# Do Systemic Antibiotics Offer Benefits to the Surgical Treatment of Peri‐Implantitis? A Systematic Review With Meta‐Analyses

**DOI:** 10.1111/jcpe.70021

**Published:** 2025-08-27

**Authors:** Georgios N. Antonoglou, Spyridon N. Papageorgiou, Ana Carrillo de Albornoz, Michael Payer, Andreas Stavropoulos

**Affiliations:** ^1^ Periodontology Unit, Centre for Host Microbiome Interactions, Faculty of Dentistry, Oral and Craniofacial Sciences King's College London London UK; ^2^ Clinic of Orthodontics and Pediatric Dentistry, Center for Dental Medicine University of Zurich Zurich Switzerland; ^3^ Etiology and Therapy of Periodontal and Peri‐Implant Diseases (ETEP) Research Group, Faculty of Dentistry, University Complutense of Madrid Madrid Spain; ^4^ Department of Oral Surgery and Orthodontics Medical University of Graz Graz Austria; ^5^ Periodontology, Faculty of Odontology University of Malmö Malmö Sweden; ^6^ Department of Periodontology Blekinge Hospital Karlskrona Sweden; ^7^ Division of Conservative Dentistry and Periodontology University Clinic of Dentistry, Medical University of Vienna Vienna Austria; ^8^ Department of Periodontology, School of Dental Medicine University of Bern Bern Switzerland

**Keywords:** clinical trials, meta‐analysis, periodontal disease, peri‐implantitis, pocket probing depth, surgical periodontal treatment, systematic review

## Abstract

**Aim:**

To assess the potential benefit of using adjunct systemic antibiotics in surgical peri‐implantitis treatment.

**Materials and Methods:**

Six databases were searched (December 2024) for randomised/non‐randomised clinical studies. After duplicate study selection, data extraction and risk‐of‐bias assessment, random‐effects meta‐analyses of odds ratios (ORs) or mean differences (MDs) and their 95% confidence intervals (CIs) were performed, followed by the analysis of certainty of evidence.

**Results:**

Seven studies (three randomised and four non‐randomised, comprising 595 patients and 1388 implants) were included. Systemic antibiotics were associated with greater short‐term treatment success (*n* = 5; OR = 2.33; 95% CI: 1.29–4.21), bone gain (*n* = 4; MD = 0.37 mm; 95% CI: –0.68 to –0.07), increased bone level stability (*n* = 3; OR = 2.73; 95% CI: 1.50–4.99), reduced bleeding on probing (*n* = 6; OR = 0.49; 95% CI: 0.31–0.78), reduced suppuration on probing (*n* = 3; OR = 0.33; 95% CI: 0.18–0.61) and increased gingival recession (*n* = 3; MD = 0.18 mm; 95% CI: 0–0.36 mm) (*p* < 0.05). Systemic antibiotics seem to benefit only implants with modified surfaces (ORs: modified 4.10 vs. turned 0.79), and event that, without long‐term benefits (≥ 3 years). Finally, one trial found that antibiotics probably increased diarrhoea risk.

**Conclusions:**

Evidence from randomised/non‐randomised studies seems to indicate that systemic antibiotics benefit surgical peri‐implantitis treatment, in the short term (1–2 years), especially for implants with a modified surface, while data on adverse effects is scarce. No substantial long‐term benefits are seen (≥ 3 years). Uncertainty still exists regarding the potential benefit of systemic antibiotics as adjunct to surgical management of peri‐implantitis.

## 1 Introduction

The prevalence of peri‐implantitis globally is estimated as 10%–22% at the implant level and 22%–45% at the patient level (Klinge et al. 2018). The main objectives of peri‐implantitis treatment include infection control, resolution of inflammation and creation of maintainable healthy conditions. Treatment can include either non‐surgical (Renvert et al. 2019) or, most commonly, also surgical measures, with the success rates varying greatly among studies (Khoury et al. 2019; Donos et al. 2023; Karlsson et al. 2023). At the same time, there is also great variation in the definition of treatment success in the various studies, which commonly include a combination of clinical signs and/or radiographic findings. In this context, the endpoint of peri‐implantitis treatment was recently defined per consensus in a European Federation of Periodontology (EFP) Workshop as (a) residual PD ≤ 5 mm with no bleeding on probing (BoP) at more than one site point, (b) no suppuration and (c) no progressive bone loss (Herrera et al. 2023). Since the pathogenesis involves a major microbiological component, systemic antibiotics have often been used as adjuncts to surgical peri‐implantitis treatment. Recent treatment guidelines, however, have been restrictive in suggesting systemic antibiotics as adjunct to surgical peri‐implantitis treatment because of the limited and inconsistent evidence regarding any potential benefits (Khoury et al. 2019; Herrera et al. 2023). This restrictive approach appears justified when considering that up to 10% of the antibiotic prescriptions globally come from dental care professionals, and clinical recommendations may thus have a significant impact on prescription practices and the overall health of the community (Ramanathan et al. 2023).

Previous reviews have assessed the effect of systemic antibiotics in surgical peri‐implantitis treatment, but they either do not include any meta‐analyses on the subject (Øen et al. 2021; Teughels et al. 2023) or are not anymore up to date (Ramanauskaite et al. 2021; Baus‐Domínguez et al. 2023; Okuma‐Oliveira et al. 2024) as new data (e.g., Carrillo de Albornoz et al. 2024; Ramanauskaite et al. 2025; Riben Grundström et al. 2024) have become recently available. It was therefore deemed appropriate that another review on this subject is warranted to include these studies, and also with the intention of acquiring individual patient data (IPD) from existing studies to enhance analyses.

Therefore, the aims of this systematic review were to critically assess the evidence from randomised clinical trials (RCTs) and non‐randomised comparative clinical studies (non‐RCTs) on adult patients treated with or without systemic antibiotics as an adjunct to surgical peri‐implantitis treatment regarding disease resolution, and also to assess the possible impact of the implant surface (turned vs. modified) on treatment success. The null hypothesis was that there is no difference in the success of surgical peri‐implantitis treatment between patients who receive systemic antibiotics and those who do not.

## 2 Materials and Methods

### 2.1 Protocol Development and Focused Question

This review's protocol was made a priori and registered in PROSPERO (CRD42019135753). And all post hoc changes are transparently reported (Appendix S1). The conduct and reporting of this review is guided by the Cochrane Handbook (Higgins et al. 2024) and the PRISMA statement (Page et al. 2021), respectively.

### 2.2 Eligibility Criteria

Based on the ‘Participants–Intervention–Comparison–Outcome–Study design (PICOS) schema, included were RCTs/non‐RCTs (S) on adult peri‐implantitis patients of any age, sex, ethnicity (P) receiving any surgical peri‐implantitis treatment with adjunct systemic antibiotics (I) compared to surgical peri‐implantitis treatment without antibiotics (C), without any limitations in language, publication year or status. The minimum follow‐up required for inclusion was arbitrarily set at 12 months.

The inclusion of non‐RCTs was judged imperative because of the small number of RCTs, to both identify the effectiveness of antibiotics and identify patient‐ or treatment‐related factors. Excluded were non‐clinical studies, case reports/series (defined as studies with < 10 patients) and studies where all/no patients received antibiotics. The pre‐planned primary outcome (O) for this review was treatment success, that is, disease resolution (however defined). Secondary outcomes were adjusted according to availability (Appendix S1) and included peri‐implant probing depth (PPD), peri‐implant radiographic bone level (RBL), BoP, suppuration on probing (SoP) and gingival recession (assessed as pre/post treatment changes or post‐treatment BoP/SoP). Any studies not reporting either the primary or any of the secondary outcomes were excluded.

### 2.3 Information Sources and Search

Six electronic databases were searched, without restrictions, from inception to December 2024 (Appendix S2), while open‐access/grey literature databases (Directory of Open Access Journals, Digital Dissertations, metaRegister of Controlled Trials, WHO, Google Scholar) and the reference/citation lists of included articles or systematic reviews were manually searched for additional studies.

### 2.4 Study Selection, Data Collection and Risk of Bias

After de‐duplication, the titles/abstracts were screened, followed by full‐text assessment against the eligibility criteria. Data extraction was performed using pre‐defined/piloted forms and communication with the authors for additional/raw data (Appendix S3). The risk of bias of RCTs and non‐RCTs was assessed with the RoB 2.0 and ROBINS‐I V2 tools, respectively. Study selection, data extraction and risk‐of‐bias assessment were performed independently by two authors (G.N.A., S.N.P.), and all discrepancies were discussed with a third author (A.S.). For details, see Appendix S1.

### 2.5 Data Analysis

As the outcome of surgical peri‐implantitis treatment is bound to be affected by patient treatment–related characteristics, a random‐effects model was a priori deemed appropriate, based on clinical/statistical reasoning (Papageorgiou 2014a), to calculate the average distribution of treatment effects using a restricted maximum likelihood variance estimator (Langan et al. 2019). Odds ratios (ORs) for binary outcomes or mean differences (MDs) for continuous outcomes with their 95% confidence intervals (CIs) were used to pool data from published papers or rearranged IPD provided by the authors (Appendix S1). The extent/impact of between‐study heterogeneity was assessed by forest plot inspection and calculation of *τ* ^2^ (absolute heterogeneity)/*I* ^2^ statistics (relative inconsistency; proportion of total variability explained by heterogeneity and not chance), while also considering the heterogeneity's direction (localisation on the forest plot) and uncertainty around heterogeneity estimates (Higgins et al. 2003).

### 2.6 Additional Analyses and Risk of Bias Across Studies

Possible heterogeneity sources were planned to be sought through random‐effects subgroup/meta‐regression analyses for meta‐analyses with five or more studies but could ultimately be performed only partly (Appendix S1). Reporting bias assessment was similarly planned but could not be carried out (Appendix S1). Separate subgroups for RCTs and non‐RCTs were included in all meta‐analyses. When statistically significant between‐subgroup differences were not observed, the overall estimate was used. When RCTs or non‐RCTs yielded different results (significant *p*‐value for subgroup effects), conclusions were based solely on RCTs. Between‐study heterogeneity was incorporated into 95% prediction intervals (PrIs) to estimate the possible effects in a future scenario (IntHout et al. 2016).

The certainty of evidence was rated using the GRADE (Grades of Recommendations, Assessment, Development and Evaluation) approach (Carrasco‐Labra et al. 2016). Forest plots were augmented with contours denoting the magnitude of the observed effects (Appendix S1) to assess heterogeneity, clinical relevance and imprecision (Papageorgiou 2014b).

A post hoc sensitivity analysis was performed by excluding an included study with a minimally invasive surgical protocol. Additionally, guidance from the Cochrane Handbook (Higgins et al. 2024) suggests that if non‐RCTs are included in meta‐analysis, those at critical risk of bias are to be excluded. Employing a more conservative approach, another post hoc sensitivity analysis was performed by excluding from the meta‐analyses non‐RCTs at either serious or critical risk of bias.

Analyses were run in R (version 4.4.1) by one author (S.N.P.) with an open dataset (Antonoglou et al. 2025). All *p*‐values were two‐sided with α = 5%, except for between‐studies/between‐subgroups heterogeneity, where α was set at 10% (Ioannidis 2008).

## 3 Results

### 3.1 Study Selection

**FIGURE 1:**
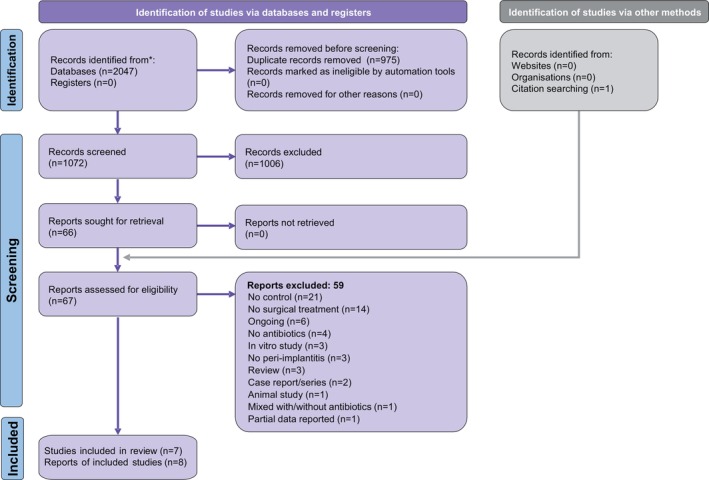
PRISMA flow diagram for the identification and selection of eligible studies.

A total of 2047 hits were retrieved by the electronic database search (Figure 1; Appendix S4). After removing duplicates, screening of titles/abstracts and adding one manually identified paper, 67 full‐text papers were checked against the eligibility criteria. In the end, eight publications (Charalampakis et al. 2011; Carcuac et al. 2016, 2017; Hallström et al. 2017; Berglundh et al. 2018; Carrillo de Albornoz et al. 2024; Ramanauskaite et al. 2025; Riben Grundström et al. 2024) pertaining to seven unique studies were included in this review. After communication attempts (Appendix S3), the full study datasets were acquired for three studies (Charalampakis et al. 2011; Hallström et al. 2017; Carrillo de Albornoz et al. 2024). Additional data or clarifications were sent for two studies (Carcuac et al. 2016; Ramanauskaite et al. 2025). No reply was received for one study (Berglundh et al. 2018) and a negative response to share data was received for another study (Riben Grundström et al. 2024).

### 3.2 Characteristics of Included Studies

Of the seven included studies, three were RCTs and four were retrospective, controlled, non‐randomised before‐and‐after (cohort) studies (Table 1). The included studies were conducted in university clinics or private practices in three countries (Germany, Spain and Sweden).

**TABLE 1:** Demographics and treatment characteristics of included studies.

Study	Design; setting; country	Patients (M/F); mean age	Smokers	Patients with SD	Implants	Modified surface	Peri‐implantitis case definition	Treatment (apart from OHI)
Berglundh et al. (2018)	rNRS; clinic; Sweden	AB: 36 (NR/NR); NR CT: 14 (NR/NR); NR	AB/CT: 20%	NR	AB/CT: 95	AB/CT: 46%	PPD ≥ 6 mm + BoP/SoP + RBL ≥ 3 mm	Apically repositioned flap, CHX MW ± AMX (750 mg 2/d × 10 d)
Carcuac et al. (2016), Carcuac et al. (2017)	RCT; Uni; Sweden	AB: 52 (15/37); 66.8 CT: 48 (20/28); 65.8	AB: 35% CT: 31%	AB: 4% CT: 6%	AB: 93 CT: 86	AB: 84% CT: 67%	PPD ≥ 6 mm + BoP/SoP + RBL ≥ 3 mm	Apically repositioned flap ± ISD (0.2% CHX) ± AMX (750 mg 2/d × 10 d)
Carrillo de Albornoz et al. (2024)^a^	rNRS; clinic; Spain	AB: 87 (30/57); 65.4 CT: 28 (9/19); 67.3	AB: 16% CT: 4%	AB: 68% CT: 75%	AB: 271 CT: 67	AB: 76% CT: 72%	PPD ≥ 6 mm + BoP/SoP + RBL ≥ 3 (at 1‐year examination)	Mucosal curettage + crestal bone exposure + peri‐implant mucosa retraction ± prosthesis removal/modification + ultrasonic/air‐polishing + + ISD (3% hydrogen peroxide) + CHX MW + AB1: MET (500 mg, 3/d × 7 d) AB2: AZT (500 mg, 1/d × 3 d) AB3: AMX (continuation of existing scheme, 7 d)
Charalampakis et al. (2011)^a^	rNRS; clinic; Sweden	AB: 155 (58/97); 67.0 CT: 38 (15/23); 66.0	AB: 36% CT: 53%	AB: 66% CT: 42%	AB: 489 CT: 111	AB: 100% CT: 100%	PPD ≥ 5 mm + BoP/SoP + RBL ≥ 1.8 mm	Open flap debridement or apically repositioned flap or regenerative surgery + ISD (NaCl/CHX/iodine) ± AB (see Appendix S5)
Riben Grundström et al. (2024)	RCT; Uni; Sweden	AB: 56 (19/37); 64.4 CT: 28 (13/15); 69.5	AB: 23% CT: 18%	AB: 43% CT: 57%	AB: 79 CT: 34	AB: 82% CT: 88%	PPD ≥ 6 mm + BOP and/or SOP + RBL ≥ 2 mm	Open flap debridement, resective surgery, ISD (3% hydrogen peroxide) + AB1: AMX (500 mg) + MET (400 mg) (3/d × 7 d) AB2: MET (400 mg) + PHE (1600 mg) (3/d × 7 d)
Hallström et al. (2017)^a^	RCT; Uni; Sweden	AB: 15 (NR^b^); 69.3 CT: 16 (NR^b^); 71.7	AB/CT: 37%	NR^b^	AB: 15 CT: 16	NR	PPD ≥ 5 mm + RBL ≥ 2–3 mm	Open flap debridement, CHX MW ± AZT (250 mg 1/d × 4 d)
Ramanauskaite et al. (2025)	rNRS; Uni; Germany	AB: 12 (6/6); 63.1 CT: 10 (4/6); 69.9	AB: 25% CT: 0%	AB: 0% CT: 0%	AB: 19 CT: 13	AB: 100% CT: 100%	BOP and/or SOP + PPD increase (from previous examination) + RBL (from baseline)	Laser/airflow + open flap debridement (Ti brush) + regenerative surgery (± membrane) + CHX MW ± AMX (500 mg, 3/d × 3 d)

Abbreviations: AB, antibiotic group; AMX, amoxicillin; ARF, apically repositioned flap; AZT, azithromycin; BoP, bleeding on probing; CHX, chlorhexidine; CT, control group; d, day; FMPS, full‐mouth plaque score; ISD, implant surface decontamination; OHI, oral hygiene instructions; MET, metronidazole; MW, mouthwash; PHE, phenoxymethylpenicillin; PPD, peri‐implant probing depth; RBL, radiographic bone level; RCT, randomised clinical trial; rNRS, retrospective non‐randomised study (comparative before‐and‐after [cohort] study); SD, systemic disease; SoP, suppuration on probing.

^a^ Raw data available.

^b^ Given only for the sample recruited sample and not for the sample ultimately analysed.

Identified studies included a total of 595 patients (median 84 patients/study), and 69.4% of them (413/595) received antibiotics. There was a higher predilection for female patients (63.2%; 325/514 from the five studies reporting gender) and the average age was 66.6 years (from the six studies reporting age). Included patients were smokers in 28.0% of the cases (167/595), with smoker prevalence ranging from 0% to 53%. Of the five studies reporting on this, 47% of included patients (243/514) had some kind of systemic disease.

A total of 1388 implants were evaluated in the 595 patients of included studies (median 1.8 implants/patient; range 1.0–3.1). Among the five studies reporting on implant surface, 74.1% (561/757) had a modified (‘roughened’) surface, while one study (Ramanauskaite et al. 2025) included only implants with a modified surface. For peri‐implantitis disease definition, PPD and RBL were used as criteria in all cases, either through certain cut‐off levels (PPD cut‐offs of 5 or 6 mm; RBL cut‐offs of 1.8–3.0 mm) or as a change from baseline. Additionally, BoP and/or SoP were used as criteria in six and seven studies, respectively (i.e., composite variable).

Surgical peri‐implantitis treatment in the identified studies included—as described in the papers—open‐flap debridement (four studies; Charalampakis et al. 2011; Hallström et al. 2017; Ramanauskaite et al. 2025; Riben Grundström et al. 2024), apically repositioned flap (two studies; Carcuac et al. 2016; Berglundh et al. 2018) or minimally invasive retraction of the peri‐implant mucosa and exposure of the crestal bone with a periodontal probe (one study; Carrillo de Albornoz et al. 2024). Mechanical implant decontamination was performed with rubber cup polishing (Berglundh et al. 2018), curettes (Carcuac et al. 2016; Hallström et al. 2017; Ramanauskaite et al. 2025; Riben Grundström et al. 2024), titanium brushes (Ramanauskaite et al. 2025), ultrasonic tips (Carrillo de Albornoz et al. 2024), air polishing (Carrillo de Albornoz et al. 2024) or was not specifically reported (Charalampakis et al. 2011). Chemical surface decontamination, in addition to mechanical implant decontamination, was performed in four of the seven studies and included chlorhexidine (CHX) (Carcuac et al. 2016), hydrogen peroxide (Carrillo de Albornoz et al. 2024; Riben Grundström et al. 2024) or one of various antiseptics (NaCl, hydrogen peroxide, CHX or iodine) (Charalampakis et al. 2011). In two studies, reconstructive surgical measures were also included (Charalampakis et al. 2011; Ramanauskaite et al. 2025), while three studies also used a resective approach to correct existing bone irregularities (Carcuac et al. 2016; Berglundh et al. 2018; Riben Grundström et al. 2024). Post‐operative CHX mouthwashes were given in four studies.

Several systemic antibiotic schemes were used, with variation in antibiotic type, dose and administration frequency/duration. Four studies used a single‐antibiotic scheme, with three of them using amoxicillin and one using azithromycin. The remaining studies did not use the same antibiotic protocol for all included patients but used two or more different antibiotic protocols, either consisting of a single antibiotic or a combination of them (Table 1; Appendix S5).

As far as outcomes are concerned (Table 2), all studies reported, except for treatment success (which was used as inclusion criterion in this review), on PPD and BoP. RBL was reported in six studies, SoP in four, gingival recession in three and clinical attachment level and bacterial load in one each. Considerable variation among studies existed regarding the composite outcome used to define peri‐implantitis treatment success, with different cut‐off values of PPDs (4.0 or 5.0 mm) and minimal or no BoP, occasionally including lack of SoP (six studies), and maximum RBL loss allowed after therapy (≤ 0.5, 1.0 or 2.0 mm) (five studies). Follow‐up durations differed substantially among the included studies. All three RCTs assessed patients at exactly ½ year and 1 year, and one of them also at 3 years post treatment; different follow‐ups existed in the non‐RCTs (mean follow‐ups 1.5–4.5 years; extending up to 13.0 years post treatment).

**TABLE 2:** Study outcomes and follow‐up of included studies.

Study	Outcome	Treatment success definition	Follo‐up (mo)
Berglundh et al. (2018)	BoP; RBL; PPD; recession; treatment success	(i) PPD ≤ 5.0 (ii) ΔRBL ≤ 0.5 (iii) PPD ≤ 5.0 + ΔRBL ≤ 0.5 (iv) no BOP (v) PPD ≤ 5.0 + no BoP (vi) ΔRBL ≤ 0.5 + no BoP (vii) PPD ≤ 5.0 + ΔRBL ≤ 0.5 + no BoP	Mean: 4.5 ± 2.1 years Range: 2.0–10.8 years
Carcuac et al. (2016), Carcuac et al. (2017)	Bacterial load; BoP; RBL; PPD; SoP; treatment success	PPD ≤ 5.0 + ΔRBL ≤ 0.5 + no BoP/SoP	At: 0.5 + 1.0 + 3.0 years
Carrillo de Albornoz et al. (2024)	BoP; RBL; PPD; recession; treatment success	(i) PPD ≤ 5.0 + np BoP/SoP + ΔRBL ≤ 0.5 (ii) PPD ≤ 5.0 + BoP ≤ 1 + ΔRBL ≤ 0.5 + no SoP (iii) PPD ≤ 5.0 + BoP ≤ 2 + ΔRBL ≤ 0.5 + no SoP	Mean: 1.5 ± 0.8 years Range: 1.0–5.0 years
Charalampakis et al. (2011)	Bacterial load; BoP; RBL; PPD; SoP; treatment success	PPD ≤ 5.0 + no BoP/SoP	Mean: 2.5 ± 1.6 years Range: 1.0–13.0 years
Riben Grundström et al. (2024)	BoP; CAL; RBL; PPD; recession; SoP; treatment success	PPD ≤ 5.0 + ΔRBL ≤ 0.5 + BoP ≤ 1.0 + no SoP	1.0 year
Hallström et al. (2017)	Bacterial load; BoP; RBL; PPD; treatment success	PPD ≤ 5.0 + ΔRBL ≤ 0.5 + no BoP/SoP	At: 0.5 + 1.0 year
Ramanauskaite et al. (2025)	BoP; SoP; PPD; treatment success	PPD ≤ 5.0 + BoP ≤ 1 + no SoP	At: 0.5 + 1.0 year

Abbreviations: BoP, bleeding on probing; mo, month; PPD, peri‐implant probing depth; RBL, radiographic bone level; SoP, supportation on probing; Δ, change.

### 3.3 Risk of Bias Within Studies

The three RCTs were at low risk of bias overall (Appendix S6). Out of the four non‐RCTs (Appendix S7), two were at moderate risk of bias, one at serious risk of bias and one at critical risk of bias. The most problematic domains were bias due to missing data (critical for one study; moderate for another study), bias due to confounding (serious for two studies) and bias due to participant selection (moderate for all four studies).

### 3.4 Data Synthesis and Certainty of Evidence

Performed meta‐analyses of published or adjusted‐for‐confounders IPD (Appendix S8) data are presented in Table 3 and Appendices S9–S18. The certainty of evidence ranged from high to low due to study design, imprecision and effect size magnitude (Table 4; Appendix S19; see Appendix S1 for details).

**TABLE 3:** Random‐effects meta‐analyses for the effect of systemic antibiotics for peri‐implantitis treatment (studies/effect estimate with 95% CIs and *p*‐value/heterogeneity estimates with 95% CIs/95% predictions for chosen analysis).

Outcome	Follow‐up (yr)	Analysis level	Randomised studies	Non‐randomised studies	*p*_SG_	Total
Tx success (short‐term)	1/1–2	Imp/Mult	2 studies^a^, ^b^ OR 2.29 (0.69, 7.64); *p* = 0.18 *τ* ^2^ 0.57 (−)/*I* ^2^ 75% (0%, 94%)	3 studies^c^, ^d^ OR 2.54 (1.20, 5.36); *p* = 0.01 *τ* ^2^ 0 (0, 29.85)/*I* ^2^ 0% (0%, 90%)	0.87	5 studies^a^, ^b^, ^c^, ^d^, ^e^ OR 2.33 (1.29, 4.21); *p* = 0.004 *τ* ^2^ 0.14 (0, 4.15)/*I* ^2^ 27% (0%, 71%) PrI 0.51, 10.68
Tx success (long‐term)	3/mixed	Imp/Mult	1 study^a^ OR 0.69 (0.32, 1.47); *p* = 0.34 *τ* ^2^—/*I* ^2^—PrI—	2 studies^c^, ^d^ OR 4.93 (1.08, 22.56); *p* = 0.04 *τ* ^2^ 0 (−)/*I* ^2^ 0% (−)	0.02*	3 studies^a^, ^c^, ^d^ OR 1.84 (0.41, 8.36); *p* = 0.43 *τ* ^2^ 1.06 (0, 48.78)/*I* ^2^ 62% (0%, 89%)
PPD change (post–pre)	1/3/mixed	Pat/Imp	3 studies^a^, ^b^, ^f^ MD –0.33 (−0.84, 0.17); *p* = 0.19 *τ* ^2^ 0 (0, 2.47)/*I* ^2^ 0% (0%, 90%)	3 studies^d^, ^e^, ^g^ MD 0 (−0.46, 0.45); *p* = 0.99 *τ* ^2^ 0 (0, 7.93)/*I* ^2^ 0% (0%, 90%)	0.34	6 studies^a^, ^b^, ^d^, ^e^, ^f^, ^g^ MD –0.15 (−0.49, 0.19); *p* = 0.38 *τ* ^2^ 0 (0, 0.77)/*I* ^2^ 0% (0%, 75%) PrI −0.63, 0.33
PPD ≥ 0.5 mm (post‐treatment)	1/mixed	Imp/Mult	2 studies^a^, ^f^ OR 0.85 (0.37, 1.94); *p* = 0.70 *τ* ^2^ 0 (−)/*I* ^2^ 0% (−) PrI−	2 studies^d^, ^e^ OR 0.19 (0.06, 0.56); *p* = 0.002 *τ* ^2^ 0 (−)/*I* ^2^ 0% (−)	0.02*	4 studies^a^, ^d^, ^e^, ^f^ OR 0.40 (0.14, 1.15); *p* = 0.08 *τ* ^2^ 0.58 (0, 8.87)/*I* ^2^ 52% (0%, 84%)
RBL change (post‐pre)	1/3/mixed	Pat/Imp	2 studies^a^, ^b^ MD −0.57 (−1.02, −0.13); *p* = 0.01 *τ* ^2^ 0.03 (−)/*I* ^2^ 24% (−)	2 studies^d^, ^g^ MD −0.16 (−0.55, 0.23); *p* = 0.41 *τ* ^2^ 0 (−)/*I* ^2^ 0% (−)	0.17	4 studies^a^, ^b^, ^d^, ^g^ MD −0.37 (−0.68, −0.07); *p* = 0.02 *τ* ^2^ 0.02 (0, 1.61)/*I* ^2^ 14% (0%, 87%) PrI −1.25, 0.50
RBL loss < 0.5 mm (post‐pre)	1/3	Imp	2 studies^a^, ^b^ OR 2.86 (1.49, 5.48); *p* = 0.001 *τ* ^2^ 0 (−)/*I* ^2^ 0% (−)	1 study^d^ OR 2.12 (0.44, 10.23); *p* = 0.35 *τ* ^2^—/*I* ^2^—	0.73	3 studies^a^, ^b^, ^d^ OR 2.73 (1.50, 4.99); *p* = 0.001 τ^2^ 0 (0, 5.48)/*I* ^2^ 0% (0%, 90%) PrI 0.06, > 100.00
BoP (post‐treatment)	1/mixed	Pat/Imp	3 studies^a^, ^b^, ^f^ OR 0.49 (0.24, 1.01); *p* = 0.05 *τ* ^2^ 0.15 (0, 32.54)/*I* ^2^ 29% (0%, 93%)	3 studies^d^, ^e^, ^g^ OR 0.41 (0.19, 0.93); *p* = 0.03 *τ* ^2^ 0 (0, 16.09)/*I* ^2^ 0% (0%, 90%)	0.63	6 studies^a^, ^b^, ^d^, ^e^, ^f^, ^g^ OR 0.49 (0.31, 0.78); *p* = 0.002 *τ* ^2^ 0.03 (0, 1.91)/*I* ^2^ 0% (0%, 75%) PrI 0.21, 1.13
SoP (post‐treatment)	1/mixed	Imp/Mult	3 studies^a^, ^b^, ^f^ OR 0.33 (0.18, 0.61); *p* < 0.001 *τ* ^2^ 0 (−)/*I* ^2^ 0% (0%, 90%) PrI 0.01, 17.66	2 studies^d^, ^e^ OR 3.87 (0.36, 41.91); *p* = 0.27 *τ* ^2^ 0 (−)/*I* ^2^ 0% (−)	0.04*	5 studies^a^, ^b^, ^d^, ^e^, ^f^ OR 0.38 (0.21, 0.70); *p* = 0.002 *τ* ^2^ 0 (0, 13.59)/*I* ^2^ 1% (0%, 80%)
Recession change (post‐pre)	1/mixed	Imp/Mult	2 studies^b^, ^f^ MD 0.14 (−0.36, 0.63); *p* = 0.59 *τ* ^2^ 0 (−)/*I* ^2^ 0% (−)	1 study^d^ MD 0.19 (0, 0.38); *P* = 0.05 *τ* ^2^—/*I* ^2^—	0.85	3 studies^b^, ^d^, ^f^ MD 0.18 (0, 0.36); *p* = 0.04 *τ* ^2^ 0 (0, 0.01)/*I* ^2^ 0% (0%, 90%) PrI −0.97, 1.33

Abbreviations: BoP, bleeding on probing; CI, confidence interval; Imp, implant; MD, mean difference; Mult, multilevel; NA, non‐applicable; OR, odds ratio; Pat, patient; PPD, pocket probing depth; PrI, random‐effects 95% prediction interval; *p*_SG_, *p* value between randomised/non‐randomised subgroups; RBL, ragiographic bone level; SoP, suppuration on probing; Tx, treatment; yr, year.

^a^ Carcuac et al. (2016), Carcuac et al. (2017).

^b^ Riben Grundström et al. (2024).

^c^ Charalampakis et al. (2011).

^d^ Carrillo de Albornoz et al. (2024).

^e^ Ramanauskaite et al. (2025).

^f^ Hallström et al. (2017).

^g^ Berglundh et al. (2018).

* Statistically significant differences between subgroups for randomised and non‐randomised studies (*p* < 0.10).

**TABLE 4:** Summary of findings table according to the GRADE approach based on results of randomised trials.

		Anticipated absolute effects (95% CI)				
Outcome Studies (implants)	Relative effects (95% CI)	Control group^a^	Antibiotic group	Difference with antibiotics	Quality of the evidence (GRADE)	Plain language
Treatment success (short‐term) 5 RCTs/non‐RCTs (718 implants)	OR 2.33 (1.29, 4.21)	455 per 1000	660 per 1000	206 implants more (64–324 more)	⨁⨁◯◯ Low^b^	Antibiotics might moderately increase short‐term treatment success
Treatment success (long‐term) 1 RCT (121 implants)	OR 0.69 (0.32, 1.47)	377 per 1000	295 per 1000	82 implants less (215 less to 94 more)	⨁⨁◯◯ Low^c^, ^d^	Antibiotics might not improve long‐term treatment success
PPD change (post‐pre) 6 RCTs/non‐RCTs (583 implants)	—	2.32 mm reduction	—	0.15 mm greater reduction (0.49 more to 0.19 less)	⨁⨁◯◯ Low^b^, ^e^	Antibiotics might not affect PPD
PPD ≥ 5.0 mm (post‐treatment) 2 RCTs (430 implants)	OR 0.85 (0.37, 1.94)	206 per 1000	181 per 1000	25 implants less (117 less to 129 more)	⨁⨁⨁⨁ Ligh^c^	Antibiotics do not reduce number of implants with PPD ≥ 5.0 mm
RBL change (post‐pre) 4 RCTs/non‐RCTs (546 implants)	—	0.21 mm gain	—	0.37 mm more gain (0.07–0.68 mm gain)	⨁⨁◯◯ Low^b^	Antibiotics might minimally improve RBL gain post‐treatment
RBL loss ≤ 0.5 mm (post‐pre) 3 RCTs/non‐RCTs (430 implants)	OR 2.73 (1.50, 4.99)	787 per 1000	910 per 1000	123 implants more (60 to 161 more)	⨁⨁⨁◯ Moderate^b^, ^f^	Antibiotics probably greatly reduce the possibility of RBL loss ≤ 0.5 mm post treatment
BoP (post‐treatment) 6 RCTs/non‐RCTs (643 implants)	OR 0.49 (0.31, 0.78)	502 per 1000	329 per 1000	173 implants less (63 to 277 less)	⨁⨁◯◯ Low^b^	Antibiotics might moderately reduce BoP post treatment
SoP (post‐treatment) 3 RCTs (604 implants)	OR 0.33 (0.18, 0.61)	271 per 1000	109 per 1000	162 implants less (86–209 less)	⨁⨁⨁⨁ High^c^, ^g^	Antibiotics moderately reduce SoP post treatment
Recession change (post‐pre) 3 RCTs/non‐RCTs (376 implants)	—	0.40 mm loss	—	0.18 mm more (0–0.36 mm more)	⨁⨁◯◯ Low^b^	Antibiotics might increase gingival recession post treatment
Adverse effect: diarrhea^h^ 1 RCT (104 implants)	Not estimable	0 per 1000	212 per 1000	212 implants more (95% CI not estimable)	⨁⨁⨁◯ Moderate^c^, ^d^	Antibiotics probably increase the risk of (sometimes prolonged) diarrhoea

*Note*: Intervention: adjunct use of systemic antibiotics for the surgical treatment of peri‐implantitis compared to surgical treatment without antibiotics. Population: adult patients with at least one dental implant with peri‐implantitis. Setting: university clinics and private clinics (Germany, Spain and Sweden).

Abbreviations: BoP, bleeding on probing; CI, confidence interval; GRADE, Grading of Recommendations Assessment, Development and Evaluation; Non‐RCT, non‐randomised comparative clinical trial; PPD, peri‐implant probing depth; RBL, radiographic bone level; RCT, randomise clinical trial; SoP, suppuration on probing.

^a^ Response in the control group is based on the response of included studies (or random‐effects meta‐analysis of the control response).

^b^ Starts from ‘low’ since RCTs and non‐RCTs are combined.

^c^ Starts from ‘high’, since only RCTs are included.

^d^ Downgraded by two levels for severe imprecision due to the inclusion of an inadequate sample and very wide CIs.

^e^ Inconsistency found with heterogeneous estimates on both sides of the forest plot; but effects are minor in all instances with wide 95% CIs; judged as lack of an effect and therefore no downgrade was performed.

^f^ Upgraded by one level, since a potentially large effect was seen and no reasons to downgrade were seen.

^g^ Potential reason to upgrade due to large effect, but rating already at ‘high’.

^h^ One trial reported non‐serious adverse events of mild intensity; reversible gastrointestinal discomfort was restricted to the antibiotics group; diarrhoea (sometimes prolonged) being the most common adverse effect and is the sole adverse effect included in this GRADE Summary of Findings table.

**FIGURE 2:**
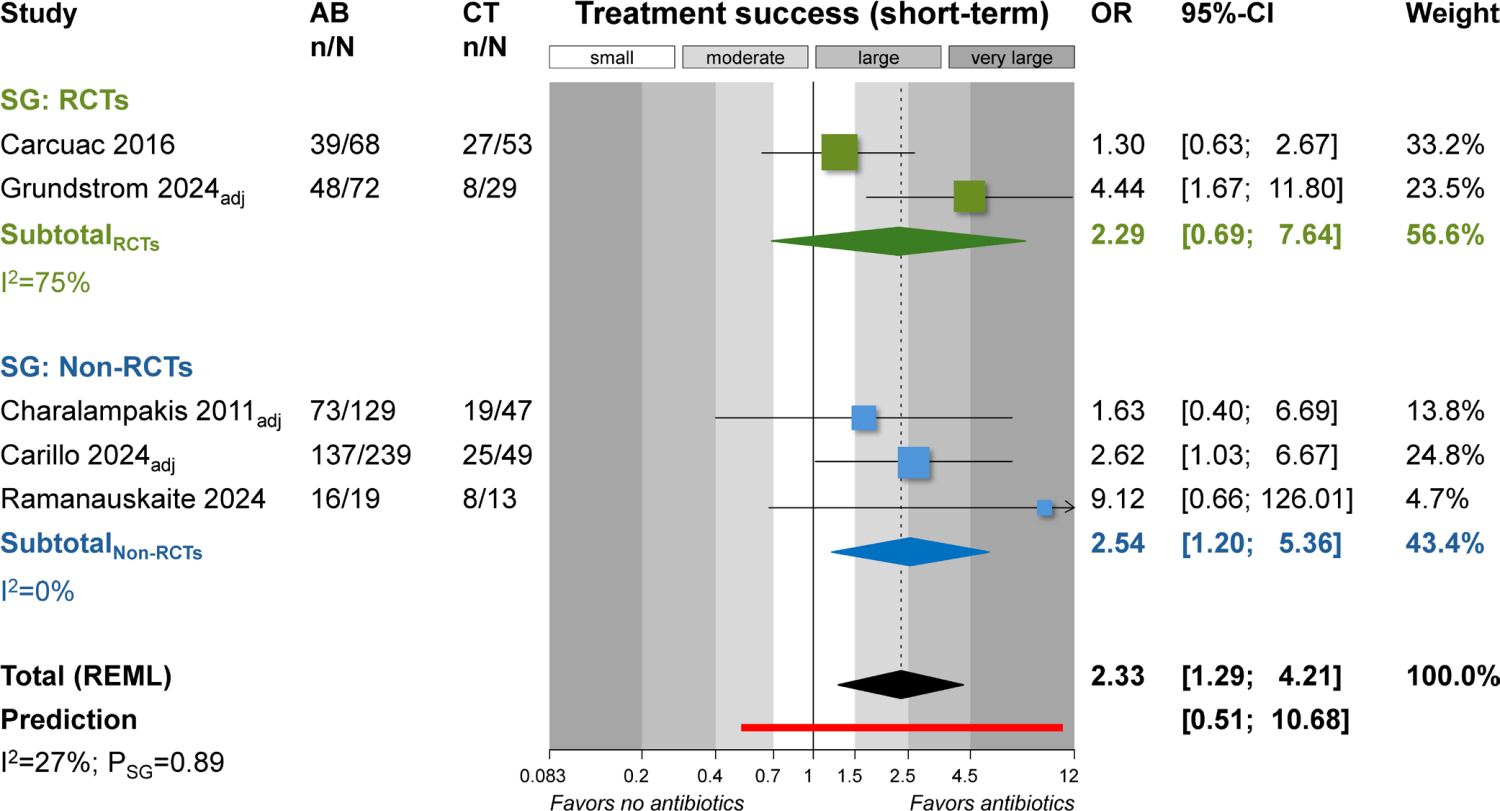
Meta‐analysis on the effect of antibiotics on treatment success in the short term. AB, antibiotics group; adj, adjusted for confounders estimates used; CI, confidence interval; CT, control group (no antibiotics); n/N, events/sample; non‐RCT, non‐randomised comparative clinical trial; OR, odds ratio; *p*_SG_, *p‐*value for differences between subgroups; RCT, randomised clinical trial; SG, subgroup; REML, restricted maximum likelihood model.

Adjunct use of systemic antibiotics was associated with increased odds for treatment success in the short term (1–2 years) compared to no antibiotics (*n* = 5; OR = 2.33; 95% CI: 1.29–4.21; *p* = 0.004; Figure 2; Table 3; ‘low’ GRADE). In the long term (≥ 3 years post treatment), RCTs/non‐RCTs gave conflicting results (Appendix S9) and, based on one RCT, antibiotics probably do not improve success (OR = 0.69; 95% CI: 0.32–1.47; *p* = 0.34; ‘low’ GRADE). The effect of systemic antibiotics on the average PPD change during treatment was minimal and probably of no clinical relevance (*n* = 6; MD = −0.15 mm; 95% CI: –0.49 to 0.19 mm; *p* = 0.38; Appendix S10). Similarly, antibiotic prescription did not reduce the number of implants with PPD ≥ 5.0 mm post treatment (*p* = 0.70; Appendix S11; ‘high’ GRADE). Different results were seen between RCTs and non‐RCTs, which was due to the RCT of Carcuac et al. (2016) not finding any benefit (OR = 0.99), whereas the remaining three studies found benefits (ORs 0.14–0.39). Four RCTs/non‐RCTs indicated that antibiotics might minimally improve RBL (bone gain) post treatment (MD = −0.37 mm; 95% CI: –0.68 to –0.07 mm; *p* = 0.02; Appendix S12; ‘low’ GRADE). Antibiotics might greatly reduce the possibility of RBL loss of ≤ 0.5 mm post treatment (*n* = 3; OR = 2.73; 95% CI: 1.50–4.99; *p* = 0.001; Appendix S13; ‘moderate’ GRADE). Additionally, systemic antibiotics might moderately reduce BoP post treatment (*n* = 6; OR = 0.49; 95% CI: 0.31–0.78; *p* = 0.002; Appendix S14; ‘low’ GRADE). However, systemic antibiotics certainly reduced SoP around the implant post treatment (*n* = 3; OR = 0.33; 95% CI: 0.18–0.61; *p* < 0.001; Appendix S15; ‘high’ GRADE). Moreover, antibiotics might minimally increase gingival recession post treatment (*n* = 3; MD = 0.18 mm; 95% CI: 0–0.36 mm; *p* = 0.04; Appendix S16).

### 3.5 Additional Analyses

**FIGURE 3:**
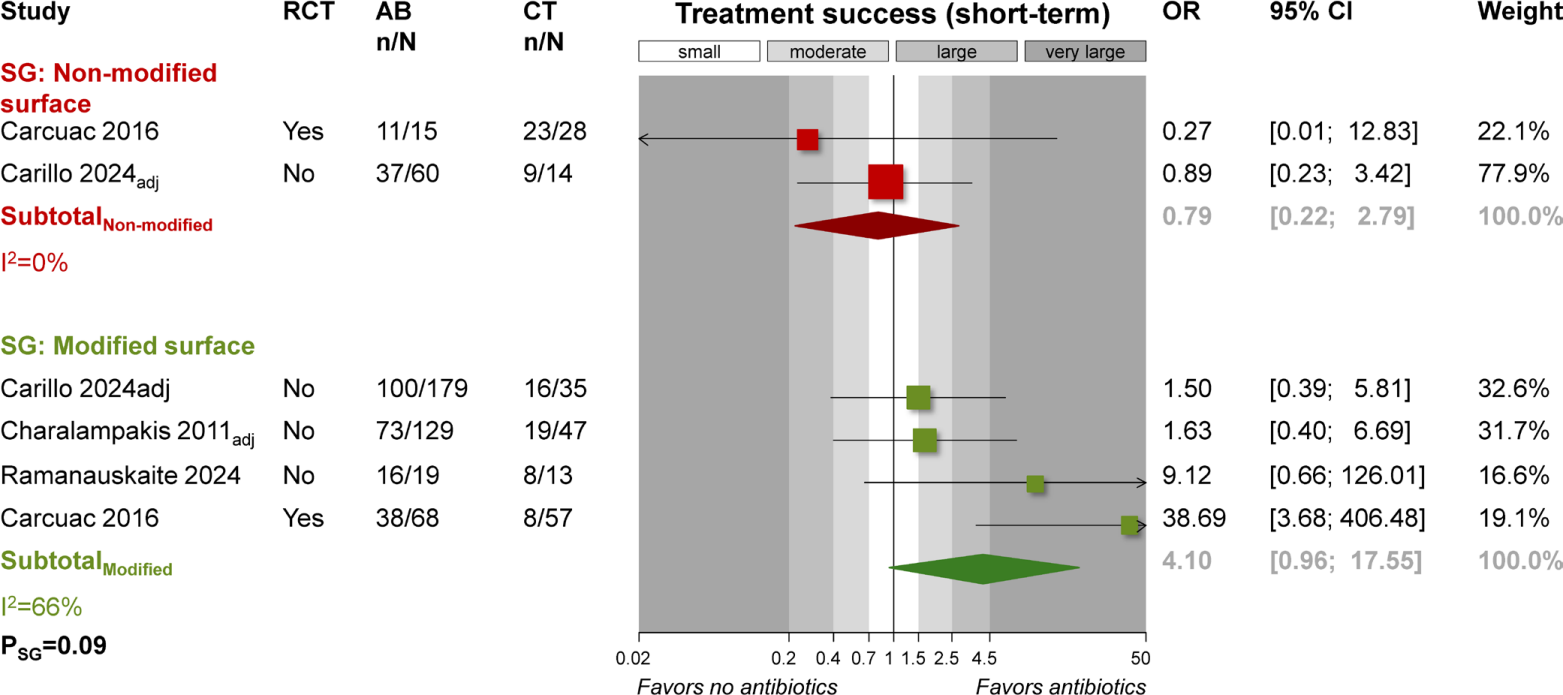
Meta‐analysis on the effect of antibiotics on treatment success in the short term according to implant surface subgroups. AB, antibiotics group; adj, adjusted for confounders estimates used; CI, confidence interval; CT, control group (no antibiotics); n/N, events/sample; OR, odds ratio; *p*_SG_, *p*‐value for differences between subgroups; RCT, randomised clinical trial; SG, subgroup; REML, restricted maximum likelihood model.

Stratified meta‐analysis according to implant surface (Figure 3) indicated significant between‐subgroup short‐term treatment success differences (*p* = 0.09) and systemic antibiotics benefited implants with modified (*n* = 4; OR = 4.10; 95% CI: 0.96–17.55) rather than non‐modified surface (*n* = 2; OR = 0.79; 95% CI: 0.22–2.79; see Appendix S1 for details).

Since Carrillo de Albornoz et al. (2024) used a minimally invasive surgical approach that differed from others, a sensitivity analysis was performed by excluding this study (Appendix S17), but no considerable differences were seen.

Sensitivity analysis without non‐RCTs with serious/critical risk of bias (Charalampakis et al. 2011; Berglundh et al. 2018) showed similar findings (Appendix S18).

### 3.6 Adverse Effects

Riben Grundström et al. (2024) reported on adverse effects, which included non‐serious adverse events of mild intensity. Specific adverse effects were restricted to the antibiotics group: diarrhoea (21.2%), nausea (9.6%), abdominal pain (5.8%), flatulence (3.8%), headache (3.8%) and allergy/bad taste/heartburn/haematoma/panic attack (1.9% each). Other adverse effects (pain/fatigue/skin rash/swelling/itching/sinus problems/sublingual caruncle obstruction) were reported in both antibiotic and control groups. Eleven patients complained of diarrhoea; patients receiving phenoxymethylpenicillin and metronidazole were more affected than those receiving amoxicillin and metronidazole (34.6% [9/26] vs. 7.7% [2/26], respectively; *p* = 0.03), and two patients reported prolonged symptoms for up to 2 weeks (in both antibiotic groups). Only diarrhoea was included in the GRADE analysis, which showed that antibiotics probably increase the risk of (sometimes prolonged) diarrhoea (Table 4; ‘moderate’ GRADE).

## 4 Discussion

### 4.1 Evidence in Context

The present systematic review critically appraises RCTs/non‐RCTs comparing surgical peri‐implantitis treatment with or without adjunct use of systemic antibiotics. Evidence from seven studies, which included 1388 implants placed in 595 patients, indicated that systemic antibiotics were associated with increased short‐term treatment success (1–2 years post surgery). This supports earlier findings from the study of Carcuac et al. (2017) (included herein), who reported that systemic antibiotics were associated with fewer implants removed in the 3 years following surgical peri‐implantitis treatment (OR 0.10; 95% CI: 0.02–0.41; *p* = 0.002). Systemic antibiotics were likewise associated with reduced bone loss, BoP and SoP. However, when considering whether systemic antibiotics should be prescribed, the potential public health threat of antimicrobial resistance, which can be caused by suboptimal antibiotic use, should also be taken into account (Frieri et al. 2017; Thompson et al. 2019).

Systemic antibiotics were found to be associated with increased success of surgical peri‐implantitis treatment of implants with a modified surface but not of implants with a turned surface (Figure 3). This supports the notion of a significant interaction between systemic antibiotics and the implant surface, as previously suggested (Carcuac et al. 2016). It is important to state that the rate of treatment success in the control (no antibiotics) group seems to be different for implants with modified and turned surfaces. Explorative post hoc meta‐analysis of proportions from the non‐antibiotic groups in Figure 3 indicated that implants with turned surfaces had significantly (*p* = 0.001) higher treatment success rates (two studies; pooled proportion 76.2%; 95% CI: 3.1%–99.7%) compared to implants with modified surfaces (four studies; pooled proportion 36.9%; 95% CI: 13.7%–68.2%). These results might be explained by the increased difficulty of decontaminating modified implant surfaces, as has been shown in laboratory studies (Kubasiewicz‐Ross et al. 2020; Stuani et al. 2021; Hui et al. 2021; Stavropoulos et al. 2025) as well as the higher chances of recontamination of such surfaces. In fact, it has been reported that implants with modified surfaces—although do not seem to be associated with increased incidence of peri‐implantitis (Stavropoulos et al. 2021)—present with increased risk (OR 5.07; *p* < 0.001) for recurrence/progression in the 5 years after surgical peri‐implantitis treatment compared with implants with turned surfaces (Carcuac et al. 2020). Therefore, based on available evidence, it would make sense that systemic antibiotics are prescribed—assessed on a case‐by‐case basis—or implants with modified surfaces but not for implants with turned surfaces. In this context, the vast majority of currently available implant systems feature a modified surface, and systemic antibiotics may be unnecessary in cases treated with implantoplasty (Stavropoulos et al. 2019).

Great variability was seen in the antibiotic protocols used among the included studies (Table 1), which pertained to the antibiotic chosen, whether this was used alone or combined with others, dosage, consumption frequency (ranging from one to three times per day) and duration of antibiotic administration (ranging from 3 to 7 days). Antibiotic regimens with prolonged duration (7–10 days) might be associated with side effects of the gastrointestinal tract including antibiotic‐associated diarrhoea (Hurley and Nguyen 2002). For example, Riben Grundström et al. (2024) reported the occurrence of diarrhoea in about 21.2% of the patients treated with antibiotics, while no patient in the control group had diarrhoea. Furthermore, variability in the penetration of the different antibiotic molecules to the infected peri‐implant tissue is largely unknown (Avila‐Ortiz et al. 2020). Nevertheless, direct comparisons between different antibiotic schemes are limited. Riben Grundström et al. (2024) found no significant difference between amoxicillin with metronidazole and phenoxymethylpenicillin with metronidazole (*p* > 0.05). On the other hand, explorative re‐analysis of the data provided by Carrillo de Albornoz et al. (2024) indicated that compared to the control group, the antibiotic group had greater treatment success with azithromycin (OR = 3.55) than with metronidazole (OR = 1.82) or amoxicillin (OR = 1.79).

In summary, further experimental data is needed before robust recommendations on the optimal antibiotic regimen can be made. It is important to consider that the antibiotics used for periodontitis may not necessarily be appropriate for the surgical treatment of peri‐implantitis (Kotsakis and Olmedo 2021).

Even though systemic antibiotics were found to be associated with improved short‐term treatment success (1–2 years), this effect seemed to disappear in the long term (≥ 3 years), indicating the increased complexity of the clinical problem in question. Other factors like post‐operative RBL, residual deep (≥ 6.0 mm) post‐operative PPDs and rough (modified) implant surface seem to be associated with long‐term recurrence or progression of peri‐implantitis (Carcuac et al. 2020). This seems to be logical, because a single short‐term administration (3–10 days) of post‐operative antibiotics cannot be expected to have a lasting influence on peri‐implant health, without a permanent change in the conditions that initiated and/or promoted the peri‐implant infection and an appropriate supportive peri‐implant care protocol, tailored to the patient's risk profile (Karlsson et al. 2023; Stiesch et al. 2023). In this context, schemes of shorter duration might be preferable, potentially limiting post‐operative antibiotic‐related complications (diarrhoea) and the risk of antibiotic resistance.

### 4.2 Limitations

This review has several limitations. For one, there were few RCTs available, and non‐RCTs were also included. This factor, together with various methodological issues (lack of pre‐registration, unclear patient selection, lack of blinding, lack of a priori sample size calculations, no open data provision) might have introduced bias (Papageorgiou et al. 2015, 2019; Papageorgiou and Cobourne 2018). Additionally, there was great variation among the included studies regarding peri‐implantitis case definition or how treatment success was defined. Recently, the endpoint of peri‐implantitis treatment was defined as residual PD ≤ 5 mm with no BOP at more than one site point, no suppuration and no progressive bone loss (Herrera et al. 2023), and adoption of this definition in the future would increase comparability among studies. In the present review, we aimed to use provided IPD to re‐define post hoc the outcome of treatment success for older studies not using the S3 definition. However, this was not possible because only one author group provided the full dataset and that study already used the composite outcome suggested in the EFP S3 guideline (Carrillo de Albornoz et al. 2024). All meta‐analyses in the present work were based almost exclusively on small studies (< 100 implants/group), which may affect their precision (Cappelleri et al. 1996). Furthermore, incomplete reporting of results/potential confounders, lack of open dataset availability, lack of data on anatomical variations with regard to tissue characteristics, lack of data on fixture–prosthesis connection and unsuccessful data‐sharing attempts precluded the conduct of many pre‐planned analyses (including proper handling of data reported at the patient and implant level [Table 3]). Even though it was our intent to base our conclusions on an intention‐to‐treat protocol, this was not available for non‐RCTs (and some outcomes of RCTs) using a per‐protocol analysis and, as such, this might have introduced selection bias (Molero‐Calafell et al. 2024). Additionally, various surgical protocols and antibiotic protocols were used in the included studies and this introduces additional heterogeneity. Finally, the outcome of long‐term treatment success used in this review might be more appropriately termed with re‐evaluation endpoints or disease recurrence and analysed separately from short‐term (1–2 years post treatment) endpoints.

## 5 Conclusions

Evidence from randomised/non‐randomised studies seem to indicates that systemic antibiotics benefit surgical peri‐implantitis treatment, in the short term (1–2 years), especially for implants with modified surfaces, while data on adverse effects is scarce. No substantial long‐term benefits are seen (≥ 3 years), and uncertainty still exists regarding the potential benefit of systemic antibiotics as adjuncts to surgical management of peri‐implantitis.

## Author Contributions

Conceptualisation: Andreas Stavropoulos. Organisation, execution and data collection: Georgios Antonoglou, Spyridon Papageorgiou, Ana Carrillo de Albornoz, Michael Payer and Andreas Stavropoulos. Data analysis and interpretation: Georgios Antonoglou and Spyridon Papageorgiou. Manuscript drafting and revision: Georgios Antonoglou, Spyridon Papageorgiou, Ana Carrillo de Albornoz, Michael Payer and Andreas Stavropoulos.

## Ethics Statement

The authors have nothing to report.

## Conflicts of Interest

The authors declare no conflicts of interest.

## Supporting information


**Data S1:** Supporting information.

## Data Availability

The data that support the findings of this study are openly available in Zenodo at https://doi.org/10.5281/zenodo.10039892.
