# Supplementary material for: Do Systemic Antibiotics Offer Benefits to the Surgical Treatment of Peri‐Implantitis? A Systematic Review With Meta‐Analyses
Source: J Clin Periodontol. 2025 Aug 27;52(12):1746–59. doi: 10.1111/jcpe.70021 (PMC12605830; doi:10.1111/jcpe.70021)
Supplement: Supplementary file 1 — Data S1: Supporting information. [file JCPE-52-1746-s001.docx]

**Do systemic antibiotics offer benefits to the surgical treatment of peri-implantitis? A systematic review with meta-analyses**

**Supporting information**

**Appendix 1.** Additional details on review methods and post hoc deviations from the review protocol.

**Additional review details**

- The literature search met all recommendations (performed in duplicate, >2 databases, grey literature, no language restriction) set by Faggion et al. (2012). All the files containing the reference details and abstracts were stored in Endnote for de-duplication. Selected databases considered grey literature databases (Mahood et al. 2014) did not allow for the recording of the reference lists in separate files and were therefore searched manually. The search queries on the previous were modified to allow the yielding of the maximum number of hits.
- Results of pairwise meta-analyses were presented in contour-enhanced forest plots illustrating the magnitude of observed effects (Papageorgiou 2014). This helps to assess the precision, heterogeneity, and clinical relevance of observed effects. For odds ratios, conventional effects of 1.5, 2.5, and 4.5 (or inversely, 0.7, 0.4, and 0.2) were used as cut-off points to denote small (trivial), moderate, large, and very large effects. For continuous outcomes, the average baseline Standard Deviation (SD) in the pre-treatment control group response was calculated from the included of each outcome. Forest plots of continuous outcomes were enhanced by using 0.5, 1.0, and 2.0 SDs of the control group to denote small, moderate, large, and very large effects. The average baseline control group SD was calculated to be: (i) 1.45 mm for PPD change, (ii) 2.05 mm for RBL change, (iii) and 1.89 mm for gingival recession change.
- Individual Patient Data (IPD) was provided for 3 studies: Carrillo de Albornoz et al. (2024), Charalampakis et al. (2011), and Hallström et al. (2017). Their data was analyzed with generalized linear models that accounted for within-patient clustering through the use of robust standard errors (except in the case of the Hallström 2017 study that it was not needed, as only one implant per patient was included). For the Carrillo de Albornoz 2024 and the Charalampakis 2011 studies efforts were made to identify possible confounding factors to the effect of systemic antibiotics for each outcome separately. As such, for each outcome, separate models were built by adding each potential confounder available in the dataset the base model (including antibiotics and patient clustering) one at a time and measuring the change in the estimate (unstandardized regression coefficient or log odds ratio). The change-in-estimate method (Greenland and Pearce 2015) was used, with a 10% cut-off. In case an adequate number of implants were included, all identified confounders were included in the final model and accounted for. In case of a limited sample size, the most strong confounders (having the largest change-in-estimate) were selected—with the final number of confounders added being dictated by the number of implants analyzed.
- The composite outcomes of “PPD ≥ 5.0 mm post-treatment” and “RBL loss ≤ 0.5 mm” were calculated from available dataset, if possible, even though these were not always used in the published papers of these studies, as other studies had reported these outcomes and could be used in meta-analyses.
- For calculations of odds ratios, a continuity correction was applied to studies with a zero cell count, as this method has showed the best performance for the odds ratio in a simulation study under the random effects model (Weber et al. 2020).

**Supplement to 2.4 | Study selection, data collection, and risk of bias**

After de-duplication, two authors (GNA/SNP) screened the titles/abstracts of retrieved hits to exclude obviously inappropriate studies, before moving to full-text assessment. Any differences between the two reviewers were resolved by discussion with another author (AS).

Data extraction was performed independently by two authors (GNA/SNP) with the same way to resolve discrepancies using pre‐defined/piloted forms covering: (a) study characteristics (design, clinical setting, country); (b) patient characteristics (age, sex, smoking, systemic disease); (c) implant characteristics (implant number; implant surface, i.e, turned, modified); (d) peri-implantitis definition; (e) treatment approach; (f) outcomes assessed; (g) definition of treatment success, and (h) follow-up duration. Data was extracted from published reports, while corresponding authors of included studies were contacted to request additional data or their studies’ full dataset (Appendix 3).

The risk of bias of RCTs/non-RCTs was assessed with the RoB 2.0 (Sterne et al. 2019)/ROBINS-I V2 (‘Risk Of Bias In Non-randomized Studies of Interventions’) tool (https://www.riskofbias.info/welcome/robins-i-v2), respectively. All studies were appraised independently by two authors (GNA/SNP) with any differences discussed with a third author (AS).

**Supplement to 3.4 | Data synthesis**

Data synthesis was based on results reported in the published papers, on additional aggregate data received from authors (Carcuac et al. 2016; Ramanauskaite et al. 2024), and on re-arrangement of IPD provided by authors (Charalampakis et al. 2011; Hallström et al. 2017; Carrillo de Albornoz et al. 2024). For the latter, adjusted estimates taking within-patient clustering and controlling for selected confounders were calculated and used (Appendix 8). Meta-analyses on the effect of systemic antibiotics are shown in Table 3. For each outcome, RCTs/non-RCTs were grouped/analyzed separately. When no significant differences between RCTs/non-RCTs subgroups are seen (as was the case for the outcomes of short-term treatment success, PPD change, RBL change, RBL loss ≤ 0.5 mm, BoP post-treatment, and gingival recession change), the pooled results were used for the analysis. In case of significant differences between subgroups (which were observed for the outcomes of long-term treatment success, PPD post-treatment ≥ 5.0 mm, and SoP post-treatment), only RCTs were used to inform clinical recommendations.

**Supplement to 3.5 | Additional analyses**

The effect of implant surface on peri-implantitis treatment could be only partly analyzed due to the limited data availability. Carcuac et al. (2016) reported in-paper separate effects of systemic antibiotics according to implant surface (modified versus non-modified), while Ramanauskaite et al. (2024) included only implants with a modified surface. The provided IPD data from Charalampakis et al. (2011) and Carrillo et al. (2024) enabled calculation of separate effects of systemic antibiotics according to implant surface, which were not reported in their original paper. Stratified meta-analysis according to implant surface (Figure 3) indicated that a statistically significant difference in short-term treatment success existed between subgroups (P=0.09) and systemic antibiotics benefited implants with modified surface (4 studies; OR 4.10; 95% CI 0.96 to 17.55) rather than implants with non-modified surface (2 studies; OR 0.79; 95% CI 0.22 to 2.79). Other subgroup analyses were planned in-protocol but could not be conducted (Appendix 1).

Since one study (Carrillo et al. 2024) used a minimally-invasive surgical approach that differed from others, a sensitivity analysis was performed by excluding this study (Appendix 17). No considerable differences were seen compared to the original analysis, apart from the reduced statistical power that was seen due to the smaller number of studies included.

Sensitivity analysis by excluding non-RCTs with serious or critical risk of bias (Charalampakis et al. 2011; Berglundh et al. 2018) showed similar findings to the main analysis (Appendix 18). The same RCTs/non-RCTs differences were found, similar effects were statistically significant, and the effect sizes in all instances were very close to the original analysis.

**Supplement to 3.5.4 | Quality of evidence**

The certainty of evidence with the GRADE approach was judged using either all studies or only RCTs (depending on potential RCTs/non-RCTs differences). The certainty of evidence ranged from high to low due to the inclusion of non-RCTs, with no obvious reasons for downgrade (Table 4; Appendix 19). One exception was treatment success in the long-term, where GRADE was moderate, due to imprecision from the inclusion of only a single RCT. For post-treatment PPD changes, heterogenous results in both sides of the forest plot were seen but were in all instances of very small magnitude and this was interpreted as statistical noise.

**Deviations from protocol**

- The primary outcome remained as was in the originally submitted protocol. The inclusion of secondary outcomes was adapted post hoc according to what data was reported from identified eligible studies. The secondary outcomes of Peri-implant Probing Depth (PPD), Radiographic Bone Level (RBL), and Bleeding on Probing (BoP) were included in the original protocol. The outcomes of Suppuration on Probing (SoP) and gingival recession were added post hoc, as they were often reported in included studies.
- Initially, subgroup / meta-regression analyses were planned to be conducted according to patient age, sex, type of experimental procedure, operator’s experience, and definition of success. Ultimately, these data were not adequately reported to perform such analyses and most authors did not provide their full dataset (even after requests were made). Therefore, these analyses could not be conducted.
- Robustness of the results was planned to be checked with sensitivity analyses based on (i) inclusion/exclusion of trials with methodological shortcomings, (ii) inclusion/exclusion of non-randomized trials (iii) improvement of the GRADE classification, and (iv) inclusion/exclusion of large-scale studies. Ultimately, only sensitivity analysis by separating randomized / non-randomized studies could be performed with the limited data that was available, while only one study had large groups (>100 implants per antibiotics / control group).
- Reporting biases (including small-study effects and the possibility of publication bias) were planned to be assessed for meta-analyses with at least 10 studies. Ultimately, no such meta-analysis existed and these analyses were abandoned.

**References for Appendix 1**

Carrillo de Albornoz, A., E. Montero, A. Alonso-Español, M. Sanz, and I. Sanz-Sánchez. 2024. Treatment of peri-implantitis with a flapless surgical access combined with implant surface decontamination and adjunctive systemic antibiotics: A retrospective case series study. *Journal of Clinical Periodontology* 51, no. 8: 968–980. https://doi.org/10.1111/jcpe.13993.

Charalampakis, G., P. Rabe, A. Leonhardt, and G. Dahlén. 2011. A follow-up study of peri-implantitis cases after treatment. *Journal of Clinical Periodontology* 38, no. 9: 864–871. https://doi.org/10.1111/j.1600-051X.2011.01759.x..

Faggion, C. M. Jr., M. A. Atieh, and S. Park. 2013. Search strategies in systematic reviews in periodontology and implant dentistry. *Journal of Clinical Periodontology* 40, no. 9: 883–888. https://doi.org/10.1111/jcpe.12132.

Hallström, H., G. R. Persson, S. Lindgren, and S. Renvert. 2017. Open flap debridement of peri-implantitis with or without adjunctive systemic antibiotics: A randomized clinical trial. *Journal of Clinical Periodontology* 44, no. 12: 1285–1293. https://doi.org/10.1111/jcpe.12805.

Greenland, S., and N. Pearce. 2015. Statistical foundations for model-based adjustments. *Annual Review of Public Health* 18, 36:89–108. https://doi.org/10.1146/annurev-publhealth-031914-122559.

Weber, F., G. Knapp, K. Ickstadt, G. Kundt, and Ä. Glass. 2020. Zero-cell corrections in random-effects meta-analyses. *Research Synthesis Methods* 11, 6: 913–919. https://doi.org/10.1002/jrsm.1460.

Mahood, Q., D. Van Eerd, and E. Irvin. 2014. Searching for grey literature for systematic reviews: challenges and benefits. *Research Synthesis Methods* 2014;5(3):221-34. https://doi.org/10.1002/jrsm.1106.

Papageorgiou, S. N. 2014. Meta-analysis for orthodontists: Part II--Is all that glitters gold? *Journal of Orthodontics* 41, no. 4: 327–336. https://doi.org/10.1179/1465313314Y.0000000110.

**Appendix 2.** Literature searches with hits in each database (last search date December, 2024).

| **Database** | **Search query** | **Limits** | **Hits** |
| --- | --- | --- | --- |
| MEDLINE  searched via PubMed | (Dent* OR dental OR periodont*) AND (“peri-implant disease” OR “periimplant disease” OR “peri-implant infection” OR “periimplant infection” OR “mucositis” (MeSH) OR “peri-implant mucositis” OR “periimplant mucositis” OR “Periimplantitis” (MeSH) OR “peri-implantitis”) AND (“treatment” OR “surgical treatment” OR “resective treatment” OR “surgical therapy” OR “resective therapy” OR “reconstructive therapy” OR “antibiotic treatment” OR “adjunctive treatment” OR “antibiotic therapy” OR “adjunctive therapy” OR “nonsurgical treatment” OR “non-surgical treatment” OR “regenerative treatment” OR “augmentative treatment” OR “reconstructive treatment” OR “regenerative therapy” OR “augmentative therapy”) AND (longitudinal study OR randomised controlled study OR comparative study OR clinical trial) NOT (“photodynamic treatment” OR “photodynamic therapy” OR “antiseptic therapy” OR “antiseptic treatment” OR “local antibiotic*” OR “probiotic” OR systematic review*[tiab] OR systematic literature[tiab] OR meta-analysis[tiab] OR meta-analyses[tiab] OR in vitro[tiab] OR animal*[tiab]) | - | 419 |
| Scopus | ( dent* OR dental OR periodont* ) AND ( "peri-implant disease" OR "periimplant disease" OR "peri-implant infection" OR "periimplant infection" OR "mucositis" ( mesh ) OR "peri-implant mucositis" OR "periimplant mucositis" OR "Periimplantitis" ( mesh ) OR "peri-implantitis" ) AND ( "treatment" OR "surgical treatment" OR "resective treatment" OR "surgical therapy" OR "resective therapy" OR "reconstructive therapy" OR "antibiotic treatment" OR "adjunctive treatment" OR "antibiotic therapy" OR "adjunctive therapy" OR "nonsurgical treatment" OR "non-surgical treatment" OR "regenerative treatment" OR "augmentative treatment" OR "reconstructive treatment" OR "regenerative therapy" OR "augmentative therapy" ) AND ( longitudinal AND study OR randomised AND controlled AND study OR comparative AND study OR clinical AND trial ) AND NOT ( "photodynamic treatment" OR "photodynamic therapy" OR "antiseptic therapy" OR "antiseptic treatment" OR "local antibiotic*" OR "probiotic" OR systematic AND review* OR systematic AND literature OR meta-analysis OR meta-analyses OR in AND vitro OR animal*) AND ( not INDEX ( medline ) ) AND ( LIMIT-TO ( DOCTYPE , "ar" ) ) AND ( LIMIT-TO ( SUBJAREA , "DENT" ) ) | - | 372 |
| Thomson Reuters Web of Science | (Dent* OR dental OR periodont*) AND (“peri-implant disease” OR “periimplant disease” OR “peri-implant infection” OR “periimplant infection” OR “mucositis” OR “peri-implant mucositis” OR “periimplant mucositis” OR “Periimplantitis” OR “peri-implantitis”) AND (“treatment” OR “surgical treatment” OR “resective treatment” OR “surgical therapy” OR “resective therapy” OR “reconstructive therapy” OR “antibiotic treatment” OR “adjunctive treatment” OR “antibiotic therapy” OR “adjunctive therapy” OR “nonsurgical treatment” OR “non-surgical treatment” OR “regenerative treatment” OR “augmentative treatment” OR “reconstructive treatment” OR “regenerative therapy” OR “augmentative therapy”) AND (longitudinal study OR randomised controlled study OR comparative study OR clinical trial) NOT (“photodynamic treatment” OR “photodynamic therapy” OR “antiseptic therapy” OR “antiseptic treatment” OR “local antibiotic*” OR “probiotic” OR systematic review* OR systematic literature OR meta-analysis OR meta-analyses OR in vitro OR animal*) | Dentistry oral surgery medicine | 420 |
| The Cochrane Library (Trials) | (Dent* OR dental OR periodont*) AND (“peri-implant disease” OR “periimplant disease” OR “peri-implant infection” OR “periimplant infection” OR “mucositis” OR “peri-implant mucositis” OR “periimplant mucositis” OR “Periimplantitis” OR “peri-implantitis”) AND (“treatment” OR “surgical treatment” OR “resective treatment” OR “surgical therapy” OR “resective therapy” OR “reconstructive therapy” OR “antibiotic treatment” OR “adjunctive treatment” OR “antibiotic therapy” OR “adjunctive therapy” OR “nonsurgical treatment” OR “non-surgical treatment” OR “regenerative treatment” OR “augmentative treatment” OR “reconstructive treatment” OR “regenerative therapy” OR “augmentative therapy”) AND (longitudinal study OR randomised controlled study OR comparative study OR clinical trial) NOT (“photodynamic treatment” OR “photodynamic therapy” OR “antiseptic therapy” OR “antiseptic treatment” OR “local antibiotic*” OR “probiotic” OR systematic review OR systematic literature OR meta-analysis OR meta-analyses OR in vitro OR animal*) |  | 779 |
| Virtual Health Library (including LILACS, BBO-Dentistry, and CUMED) | (tw:(( dent* OR dental OR periodont* ) AND ( "peri-implant disease" OR "periimplant disease" OR "peri-implant infection" OR "periimplant infection" OR "mucositis" OR "peri-implant mucositis" OR "periimplant mucositis" OR "Periimplantitis" OR "peri-implantitis" ) AND ( "treatment" OR "surgical treatment" OR "resective treatment" OR "surgical therapy" OR "resective therapy" OR "reconstructive therapy" OR "antibiotic treatment" OR "adjunctive treatment" OR "antibiotic therapy" OR "adjunctive therapy" OR "nonsurgical treatment" OR "non-surgical treatment" OR "regenerative treatment" OR "augmentative treatment" OR "reconstructive treatment" OR "regenerative therapy" OR "augmentative therapy" ) not ("photodynamic treatment" OR "photodynamic therapy" OR "antiseptic therapy" OR "antiseptic treatment" OR "local antibiotic*" OR "probiotic" OR systematic AND review* OR systematic AND literature OR meta-analysis OR meta-analyses OR in AND vitro OR animal*))) AND (instance:"regional") AND ( db:("LILACS" OR "IBECS" OR "BBO") AND type_of_study:("case_reports" OR "case_control" OR "clinical_trials" OR "cohort" OR "guideline" OR "overview")) | - | 2 |
| SciELO | (Dent* OR dental OR periodont*) AND (“peri-implant disease” OR “periimplant disease” OR “peri-implant infection” OR “periimplant infection” OR “mucositis” OR “peri-implant mucositis” OR “periimplant mucositis” OR “Periimplantitis” OR “peri-implantitis”) AND (“treatment” OR “surgical treatment” OR “resective treatment” OR “surgical therapy” OR “resective therapy” OR “reconstructive therapy” OR “antibiotic treatment” OR “adjunctive treatment” OR “antibiotic therapy” OR “adjunctive therapy” OR “nonsurgical treatment” OR “non-surgical treatment” OR “regenerative treatment” OR “augmentative treatment” OR “reconstructive treatment” OR “regenerative therapy” OR “augmentative therapy”) | - | 54 |
| **Total** |  |  | 2046 |

**Appendix 3.** Communication attempts with authors of included studies

| **Study** | **Corresponding author** | **Inquiry** | **Status** |
| --- | --- | --- | --- |
| Berglundh 2018 | Tord Berglundh | Asked for additional data and/or dataset | Reply pending |
| Carcuac 2016;2017 | Oliver Carcuac / Jan Derks | Asked for additional data and/or dataset | Sent additional data |
| Carrillo de Albornoz 2024 | Ana Carrillo de Albornoz / Eduardo Montero | Asked for additional data and/or dataset | Sent full dataset |
| Charalampakis 2011 | Georgios Charalampakis | Asked for dataset | Sent full dataset |
| Grundström 2024 | Caroline Riben Grundström | Asked for additional data and/or dataset | Asked for clarifications / additional data; declined provision |
| Hallström 2017 | Gunnar Dahlen | Asked for dataset | Sent full dataset |
| Ramanauskaite 2024 | Ausra Ramanauskaite / Iulia Dahmer | Asked for additional data and/or dataset | Sent additional data |

**Appendix 4.** List of studies identified from the literature searches and inclusion / exclusion status with reasons.

| **Nr** | **Paper** | **Status** |
| --- | --- | --- |
| 1 | . Clinical practice guidelines: Role of bone augmentation for implant placement in the edentulous maxilla. 2016. | Excluded by title |
| 2 | A. A. Al-Hashedi, M. Laurenti, M. N. Abdallah, R. F. Albuquerque and F. Tamimi. Electrochemical Treatment of Contaminated Titanium Surfaces in Vitro: An Approach for Implant Surface Decontamination. 2016. | Excluded by title |
| 3 | A. Aoki, K. Mizutani, F. Schwarz, A. Sculean, R. A. Yukna, A. A. Takasaki, G. E. Romanos, Y. Taniguchi, K. M. Sasaki, J. L. Zeredo, G. Koshy, D. J. Coluzzi, J. M. White, Y. Abiko, I. Ishikawa and Y. Izumi. Periodontal and peri-implant wound healing following laser therapy. 2015. | Excluded by title |
| 4 | A. Azaripour, M. Azaripour, I. Willershausen, C. J. F. Van Noorden and B. Willershausen. Photodynamic Therapy has no Adverse Effects In Vitro on Human Gingival Fibroblasts and Osteoblasts. 2018. | Excluded by title |
| 5 | A. B. Marin-Fernandez, B. Garcia Medina, A. Aguilar-Salvatierra, A. Jimenez-Burkhardt and G. Gomez-Moreno. Jaw osteonecrosis management around a dental implant inserted 2 years before starting treatment with zoledronic acid. 2015. | Excluded by title |
| 6 | A. B. W. Alecio, C. F. Ferreira, J. Babu, T. Shokuhfar, S. Jo, R. Magini and F. Garcia-Godoy. Doxycycline release of dental implants with nanotube surface, coated with poly lactic-co-glycolic acid (PLGA) for extended pH-controlled drug delivery. 2019. | Excluded by title |
| 7 | A. Barone, M. Porzio, L. Ramaglia and L. Sbordone. [Implants in partially edentulous patients with history of periodontal disease]. 2000. | Excluded by title |
| 8 | A. Bedogni, G. Bettini, A. Totola, G. Saia and P. F. Nocini. Oral bisphosphonate-associated osteonecrosis of the jaw after implant surgery: a case report and literature review. 2010. | Excluded by title |
| 9 | A. Boddupalli, L. Zhu and K. M. Bratlie. Methods for Implant Acceptance and Wound Healing: Material Selection and Implant Location Modulate Macrophage and Fibroblast Phenotypes. 2016. | Excluded by title |
| 10 | A. Bowen Antolin, M. T. Pascua Garcia and A. Nasimi. Infections in implantology: from prophylaxis to treatment. 2007. | Excluded by title |
| 11 | A. Bruinink, M. Bitar, M. Pleskova, P. Wick, H. F. Krug and K. Maniura-Weber. Addition of nanoscaledbioinspiredsurface features: A revolution for bone related implants and scaffolds?. 2014. | Excluded by title |
| 12 | A. C. Wetzel, J. Vlassis, R. G. Caffesse, C. H. Hammerle and N. P. Lang. Attempts to obtain re-osseointegration following experimental peri-implantitis in dogs. 1999. | Excluded by title |
| 13 | A. Cordoba, N. Manzanaro-Moreno, C. Colom, H. J. Ronold, S. P. Lyngstadaas, M. Monjo and J. M. Ramis. Quercitrin Nanocoated Implant Surfaces Reduce Osteoclast Activity In Vitro and In Vivo. 2018. | Excluded by title |
| 14 | A. D. Pye, D. E. Lockhart, M. P. Dawson, C. A. Murray and A. J. Smith. A review of dental implants and infection. 2009. | Excluded by title |
| 15 | A. Dashti, D. Ready, V. Salih, J. C. Knowles, J. E. Barralet, M. Wilson, N. Donos and S. N. Nazhat. In vitro antibacterial efficacy of tetracycline hydrochloride adsorbed onto Bio-Oss® bone graft. 2010. | Excluded by title |
| 16 | A. De Ranieri, A. S. Virdi, S. Kuroda, S. Shott, R. M. Leven, N. J. Hallab and D. R. Sumner. Local application of rhTGF-beta2 enhances peri-implant bone volume and bone-implant contact in a rat model. 2005. | Excluded by title |
| 17 | A. E. Parvu, S. Talu, M. A. Taulescu, A. Bota, F. Catoi, C. Craciun, C. Alb, O. Parvu and S. F. Alb. Fractal analysis of ibuprofen effect on experimental dog peri-implantitis. 2014. | Excluded by title |
| 18 | A. E. Steiert, M. Boyce and H. Sorg. Capsular contracture by silicone breast implants: Possible causes, biocompatibility, and prophylactic strategies. 2013. | Excluded by title |
| 19 | A. Eder and G. Watzek. Treatment of a patient with severe osteoporosis and chronic polyarthritis with fixed implant-supported prosthesis: a case report. 1999. | Excluded by title |
| 20 | A. Esberg, C. Isehed, A. Holmlund and P. Lundberg. Peri-implant crevicular fluid proteome before and after adjunctive enamel matrix derivative treatment of peri-implantitis. 2019. | Excluded by title |
| 21 | A. Ewald and S. Ihde. Salt impregnation of implant materials. 2009. | Excluded by title |
| 22 | A. Fernandez Ayora, F. Herion, E. Rompen, J. Y. Reginster, M. Magremanne and F. Lambert. Dramatic osteonecrosis of the jaw associated with oral bisphosphonates, periodontitis, and dental implant removal. 2015. | Excluded by title |
| 23 | A. Ghanem, S. Pasumarthy, V. Ranna, S. V. Kellesarian, T. Abduljabbar, F. Vohra and H. Malmstrom. Is mechanical curettage with adjunct photodynamic therapy more effective in the treatment of peri-implantitis than mechanical curettage alone?. 2016. | Excluded by title |
| 24 | A. H. Almehmadi and F. Alghamdi. Biomarkers of alveolar bone resorption in gingival crevicular fluid: A systematic review. 2018. | Excluded by title |
| 25 | A. Ilea, A. M. Băbţan, B. A. Boşca, M. Crişan, N. B. Petrescu, M. Collino, R. M. Sainz, J. Q. Gerlach and R. S. Câmpian. Advanced glycation end products (AGEs) in oral pathology. 2018. | Excluded by title |
| 26 | A. J. van Winkelhoff and J. W. Wolf. Actinobacillus actinomycetemcomitans-associated peri-implantitis in an edentulous patient. A case report. 2000. | Excluded by title |
| 27 | A. J. van Winkelhoff. [Consensus on peri-implant infections]. 2010. | Excluded by title |
| 28 | A. J. van Winkelhoff. [Dissertations 25 years after date 31. Black-pigmented oral bacteria in human oral infections]. 2012. | Excluded by title |
| 29 | A. J. van Winkelhoff. Antibiotics in the treatment of peri-implantitis. 2012. | Excluded by title |
| 30 | A. Kantarci, H. Hasturk and T. E. Van Dyke. Animal models for periodontal regeneration and peri-implant responses. 2015. | Excluded by title |
| 31 | A. Kashefimehr, R. Pourabbas, M. Faramarzi, A. Zarandi, A. Moradi, H. C. Tenenbaum and A. Azarpazhooh. Effects of enamel matrix derivative on non-surgical management of peri-implant mucositis: a double-blind randomized clinical trial. 2017. | Excluded by title |
| 32 | A. Kelekis-Cholakis and J. Rothney. Maintenance of Implant Patients: A Narrative Review. 2019. | Excluded by title |
| 33 | A. Khojasteh, M. M. Dehghan and P. Nazeman. Immediate implant placement following 1-year treatment with oral versus intravenous bisphosphonates: a histomorphometric canine study on peri-implant bone. 2019. | Excluded by title |
| 34 | A. Kozlovsky, O. Mozes, C. E. Nemcovsky and Z. Artzi. [Differential diagnosis and treatment strategies for peri-implant diseases]. 2003. | Excluded by title |
| 35 | A. L. Neely and A. Maalhagh-Fard. Successful Management of Early Peri-Implant Infection and Bone Loss Using a Multidisciplinary Treatment Approach. 2018. | Excluded by title |
| 36 | A. M. Albaker, A. S. ArRejaie, M. Alrabiah and T. Abduljabbar. Effect of photodynamic and laser therapy in the treatment of peri-implant mucositis: A systematic review. 2018. | Excluded by title |
| 37 | A. M. Decker, R. Sheridan, G. H. Lin, P. Sutthiboonyapan, W. Carroll and H. L. Wang. A Prognosis System for Periimplant Diseases. 2015. | Excluded by title |
| 38 | A. M. Roos-Jansaker, U. S. Almhojd and H. Jansson. Treatment of peri-implantitis: clinical outcome of chloramine as an adjunctive to non-surgical therapy, a randomized clinical trial. 2017. | Excluded by title |
| 39 | A. M. Sultan, Z. E. Hughes and T. R. Walsh. Effect of calcium ions on peptide adsorption at the aqueous rutile titania (110) interface. 2018. | Excluded by title |
| 40 | A. Makeudom, C. Supanchart, P. Montreekachon, S. Khongkhunthian, T. Sastraruji, J. Krisanaprakornkit and S. Krisanaprakornkit. The antimicrobial peptide, human beta-defensin-1, potentiates in vitro osteoclastogenesis via activation of the p44/42 mitogen-activated protein kinases. 2017. | Excluded by title |
| 41 | A. Markovic, A. Dinic, J. L. Calvo Guirado, A. Tahmaseb, M. Scepanovic and B. Janjic. Randomized clinical study of the peri-implant healing to hydrophilic and hydrophobic implant surfaces in patients receiving anticoagulants. 2017. | Excluded by title |
| 42 | A. Materni. Managing an extreme peri-implantitis. 2013. | Excluded by title |
| 43 | A. Mombelli. In vitro models of biological responses to implant microbiological models. 1999. | Excluded by title |
| 44 | A. Mombelli. Maintenance therapy for teeth and implants. 2019. | Excluded by title |
| 45 | A. Mombelli. Microbial colonization of the periodontal pocket and its significance for periodontal therapy. 2018. | Excluded by title |
| 46 | A. Mouratidou, J. Karbach, B. d'Hoedt and B. Al-Nawas. Antibiotic susceptibility of cocultures in polymicrobial infections such as peri-implantitis or periodontitis: an in vitro model. 2011. | Excluded by title |
| 47 | A. N. Fernandes-Costa. Avaliação longitudinal da clorexidina associada ao full-mouth desinfection mucosite peri-implantar. 2015. | Excluded by title |
| 48 | A. N. Idlibi, F. Al-Marrawi, M. Hannig, A. Lehmann, A. Rueppell, A. Schindler, H. Jentsch and S. Rupf. Destruction of oral biofilms formed in situ on machined titanium (Ti) surfaces by cold atmospheric plasma. 2013. | Excluded by title |
| 49 | A. Nakagawa, N. Shitara, Y. Ayukawa, K. Koyano and K. Nishimura. Implant treatment followed by living donor lung transplant: a follow-up case report. 2014. | Excluded by title |
| 50 | Á. O. Salgado-Peralvo, Á. Salgado-García and L. Arriba-Fuente. New tendencies in tissue regeneration: Leucocyte-rich platelet-rich fibrin. 2017. | Excluded by title |
| 51 | A. Ong, J. Kim, S. Loo, A. Quaranta and A. J. Rincon. Prescribing trends of systemic antibiotics by periodontists in Australia. 2019. | Excluded by title |
| 52 | A. Pulcini, J. Bollain, I. Sanz-Sanchez, E. Figuero, B. Alonso, M. Sanz and D. Herrera. Clinical effects of the adjunctive use of a 0.03% chlorhexidine and 0.05% cetylpyridinium chloride mouth rinse in the management of peri-implant diseases: A randomized clinical trial. 2019. | Excluded by title |
| 53 | A. R. Chang, T. H. Cho and S. J. Hwang. Receptor Activator of Nuclear Factor Kappa-B Ligand-Induced Local Osteoporotic Canine Mandible Model for the Evaluation of Peri-Implant Bone Regeneration. 2017. | Excluded by title |
| 54 | A. R. Hohn, C. R. F. Ferreira and R. De Mori. Periimplante: relato de caso clínico. 2001. | Excluded by title |
| 55 | A. R. Sanchez, P. J. Sheridan, S. E. Eckert and A. L. Weaver. Influence of platelet-rich plasma added to xenogeneic bone grafts in periimplant defects: a vital fluorescence study in dogs. 2005. | Excluded by title |
| 56 | A. R. Sanchez, P. J. Sheridan, S. E. Eckert and A. L. Weaver. Regenerative potential of platelet-rich plasma added to xenogenic bone grafts in peri-implant defects: a histomorphometric analysis in dogs. 2005. | Excluded by title |
| 57 | A. R. Sanchez, S. E. Eckert, P. J. Sheridan and A. L. Weaver. Influence of platelet-rich plasma added to xenogeneic bone grafts on bone mineral density associated with dental implants. 2005. | Excluded by title |
| 58 | A. Roffi, B. Di Matteo, G. S. Krishnakumar, E. Kon and G. Filardo. Platelet-rich plasma for the treatment of bone defects: from pre-clinical rational to evidence in the clinical practice. A systematic review. 2017. | Excluded by title |
| 59 | A. Roffi, G. Filardo, E. Kon and M. Marcacci. Does PRP enhance bone integration with grafts, graft substitutes, or implants? A systematic review. 2013. | Excluded by title |
| 60 | A. S. Barakat, M. Alhashash, M. Shousha and H. Boehm. What an orthopaedic surgeon should know about vertebral cement augmentation. 2017. | Excluded by title |
| 61 | A. S. Bidra, M. J. Persenaire and E. Natarajan. Management of peripheral giant cell granuloma around complete-arch fixed implant-supported prosthesis: A case series. 2019. | Excluded by title |
| 62 | A. S. Bidra. No reliable evidence suggesting what is the most effective interventions for treating peri-implantitis. 2012. | Excluded by title |
| 63 | A. S. Bidra. Nonsurgical management of inflammatory periimplant disease caused by food impaction: a clinical report. 2014. | Excluded by title |
| 64 | A. S. Herford, M. Miller and F. Signorino. Maxillofacial Defects and the Use of Growth Factors. 2017. | Excluded by title |
| 65 | A. Scarano, B. Assenza, A. Di Cerbo, V. Candotto, P. S. De Oliveira and F. Lorusso. Bone regeneration in aesthetic areas using titanium micromesh. Three case reports. 2017. | Excluded by title |
| 66 | A. Simonpieri, M. Del Corso, A. Vervelle, R. Jimbo, F. Inchingolo, G. Sammartino and D. M. Dohan Ehrenfest. Current knowledge and perspectives for the use of platelet-rich plasma (PRP) and platelet-rich fibrin (PRF) in oral and maxillofacial surgery part 2: Bone graft, implant and reconstructive surgery. 2012. | Excluded by title |
| 67 | A. Simonpieri, M. Del Corso, G. Sammartino and D. M. Dohan Ehrenfest. The relevance of choukroun's platelet-rich fibrin and metronidazole during complex maxillary rehabilitations using bone allograft. Part I: A new grafting protocol. 2009. | Excluded by title |
| 68 | A. Stavropoulos, K. Bertl, P. Pietschmann, N. Pandis, M. Schiodt and B. Klinge. The effect of antiresorptive drugs on implant therapy: Systematic review and meta-analysis. 2018. | Excluded by title |
| 69 | A. Stavropoulos, L. Kostopoulos, J. R. Nyengaard and T. Karring. Deproteinized bovine bone (Bio-Oss) and bioactive glass (Biogran) arrest bone formation when used as an adjunct to guided tissue regeneration (GTR): an experimental study in the rat. 2003. | Excluded by title |
| 70 | A. Stavropoulos, L. Kostopoulos, N. Mardas, J. R. Nyengaard and T. Karring. Deproteinized bovine bone used as an adjunct to guided bone augmentation: an experimental study in the rat. 2001. | Excluded by title |
| 71 | A. T. Nurden, P. Nurden, M. Sanchez, I. Andia and E. Anitua. Platelets and wound healing. 2008. | Excluded by title |
| 72 | A. Tanner and N. Stillman. Oral and dental infections with anaerobic bacteria: clinical features, predominant pathogens, and treatment. 1993. | Excluded by title |
| 73 | A. Tanner. Microbial etiology of periodontal diseases. Where are we? Where are we going?. 1992. | Excluded by title |
| 74 | A. Tian, J. J. Zhai, Y. Peng, L. Zhang, M. H. Teng, J. Liao, X. Sun and X. Liang. Osteoblast response to titanium surfaces coated with strontium ranelate-loaded chitosan film. 2014. | Excluded by title |
| 75 | A. Vajgel, N. Mardas, B. C. Farias, A. Petrie, R. Cimões and N. Donos. A systematic review on the critical size defect model. 2014. | Excluded by title |
| 76 | A. Vissink, F. K. L. Spijkervet and G. M. Raghoebar. The medically compromised patient: Are dental implants a feasible option?. 2018. | Excluded by title |
| 77 | A. Widodo, D. Spratt, V. Sousa, A. Petrie and N. Donos. An in vitro study on disinfection of titanium surfaces. 2016. | Excluded by title |
| 78 | A. Y. Togashi, R. A. Carmelo and N. C. Pereira. Nível de conhecimento dos cirurgiões-dentistas quanto ao diagnóstico e tratamento da peri-implantite. 2014. | Excluded by title |
| 79 | A. Z. A. Kaissy, A. A. Albir and K. I. A. Mohammed. Quantitative study of the effectiveness of soft laser on the activity of the blood vessels in the spleen of diseased mice with cancer. 2017. | Excluded by title |
| 80 | A. Zampelis, B. Rangert and L. Heijl. Tilting of splinted implants for improved prosthodontic support: A two-dimensional finite element analysis. 2007. | Excluded by title |
| 81 | Abd El Rahim, N. S. and Ashour, A. A.(2022); Assessment of Quality of Life and Supporting Structures in Implant Retained Mandibular Overdenture: A 5-Year Cohort Study. Clinical, Cosmetic and Investigational Dentistry,E3 ,171-182 | Excluded by title |
| 82 | Actrn(2023); Trismus exercise adherence during radiotherapy. https://trialsearch.who.int/Trial2.aspx?TrialID=ACTRN12623001324606,E3 , | Excluded by title |
| 83 | Afrashtehfar, K. I. and Assery, N. M. and Alblooshi, K. A. K. and Schmidlin, P. R.(2024); Maintaining periodontally compromised teeth seems more cost-effective than replacing them with dental implants. Evid Based Dent,E3 , | Excluded by title |
| 84 | Afrashtehfar, K. I. and Hicklin, S. P. and Schmidlin, P. R.(2024); LONG-TERM OUTCOMES OF IMPLANT PLACEMENT VERSUS TOOTH PRESERVATION IN PERIODONTALLY COMPROMISED TEETH MAY BE COMPARABLE. Journal of Evidence-Based Dental Practice,E3 ,14 | Excluded by title |
| 85 | Agurto, M. G. and Carpenter, G. H. and Bozorgi, S. S. and Koller, G. and Fenlon, M. and Warburton, F. and Bruce, K. and Burke, M. and Banerjee, A.(2024); Analysis of the association between salivary proteins and oral mucositis in patients with head and neck cancer undergoing IMRT: a longitudinal study. Bmc Oral Health,E3 ,112 | Excluded by title |
| 86 | Alarc√≥n, M. A. and Esparza, D. and Montoya, C. and Monje, A. and Faggion, C. M.(2017); The 300 most-cited articles in implant dentistry. International Journal of Oral and Maxillofacial Implants,E3 ,1e1-e8 | Excluded by title |
| 87 | AlHelal, A. A. and Alzaid, A. A. and Almujel, S. H. and Alsaloum, M. and Alanazi, K. K. and Althubaitiy, R. O. and Al-Aali, K. A.(2024); Evaluation of Peri-Implant Parameters and Functional Outcome of Immediately Placed and Loaded Mandibular Overdentures: A 5-year Follow-up Study. Oral Health & Preventive Dentistry,E3 ,123-30 | Excluded by title |
| 88 | Alpaslan, N. Z. and Altindal, D. and Akbal, D. and Talmac, A. C. and Tunc, S. K. and Ertugrul, A. S.(2024); Evaluation of the effect of Er,Cr:YSGG laser application on peri-implant crevicular fluid receptor activator of nuclear factor-kappa B ligand and osteoprotegerin levels in the non-surgical treatment of peri-implantitis: A randomized clinical trial. Journal of Periodontology,E3 ,12 | Excluded by title |
| 89 | Ambili, R. and Ramadas, K. and Nair, L. M. and Raj, D. and Nazeer, F. and George, P. S. and Kumar, R. R. and Pillai, M. R.(2023); Efficacy of a herbal mouthwash for management of periodontitis and radiation-induced mucositis e A consolidated report of two randomized controlled clinical trials. Journal of Ayurveda and Integrative Medicine,E3 ,68 | Excluded by title |
| 90 | Ambili, R. and Ramadas, K. and Nair, L. M. and Raj, D. and Nazeer, F. and George, P. S. and Rejnish Kumar, R. and Radhakrishna Pillai, M.(2023); Efficacy of a herbal mouthwash for management of periodontitis and radiation-induced mucositis - A consolidated report of two randomized controlled clinical trials. Journal of Ayurveda and integrative medicine,E3 ,6100791 | Excluded by title |
| 91 | Ambili, R. and Ramadas, K. and Nair, L. M. and Raj, D. and Nazeer, F. and George, P. S. and Rejnish Kumar, R. and Radhakrishna Pillai, M.(2023); Efficacy of a herbal mouthwash for management of periodontitis and radiation-induced mucositis ‚Äì A consolidated report of two randomized controlled clinical trials. Journal of Ayurveda and integrative medicine,E3 ,6 | Excluded by title |
| 92 | Ardakani, M. T. and Amid, R. and Moscowchi, A.(2023); EXPLANTATION SITE MANAGEMENT: A DECISION ALGORITHM FOR REIMPLANTATION. International Journal of Periodontics & Restorative Dentistry,E3 ,S26-S35 | Excluded by title |
| 93 | Attia, S. and Narberhaus, C. and Schaaf, H. and Streckbein, P. and Pons-k√ºhnemann, J. and Schmitt, C. and Neukam, F. W. and Howaldt, H. P. and B√∂ttger, S.(2020); Long-term influence of platelet-rich plasma (Prp) on dental implants after maxillary augmentation: Retrospective clinical and radiological outcomes of a randomized controlled clinical trial. Journal of Clinical Medicine,E3 ,2 | Excluded by title |
| 94 | Attik, N. and Phantarasmy, M. and Abouelleil, H. and Chevalier, C. and Barraco, A. and Grosgogeat, B. and Lafon, A.(2022); Comparison of the Biological Behavior and Topographical Surface Assessment of a Minimally Invasive Dental Implant and a Standard Implant: An In Vitro Study. Materials,E3 ,21 | Excluded by title |
| 95 | B. Al-Nawas and J. Karbach. Antibiotics in implant dentistry. 2012. | Excluded by title |
| 96 | B. B. Seo, H. I. Chang, H. Choi, J. T. Koh, K. D. Yun, J. Y. Lee and S. C. Song. New approach for vertical bone regeneration using in situ gelling and sustained BMP-2 releasing poly(phosphazene) hydrogel system on peri-implant site with critical defect in a canine model. 2018. | Excluded by title |
| 97 | B. Burzyńska and E. Mierzwińska-Nastalska. Implant treatment of patients with diabetes mellitus. 2013. | Excluded by title |
| 98 | B. G. X. Zhang, D. E. Myers, G. G. Wallace, M. Brandt and P. F. M. Choong. Bioactive coatings for orthopaedic implants-recent trends in development of implant coatings. 2014. | Excluded by title |
| 99 | B. Garcia Garcia, A. Dean Ferrer, N. Diaz Jimenez and F. J. Alamillos Granados. Osteonecrosis de los maxilares asociada a denosumab en una paciente con osteoporosis: un caso clínico. 2015. | Excluded by title |
| 100 | B. Klinge, A. Gustafsson and T. Berglundh. A systematic review of the effect of anti-infective therapy in the treatment of peri-implantitis. 2002. | Excluded by title |
| 101 | B. Klinge, T. Flemming, J. Cosyn, H. De Bruyn, B. M. Eisner, M. Hultin, F. Isidor, N. P. Lang, B. Lund, J. Meyle, A. Mombelli, J. M. Navarro, B. Pjetursson, S. Renvert and H. Schliephake. The patient undergoing implant therapy. Summary and consensus statements. The 4th EAO Consensus Conference 2015. 2015. | Excluded by title |
| 102 | B. Lethaus and J. Zoller. Systemic factors in dental implantology. 2016. | Excluded by title |
| 103 | B. M. Holzapfel, J. C. Reichert, J. T. Schantz, U. Gbureck, L. Rackwitz, U. Nöth, F. Jakob, M. Rudert, J. Groll and D. W. Hutmacher. How smart do biomaterials need to be? A translational science and clinical point of view. 2013. | Excluded by title |
| 104 | B. Mair, S. Tangl, J. Feierfeil, D. Skiba, G. Watzek and R. Gruber. Age-related efficacy of parathyroid hormone on osseointegration in the rat. 2009. | Excluded by title |
| 105 | B. R. Chrcanovic, M. D. Martins and A. Wennerberg. Immediate Placement of Implants into Infected Sites: A Systematic Review. 2015. | Excluded by title |
| 106 | B. Reinhardt, A. Klocke, S. H. Neering, S. Selbach, U. Peters, T. F. Flemmig and T. Beikler. Microbiological dynamics of red complex bacteria following full-mouth air polishing in periodontally healthy subjects-a randomized clinical pilot study. 2019. | Excluded by title |
| 107 | B. S. Lee, K. S. Shih, C. H. Lai, Y. Takeuchi and Y. W. Chen. Surface property alterations and osteoblast attachment to contaminated titanium surfaces after different surface treatments: An in vitro study. 2018. | Excluded by title |
| 108 | B. Shrestha, M. L. Theerathavaj, S. Thaweboon and B. Thaweboon. In vitro antimicrobial effects of grape seed extract on peri-implantitis microflora in craniofacial implants. 2012. | Excluded by title |
| 109 | B. Stavaru Marinescu, L. Naval Gías and G. Herrera Calvo. Periimplantitis apical - presentación de serie de 11 casos clínicos. 2015. | Excluded by title |
| 110 | B. T. Goh, N. Chanchareonsook, H. Tideman, S. H. Teoh, J. K. Chow and J. A. Jansen. The use of a polycaprolactone-tricalcium phosphate scaffold for bone regeneration of tooth socket facial wall defects and simultaneous immediate dental implant placement in Macaca fascicularis. 2014. | Excluded by title |
| 111 | B. T. Le and A. Borzabadi-Farahani. Simultaneous implant placement and bone grafting with particulate mineralized allograft in sites with buccal wall defects, a three-year follow-up and review of literature. 2014. | Excluded by title |
| 112 | B. W. Sigusch, M. Engelbrecht, A. Volpel, A. Holletschke, W. Pfister and J. Schutze. Full-Mouth Antimicrobial Photodynamic Therapy in Fusobacterium nucleatum-Infected Periodontitis Patients. 2010. | Excluded by title |
| 113 | B. Zhu, S. Gong and W. Cheng. Softening gold for elastronics. 2019. | Excluded by title |
| 114 | Bassetti, M. and Sch√§r, D. and Wicki, B. and Eick, S. and Ramseier, C. A. and Arweiler, N. B. and Sculean, A. and Salvi, G. E.(2014); Anti-infective therapy of peri-implantitis with adjunctive local drug delivery or photodynamic therapy: 12-month outcomes of a randomized controlled clinical trial. Clinical Oral Implants Research,E3 ,3279-287 | Excluded by title |
| 115 | Bazzani, D. and Heidrich, V. and Manghi, P. and Blanco-Miguez, A. and Asnicar, F. and Armanini, F. and Cavaliere, S. and Bertelle, A. and Dell'Acqua, F. and Dellasega, E. and Waldner, R. and Vicentini, D. and Bolzan, M. and Tomasi, C. and Segata, N. and Pasolli, E. and Ghensi, P.(2024); Favorable subgingival plaque microbiome shifts are associated with clinical treatment for peri-implant diseases. Npj Biofilms and Microbiomes,E3 ,16 | Excluded by title |
| 116 | Becker, K. and Brunello, G. and Scotti, L. and Drescher, D. and John, G.(2021); Efficacy of 0.05% chlorhexidine and 0.05% cetylpyridinium chloride mouthwash to eliminate living bacteria on in situ collected biofilms: An in vitro study. Antibiotics,E3 ,6 | Excluded by title |
| 117 | Beikler, T. and Sch√§fer, I. and Flemmig, T. F.(2005); Infectious peri-implant complications - diagnosis, prevention, and therapy. Implantologie,E3 ,3211-222 | Excluded by title |
| 118 | Berglundh, T. and Mombelli, A. and Schwarz, F. and Derks, J.(2024); Etiology, pathogenesis and treatment of peri-implantitis: A European perspective. Periodontology 2000,E3 ,36 | Excluded by title |
| 119 | Bernardi, S. and Gerardi, D. and Bartsch, S. and Macchiarelli, G. and Hellwig, E. and Al-Ahmad, A.(2024); Antimicrobial therapy using VIS plus water-filtered infrared-A as an alternative method to treat oral diseases. Future Microbiology,E3 ,3241-254 | Excluded by title |
| 120 | Berton, F. and Zotti, M. and Rapani, A. and Costantinides, F. and Castronovo, G. and Di Lenarda, R. and Stacchi, C.(2024); Implant Survival in Renal-Transplanted Patients: A Prospective Long-Term Study. J Oral Implantol,E3 ,267-73 | Excluded by title |
| 121 | Blanco-Ruiz, S. and Molinero-Mourelle, P. and Blanco-Ruiz, M. and Fern√°ndez-Tresguerres, F. G. and Blanco-Samper, S. and L√≥pez-Quiles, J.(2023); Effect of the buccal fat pad in the prevention of zygomatic implant surgery postoperative complications: a pilot study. Medicina oral, patologia oral y cirugia bucal,E3 ,4e371‚Äêe377 | Excluded by title |
| 122 | Bonomi, M. and Blakaj, D. M. and Kabarriti, R. and Colvett, K. and Takiar, V. and Biagioli, M. and Bar -Ad, V. and Goyal, S. and Muzyka, B. and Niermann, K. and Abrouk, N. and Oronsky, B. and Reid, T. and Caroen, S. and Sonis, S. and Sher, D. J.(2023); PREVLAR: Phase 2a Randomized Trial to Assess the Safety and Efficacy of RRx-001 in the Attenuation of Oral Mucositis in Patients Receiving Head and Neck Chemoradiotherapy. International Journal of Radiation Oncology Biology Physics,E3 ,3551-559 | Excluded by title |
| 123 | bqywp, R. B. R.(2024); Use of Simvastatin Gel for the treatment of Peri-implantitis. https://trialsearch.who.int/Trial2.aspx?TrialID=RBR-3bqywp7,E3 , | Excluded by title |
| 124 | Bu, X. S. and Wang, X. and Pan, T. H. and Zhu, L. L. and Guo, J. C.(2024); Clinical effect of MB-PDT assisted initial therapy on alveolar angle absorption of lower anterior teeth. Shanghai kou qiang yi xue [Shanghai journal of stomatology],E3 ,3265‚Äê268 | Excluded by title |
| 125 | Bunoiu, I. and Andrei, M. and Scheau, C. and Manole, C. C. and Stoian, A. B. and Cioranu, V. S. I. and Didilescu, A. C.(2020); Electrochemical behavior of rejected dental implants in peri-implantitis. Coatings,E3 ,3 | Excluded by title |
| 126 | Butera, A. and Pascadopoli, M. and Pellegrini, M. and Gallo, S. and Zampetti, P. and Cuggia, G. and Scribante, A.(2022); Domiciliary Use of Chlorhexidine vs. Postbiotic Gels in Patients with Peri-Implant Mucositis: A Split-Mouth Randomized Clinical Trial. Applied Sciences (Switzerland),E3 ,6 | Excluded by title |
| 127 | C. A. Acevedo, Y. Olguín, M. Briceño, J. C. Forero, N. Osses, P. Díaz-Calderón, A. Jaques and R. Ortiz. Design of a biodegradable UV-irradiated gelatin-chitosan/nanocomposed membrane with osteogenic ability for application in bone regeneration. 2019. | Excluded by title |
| 128 | C. A. Landes, S. Ghanaati, A. Ballon, V. D. Thai, R. Reinhard, D. Nolte, A. Piwowarczyk, M. Wagner and R. Sader. Severely scarred oronasal cleft defects in edentulous adults: Initial data on the long-term outcome of telescoped obturator prostheses supported by zygomatic implants. 2013. | Excluded by title |
| 129 | C. Constantinides, J. Chang and P. Fletcher. Management of an Ailing Anterior Implant Using a Minimally Invasive Flapless Surgical Technique: A Case Report. 2017. | Excluded by title |
| 130 | C. Doll, S. Hartwig, C. Nack, S. Nahles, K. Nelson and J. D. Raguse. Dramatic course of osteomyelitis in a patient treated with immediately placed dental implants suffering from uncontrolled diabetes: A case report. 2015. | Excluded by title |
| 131 | C. F. Goncalves, A. C. Desidera, G. C. do Nascimento, J. P. Issa and C. R. Leite-Panissi. Experimental tooth movement and photobiomodulation on bone remodeling in rats. 2016. | Excluded by title |
| 132 | C. G. Dodo, L. Meirelles, A. Aviles-Reyes, K. G. S. Ruiz, J. Abranches and A. Cury. Pro-inflammatory Analysis of Macrophages in Contact with Titanium Particles and Porphyromonas gingivalis. 2017. | Excluded by title |
| 133 | C. Galli, G. Pedrazzi, M. Mattioli-Belmonte and S. Guizzardi. The Use of Pulsed Electromagnetic Fields to Promote Bone Responses to Biomaterials in Vitro and in Vivo. 2018. | Excluded by title |
| 134 | C. Gao, C. Li, C. Wang, Y. Qin, Z. Wang, F. Yang, H. Liu, F. Chang and J. Wang. Advances in the induction of osteogenesis by zinc surface modification based on titanium alloy substrates for medical implants. 2017. | Excluded by title |
| 135 | C. H. F. Hammerle and D. Tarnow. The etiology of hard- and soft-tissue deficiencies at dental implants: A narrative review. 2018. | Excluded by title |
| 136 | C. H. Fang, P. I. Tsai, S. W. Huang, J. S. Sun, J. Z. Chang, H. H. Shen, S. Y. Chen, F. H. Lin, L. T. Hsu and Y. C. Chen. Magnetic hyperthermia enhance the treatment efficacy of peri-implant osteomyelitis. 2017. | Excluded by title |
| 137 | C. H. Hammerle, I. Fourmousis, J. R. Winkler, C. Weigel, U. Bragger and N. P. Lang. Successful bone fill in late peri-implant defects using guided tissue regeneration. A short communication. 1995. | Excluded by title |
| 138 | C. J. Chen, C. C. Chen and S. J. Ding. Effectiveness of Hypochlorous Acid to Reduce the Biofilms on Titanium Alloy Surfaces in Vitro. 2016. | Excluded by title |
| 139 | C. J. Chen, S. J. Ding and C. C. Chen. Effects of Surface Conditions of Titanium Dental Implants on Bacterial Adhesion. 2016. | Excluded by title |
| 140 | C. K. Kim, H. Y. Kim, J. K. Chai, K. S. Cho, I. S. Moon, S. H. Choi, J. S. Sottosanti and U. M. E. Wikesjö. Effect of a Calcium Sulfate Implant with Calcium Sulfate Barrier on Periodontal Healing in 3-Wall Intrabony Defects in Dogs. 1998. | Excluded by title |
| 141 | C. M. Ardila Medina and I. C. Guzmán Zuluaga. Eficacia de las alternativas de tratamiento para la mucositis periimplantaria. 2014. | Excluded by title |
| 142 | C. M. Faggion, Jr. and M. Schmitter. Using the best available evidence to support clinical decisions in implant dentistry. 2010. | Excluded by title |
| 143 | C. M. Faggion, Jr., S. Listl, N. Fruhauf, H. J. Chang and Y. K. Tu. A systematic review and Bayesian network meta-analysis of randomized clinical trials on non-surgical treatments for peri-implantitis. 2014. | Excluded by title |
| 144 | C. M. Reddy, A. W. Brock, B. G. Coleman, K. J. Erley and T. M. Johnson. Should Perioperative Antibiotics Be Prescribed Prophylactically for Uncomplicated Single Implant Surgeries?. 2017. | Excluded by title |
| 145 | C. Mas-Moruno, B. Su and M. J. Dalby. Multifunctional Coatings and Nanotopographies: Toward Cell Instructive and Antibacterial Implants. 2019. | Excluded by title |
| 146 | C. Mongardini, A. Pilloni, R. Farina, G. Di Tanna and B. Zeza. Adjunctive efficacy of probiotics in the treatment of experimental peri-implant mucositis with mechanical and photodynamic therapy: a randomized, cross-over clinical trial. 2017. | Excluded by title |
| 147 | C. N. Yukna and R. A. Yukna. Multi-center evaluation of bioabsorbable collagen membrane for guided tissue regeneration in human class II furcations. 1996. | Excluded by title |
| 148 | C. Passariello, A. Lucchese, F. Pera and P. Gigola. CLINICAL, MICROBIOLOGICAL AND INFLAMMATORY EVIDENCE OF THE EFFICACY OF COMBINATION THERAPY INCLUDING SERRATIOPEPTIDASE IN THE TREATMENT OF PERIIMPLANTITIS. 2012. | Excluded by title |
| 149 | C. S. Tastepe, X. Lin, M. Donnet, B. Z. Doulabi, D. Wismeijer and Y. Liu. Re-establishment of Biocompatibility of the In Vitro Contaminated Titanium Surface Using Osteoconductive Powders With Air-Abrasive Treatment. 2018. | Excluded by title |
| 150 | C. S. Tastepe, Y. Liu, C. M. Visscher and D. Wismeijer. Cleaning and modification of intraorally contaminated titanium discs with calcium phosphate powder abrasive treatment. 2013. | Excluded by title |
| 151 | C. Schouten, G. J. Meijer, J. J. J. P. Van Den Beucken, P. H. M. Spauwen and J. A. Jansen. Effects of implant geometry, surface properties, and TGF-β1 on peri-implant bone response: An experimental study in goats. 2009. | Excluded by title |
| 152 | C. Schouten, G. J. Meijer, J. J. J. P. van den Beucken, P. H. M. Spauwen and J. A. Jansen. The quantitative assessment of peri-implant bone responses using histomorphometry and micro-computed tomography. 2009. | Excluded by title |
| 153 | C. Susin, T. Fiorini, J. Lee, J. A. De Stefano, D. P. Dickinson and U. M. E. Wikesjö. Wound healing following surgical and regenerative periodontal therapy. 2015. | Excluded by title |
| 154 | C. Vaquette, S. P. Pilipchuk, P. M. Bartold, D. W. Hutmacher, W. V. Giannobile and S. Ivanovski. Tissue Engineered Constructs for Periodontal Regeneration: Current Status and Future Perspectives. 2018. | Excluded by title |
| 155 | C. von Wilmowsky, P. Stockmann, I. Harsch, K. Amann, P. Metzler, R. Lutz, T. Moest, F. W. Neukam and K. A. Schlegel. Diabetes mellitus negatively affects peri-implant bone formation in the diabetic domestic pig. 2011. | Excluded by title |
| 156 | C. X. Sun, J. M. Henkin, C. Ririe and E. Javadi. Implant failure associated with actinomycosis in a medically compromised patient. 2013. | Excluded by title |
| 157 | Cabanes-Gumbau, G. and Padulles-Roig, E. and Kois, J. C. and Revilla-Le√≥n, M.(2024); Implant-supported prostheses following the biologically oriented preparation technique (BOPT) after implantoplasty procedures: A dental technique. Journal of Prosthetic Dentistry,E3 , | Excluded by title |
| 158 | Cappar√®, P. and Tet√®, G. and D‚ÄôOrto, B. and Nagni, M. and Gherlone, E. F.(2023); Immediate Loaded Full-Arch Mandibular Rehabilitations in Younger vs. Elderly Patients: A Comparative Retrospective Study with 7-Year Follow-Up. Journal of Clinical Medicine,E3 ,13 | Excluded by title |
| 159 | ch2tf, R. B. R.(2023); The effect of using two mouthwashes in the treatment of mucosal inflammation around dental implants. https://trialsearch.who.int/Trial2.aspx?TrialID=RBR-10ch2tf2,E3 , | Excluded by title |
| 160 | Chang, S. H. and Mei, H. I. and Lin, C. L.(2020); Integrating CAD and 3D-printing techniques to construct an in vitro laser standard treatment platform for evaluating the effectiveness of sterilization by Er: YAG laser in peri-implant intra-bony defects. Applied Sciences (Switzerland),E3 ,10 | Excluded by title |
| 161 | ChiCtr(2023); Clinical study on re-mineralization of nano-hydroxyapatite toothpaste. https://trialsearch.who.int/Trial2.aspx?TrialID=ChiCTR2300070488,E3 , | Excluded by title |
| 162 | ChiCtr(2023); Clinical study on the application of Hydraulic Crestal Sinus Augmentation combined with aPRF in patients with severe deficiency of maxillary posterior sinus crest. https://trialsearch.who.int/Trial2.aspx?TrialID=ChiCTR2300068012,E3 , | Excluded by title |
| 163 | ChiCtr(2024); Improvement of self-management ability in elderly patients with periodontitis after implantation based on Com-B theory: a randomized controlled study. https://trialsearch.who.int/Trial2.aspx?TrialID=ChiCTR2400082660,E3 , | Excluded by title |
| 164 | Chin, H. L. and Chung, K. P. and Liu, H. C. and Chen, R. S. and Chang, H. H. and Chen, M. H.(2023); Efficacy of polyacrylate silver salt/polyvinylpyrrolidone-based liquid oral gel in management of concurrence chemoradiotherapy-induced oral mucositis. Journal of the Formosan Medical Association,E3 ,8723-730 | Excluded by title |
| 165 | Chitsazi, M. T. and Khorramdel, A. and Mesforoush, M. A.(2022); Effect of sterile tetracycline ophthalmic ointment as an adjuvant to mechanical debridement on the treatment of peri-implantitis: A randomized controlled clinical trial. Journal of Advanced Periodontology and Implant Dentistry,E3 ,126-31 | Excluded by title |
| 166 | Cho, A. R. and Son, H. and Han, G.(2024); Effect of Honey-based Oral Care on Oral Health of Patients With Stroke Undergoing Rehabilitation: A Randomized Controlled Trial. Asian Nursing Research,E3 ,3215-221 | Excluded by title |
| 167 | Cobo-V√°zquez, C. M. and Molinero-Mourelle, P. and Romeo-Rubio, M. and Guisado-Moya, B. F. and Del R√≠o-Highsmith, J. and L√≥pez-Quiles, J.(2024); Prospective clinical-radiological study of the survival and behavior of short implants. Journal of Prosthetic Dentistry,E3 , | Excluded by title |
| 168 | Colella, G. and Boschetti, C. E. and Vitagliano, R. and Colella, C. and Jiao, L. B. and King-Smith, N. and Li, C. and Lau, Y. N. and Lai, Z. C. and Mohammed, A. I. and Cirillo, N.(2023); Interventions for the Prevention of Oral Mucositis in Patients Receiving Cancer Treatment: Evidence from Randomised Controlled Trials. Current Oncology,E3 ,1967-980 | Excluded by title |
| 169 | Correia, F. and Gouveia, S. and Felino, A. C. and Faria-Almeida, R. and Pozza, D. H.(2024); Maxillary Sinus Augmentation with Xenogenic Collagen-Retained Heterologous Cortico-Cancellous Bone: A 3-Year Follow-Up Randomized Controlled Trial. Dentistry Journal,E3 ,213 | Excluded by title |
| 170 | Corsalini, M. and Montagnani, M. and Charitos, I. A. and Bottalico, L. and Barile, G. and Santacroce, L.(2023); Non-Surgical Therapy and Oral Microbiota Features in Peri-Implant Complications: A Brief Narrative Review. Healthcare,E3 ,516 | Excluded by title |
| 171 | Ctri(2023); Compare and evaluate the effect of cryotherapy and laser therapy in single rooted teeth after single sitting Root Canal Treatment(RCT). https://trialsearch.who.int/Trial2.aspx?TrialID=CTRI/2023/07/055237,E3 , | Excluded by title |
| 172 | Ctri(2023); Comparison of Peri-Implant Clinical and Radiological Parameters after using Glycine Air Abrasion, Soft Tissue Diode Laser or Titanium Brush adjunctive to Conventional Peri-Implantitis Therapy. https://trialsearch.who.int/Trial2.aspx?TrialID=CTRI/2023/05/052252,E3 , | Excluded by title |
| 173 | Ctri(2023); LASER Assisted New Attachment Procedure, Excisional New Attachment Procedure and Subgingival curettage in the treatment of chronic periodontitis. https://trialsearch.who.int/Trial2.aspx?TrialID=CTRI/2023/09/057472,E3 , | Excluded by title |
| 174 | Ctri(2023); The effect of submucosal injection of platelet rich plasma and perforations in alveolar bone for reducing the time required for correction of cramped lower jaw front teeth using fixed orthodontic appliance. https://trialsearch.who.int/Trial2.aspx?TrialID=CTRI/2023/09/057246,E3 , | Excluded by title |
| 175 | Ctri(2024); A study to compare the removal of dead tooth and placing it back after removing the infection with gum surgery for treating root infection in teeth. https://trialsearch.who.int/Trial2.aspx?TrialID=CTRI/2024/01/061132,E3 , | Excluded by title |
| 176 | Ctri(2024); Efficacy of rose Bengal dye in addition with mechanical cleaning using laser assisted therapy in implant defects. https://trialsearch.who.int/Trial2.aspx?TrialID=CTRI/2024/04/065478,E3 , | Excluded by title |
| 177 | Ctri(2024); Intranasal sedation and Nitrous oxide-Oxygen inhalation sedation for 5-10 year old Anxious Children Undergoing Dental Treatment. https://trialsearch.who.int/Trial2.aspx?TrialID=CTRI/2024/01/061815,E3 , | Excluded by title |
| 178 | D. A. Di Stefano, G. B. Greco, L. Cinci and L. Pieri. Horizontal-guided bone regeneration using a titanium mesh and an equine bone graft. 2015. | Excluded by title |
| 179 | D. B. Spagnoli, R. Mazzonetto and J. M. Marchena. Clinical procedures currently using bone grafting with guided tissue regeneration techniques. 2001. | Excluded by title |
| 180 | D. C. Matthews. Prevention and treatment of periodontal diseases in primary care. 2014. | Excluded by title |
| 181 | D. Cena, E. Biljana and P. Mirjana. The use of graft materials and platelet rich plasma in oral surgery. 2017. | Excluded by title |
| 182 | D. D. Dean, C. M. Campbell, S. F. Gruwell, J. W. Tindall, H. H. Chuang, W. Zhong, J. P. Schmitz and V. L. Sylvia. Arachidonic acid and prostaglandin E2 influence human osteoblast (MG63) response to titanium surface roughness. 2008. | Excluded by title |
| 183 | D. F. Williams. Biocompatibility pathways: Biomaterials-induced sterile inflammation, mechanotransduction, and principles of biocompatibility control. 2017. | Excluded by title |
| 184 | D. Flanagan. Implant Placement in Failed Endodontic Sites: A Review. 2016. | Excluded by title |
| 185 | D. Gothard, E. L. Smith, J. M. Kanczler, H. Rashidi, O. Qutachi, J. Henstock, M. Rotherham, A. El Haj, K. M. Shakesheff and R. O. C. Oreffo. Tissue engineered bone using select growth factors: A comprehensive review of animal studies and clinical translation studies in man. 2014. | Excluded by title |
| 186 | D. L. Wang, W. X. Xu, N. Lin, H. Y. Wang, Y. Shi, X. G. Yu, Q. L. Li, Y. Zhou and H. R. Luan. Platelet-rich fibrin combined with tooth ash promotes bone repair. 2018. | Excluded by title |
| 187 | D. Lebeaux, A. Chauhan, O. Rendueles and C. Beloin. From in vitro to in vivo models of bacterial biofilm-related infections. 2013. | Excluded by title |
| 188 | D. Losic, M. S. Aw, A. Santos, K. Gulati and M. Bariana. Titania nanotube arrays for local drug delivery: Recent advances and perspectives. 2015. | Excluded by title |
| 189 | D. Morales Navarro and D. Vila Morales. Guided bone regeneration in dental practice. 2016. | Excluded by title |
| 190 | D. Nikolidakis, G. J. Meijer, D. A. W. Oortgiesen, X. F. Walboomers and J. A. Jansen. The effect of a low dose of transforming growth factor β1 (TGF-β1) on the early bone-healing around oral implants inserted in trabecular bone. 2009. | Excluded by title |
| 191 | D. Oliveira, J. S. Hassumi, P. H. Gomes-Ferreira, T. O. Polo, G. R. Ferreira, L. P. Faverani and R. Okamoto. Short term sodium alendronate administration improves the peri-implant bone quality in osteoporotic animals. 2017. | Excluded by title |
| 192 | D. P. Dowling, S. Maher, V. J. Law, M. Ardhaoui, C. Stallard and A. Keenan. Modified drug release using atmospheric pressure plasma deposited siloxane coatings. 2016. | Excluded by title |
| 193 | D. Peñarrocha-Oltra, A. Aloy-Prósper, J. Cervera-Ballester, M. Peñarrocha-Diago, L. Canullo and M. Peñarrocha-Diago. Implant treatment in atrophic posterior mandibles: Vertical regeneration with block bone grafts versus implants with 5.5-mm intrabony length. 2014. | Excluded by title |
| 194 | D. R. Bijukumar, C. McGeehan and M. T. Mathew. Regenerative Medicine Strategies in Biomedical Implants. 2018. | Excluded by title |
| 195 | D. R. Haynes. Bone lysis and inflammation. 2004. | Excluded by title |
| 196 | D. S. Thoma, I. S. Martin, S. Mühlemann and R. E. Jung. Systematic review of pre-clinical models assessing implant integration in locally compromised sites and/or systemically compromised animals. 2012. | Excluded by title |
| 197 | D. S. Thoma, U. W. Jung, J. Y. Park, S. P. Bienz, J. Husler and R. E. Jung. Bone augmentation at peri-implant dehiscence defects comparing a synthetic polyethylene glycol hydrogel matrix vs. standard guided bone regeneration techniques. 2017. | Excluded by title |
| 198 | D. Schar, C. A. Ramseier, S. Eick, N. B. Arweiler, A. Sculean and G. E. Salvi. Anti-infective therapy of peri-implantitis with adjunctive local drug delivery or photodynamic therapy: six-month outcomes of a prospective randomized clinical trial. 2013. | Excluded by title |
| 199 | D. Sebring, T. Kvist and J. Derks. Indications for Extraction before Implant Therapy: Focus on Endodontic Status. 2019. | Excluded by title |
| 200 | D. W. Paquette, M. E. Ryan and R. S. Wilder. Locally delivered antimicrobials: clinical evidence and relevance. 2008. | Excluded by title |
| 201 | D. Xue and Y. Zhao. Clinical effectiveness of adjunctive antimicrobial photodynamic therapy for residual pockets during supportive periodontal therapy: A systematic review and meta-analysis. 2017. | Excluded by title |
| 202 | D. Ye and A. Peramo. Implementing tissue engineering and regenerative medicine solutions in medical implants. 2014. | Excluded by title |
| 203 | de Chirurgie Orale, S. F.(2012); Prise en charge des foyers infectieux bucco-dentaires. Medecine Buccale Chirurgie Buccale,E3 ,3251-314 | Excluded by title |
| 204 | de Oliveira, F. M. A. and Borges, M. M. F. and Malta, C. E. N. and de Moura, J. F. B. and Forte, C. P. F. and Barbosa, J. V. and Silva, P. G. D. and Dantas, T. S.(2024); Comparison of a daily and alternate-day photobiomodulation protocol in the prevention of oral mucositis in patients undergoing radiochemotherapy for oral cancer: a triple-blind, controlled clinical trial. Medicina Oral Patologia Oral Y Cirugia Bucal,E3 ,3e430-e440 | Excluded by title |
| 205 | Deb, B. J. and Santhanam, V. and Narayanamurthy, U. and Raizada, P. and Jaine, R. and Murugesan, S.(2023); Evaluation of Efficacy and Safety of Zinobliss (Zinc Carnosine 2% and Lignocaine 2%) for the Management of Oral Mucositis and Stomatitis: a Phase 3 Prospective Randomized Open Label Multi Centric Parallel Design Study. International journal of scientific research in dental and medical sciences,E3 ,4181‚Äê187 | Excluded by title |
| 206 | Deepa, K. Mujawar, K. Dhillon, P. Jadhav, I. Das and Y. K. Singla. Prognostic Implication of Selective Serotonin Reuptake Inhibitors in Osseointegration of Dental Implants: A 5-year Retrospective Study. 2018. | Excluded by title |
| 207 | Deppe, H. and L√ºth, T. and Wolff, K. D.(2015); Laser application in the treatment of peri-implantitis: 2015 update. Implantologie,E3 ,3287-294 | Excluded by title |
| 208 | Dewan, H. and Mansuri, S. and Bansal, K. and Basutkar, N. and Saini, M. and Nayan, K.(2023); Comparison of the quality of life among the immediate loading implant overdentures using the single and the double implants: an original study. Journal of pharmacy & bioallied sciences,E3 ,5161‚Äê165 | Excluded by title |
| 209 | Deya, J. and Delannoy, S. and Vermaut, P. and Prima, F.(2024); Thermomechanical Pathways for Accurate Microstructural Control of Ti‚Äì7Ag Alloy: Towards a New Generation of Antibacterial Materials for Medical Applications. Metals,E3 ,5 | Excluded by title |
| 210 | Dhingra, K. and Dinda, A. K. and Kottarath, S. K. and Chaudhari, P. K. and Verma, F.(2022); Mucoadhesive silver nanoparticle-based local drug delivery system for peri-implantitis management in COVID-19 era. Part 1: antimicrobial and safety in-vitro analysis. Journal of Oral Biology and Craniofacial Research,E3 ,1177-181 | Excluded by title |
| 211 | Di Gianfilippo, R. and Wang, C. W. and Xie, Y. Y. and Kinney, J. and Sugai, J. and Giannobile, W. V. and Wang, H. L.(2023); Effect of laser-assisted reconstructive surgical therapy of peri-implantitis on protein biomarkers and bacterial load. Clinical Oral Implants Research,E3 ,4393-403 | Excluded by title |
| 212 | Diachkova, E. and Corbella, S. and Taschieri, S. and Tarasenko, S.(2020); Nonsurgical treatment of peri-implantitis: Case series. Dentistry Journal,E3 ,3 | Excluded by title |
| 213 | Dommisch, H. and Hoedke, D. and Valles, C. and Vilarrasa, J. and Jepsen, S. and Pascual La Rocca, A.(2023); Efficacy of professionally administered chemical agents as an adjunctive treatment to sub-marginal instrumentation during the therapy of peri-implant mucositis. J Clin Periodontol,E3 ,146-160 | Excluded by title |
| 214 | Drks(2023); Effect of soft tissue augmentation with autologeous epithelialized connective tissue grafts in the treatment of periimplantitis: a randomised controlled clinical trial. https://trialsearch.who.int/Trial2.aspx?TrialID=DRKS00032251,E3 , | Excluded by title |
| 215 | Drks(2023); Integration of dentistry into multiprofessional care and discharge management of elderly multimorbid patients of the university geriatric medicine of the University Hospital Cologne - The IntDENTgration Study. https://trialsearch.who.int/Trial2.aspx?TrialID=DRKS00027438,E3 , | Excluded by title |
| 216 | Drks(2024); Effect of adjunctive electrochemical implant surface decontamination in the treatment of periimplantitis: a randomised controlled clinical trial. https://trialsearch.who.int/Trial2.aspx?TrialID=DRKS00033883,E3 , | Excluded by title |
| 217 | Drks. Repeated non-surgical periimplantitis therapy with two different antiseptic adjuncts. A randomized controlled clinical trial. 2015. | Excluded by title |
| 218 | Dumitriu, A. S. and Paunica, S. and Nicolae, X. A. and Bodnar, D. C. and Albu, S. D. and Suciu, I. and Ciongaru, D. N. and Giurgiu, M. C.(2023); The Effectiveness of the Association of Chlorhexidine with Mechanical Treatment of Peri-Implant Mucositis. Healthcare,E3 ,1312 | Excluded by title |
| 219 | E. A. Mancini and G. P. Pini Prato. Procedimiento de descontaminación del biofilm para el tratamiento del absceso periodontal agudo y la periimplantitis. 2016. | Excluded by title |
| 220 | E. Anitua, M. Troya, M. Zalduendo, R. Tejero and G. Orive. Progress in the use of autologous regenerative platelet-based therapies in implant dentistry. 2016. | Excluded by title |
| 221 | E. Anitua, R. Tejero, M. H. Alkhraisat and G. Orive. Platelet-rich plasma to improve the bio-functionality of biomaterials. 2013. | Excluded by title |
| 222 | E. Anitua. Implantes inmediatos post-extracción con carga inmediata en alveolos con infección por periodontitis activa: estudio de cohortes retrospectivo. 2017. | Excluded by title |
| 223 | E. B. Kenney and S. A. Jovanovic. Osteopromotion as an adjunct to osseointegration. 1993. | Excluded by title |
| 224 | E. C. Carlo Reis, A. P. B. Borges, M. V. F. Araújo, V. C. Mendes, L. Guan and J. E. Davies. Periodontal regeneration using a bilayered PLGA/calcium phosphate construct. 2011. | Excluded by title |
| 225 | E. Cianci, O. Trubiani, F. Diomede, I. Merciaro, I. Meschini, P. Bruni, F. Croce and M. Romano. Immobilization and delivery of biologically active Lipoxin A<inf>4</inf>using electrospinning technology. 2016. | Excluded by title |
| 226 | E. E. Machtei. Treatment Alternatives to Negotiate Peri-Implantitis. 2014. | Excluded by title |
| 227 | E. Figuero, F. Graziani, I. Sanz, D. Herrera and M. Sanz. Management of peri-implant mucositis and peri-implantitis. 2014. | Excluded by title |
| 228 | E. García-Gareta, M. J. Coathup and G. W. Blunn. Osteoinduction of bone grafting materials for bone repair and regeneration. 2015. | Excluded by title |
| 229 | E. Gerits, I. Van der Massen, K. Vandamme, K. De Cremer, K. De Brucker, K. Thevissen, B. P. A. Cammue, S. Beullens, M. Fauvart, N. Verstraeten and J. Michiels. In vitro activity of the antiasthmatic drug zafirlukast against the oral pathogens Porphyromonas gingivalis and Streptococcus mutans. 2017. | Excluded by title |
| 230 | E. Gerits, P. Spincemaille, K. De Cremer, K. De Brucker, S. Beullens, K. Thevissen, B. P. A. Cammue, K. Vandamme, M. Fauvart, N. Verstraeten and J. Michiels. Repurposing AM404 for the treatment of oral infections by Porphyromonas gingivalis. 2017. | Excluded by title |
| 231 | E. Grageda. Platelet-rich plasma and bone graft materials: A review and a standardized research protocol. 2004. | Excluded by title |
| 232 | E. H. Abdulkareem, K. Memarzadeh, R. P. Allaker, J. Huang, J. Pratten and D. Spratt. Anti-biofilm activity of zinc oxide and hydroxyapatite nanoparticles as dental implant coating materials. 2015. | Excluded by title |
| 233 | E. J. Blom, J. Klein-Nulend, C. P. Klein, K. Kurashina, M. A. van Waas and E. H. Burger. Transforming growth factor-beta1 incorporated during setting in calcium phosphate cement stimulates bone cell differentiation in vitro. 2000. | Excluded by title |
| 234 | E. J. Hansen, S. Schou, F. Harder and E. Hjørting-Hansen. Outcome of implant therapy involving localised lateral alveolar ridge and/or sinus floor augmentation: A clinical and radiographic retrospective 1-year study. 2011. | Excluded by title |
| 235 | E. Luo, J. Hu, C. Bao, Y. Li, Q. Tu, D. Murray and J. Chen. Sustained release of adiponectin improves osteogenesis around hydroxyapatite implants by suppressing osteoclast activity in ovariectomized rabbits. 2012. | Excluded by title |
| 236 | E. Mielczarek-Badora and M. Szulc. Photodynamic Therapy and its Role in Periodontitis Treatment. 2013. | Excluded by title |
| 237 | E. Papathanasiou, M. Finkelman, J. Hanley and A. O. Parashis. Prevalence, Etiology and Treatment of Peri-Implant Mucositis and Peri-Implantitis: A Survey of Periodontists in the United States. 2016. | Excluded by title |
| 238 | E. Romeo, D. Lops, S. Storelli and M. Ghisolfi. Clinical peri-implant sounding accuracy in the presence of chronic inflammation of peri-implant tissues. Clinical observation study. 2009. | Excluded by title |
| 239 | E. S. El Chaar and Z. N. Jalbout. Regeneration of an osseous peri-implantitis lesion. 2002. | Excluded by title |
| 240 | E. Stein, J. Koehn, W. Sutter, C. Schmidl, V. Lezaic, G. Wendtlandt, F. Watzinger and D. Turhani. Phenothiazine chloride and soft laser light have a biostimulatory effect on human osteoblastic cells. 2009. | Excluded by title |
| 241 | E. Stellini, A. Migliorato, S. Mazzoleni, A. Mottola, L. Lombardi and G. A. Favero. [Topical treatment of peri-implantitis with metronidazole dental gel 25%. Clinical analysis and microbiological control]. 2000. | Excluded by title |
| 242 | E. Vanderleyden, S. Mullens, J. Luyten and P. Dubruel. Implantable (Bio)polymer coated titanium scaffolds: A review. 2012. | Excluded by title |
| 243 | Elad, S. and Keegan, R. and Fregnani, E. R. and Gavish, L. and Ottaviani, G. and Arany, P. and Zadik, Y.(2024); Immediate pain alleviation in oral mucositis and other oral ulcerative diseases through photobiomodulation therapy: the preemptive treatment concept. Quintessence International,E3 ,6482-493 | Excluded by title |
| 244 | Elhadidi, M. H. and Awad, S. and Elsheikh, H. A. and Tawfik, M. A.(2023); Comparison of Clinical Efficacy of Screw-retained Arch Bar vs Conventional Erich's Arch Bar in Maxillomandibular Fixation: a Randomized Clinical Trial. Journal of contemporary dental practice,E3 ,12928‚Äê935 | Excluded by title |
| 245 | Elsaadany, B. and Anayb, S. M. and Mashhour, K. and Yossif, M. and Zahran, F.(2024); Rebamipide gargle and benzydamine gargle in prevention and management of chemo-radiotherapy and radiotherapy-induced oral mucositis in head and neck cancer patients (randomized clinical trial). Bmc Oral Health,E3 ,19 | Excluded by title |
| 246 | Elsadek, M. F. and Almoajel, A. and Sonbol, A. M. and Aljarbou, H. M.(2023); Chloro-aluminum phthalocyanine-mediated photodynamic therapy improves peri-implant parameters and crevicular fluid cytokine levels in cigarette smokers with chronic hyperglycemia. Photodiagnosis and Photodynamic Therapy,E3 ,6 | Excluded by title |
| 247 | Esmat, S. A. and El-Sayed, N. M. and Fahmy, R. A.(2023); Vitamin C mesotherapy versus diode laser for the esthetic management of physiologic gingival hyperpigmentation: a randomized clinical trial. BMC oral health,E3 ,1899 | Excluded by title |
| 248 | Etemadi, A. and Sabri, H. and Enssi, M.(2023); Surgical reconstruction of peri-implantitis with adjunctive antimicrobial photodynamic therapy: A case report with 5-year follow-up. Clinical Advances in Periodontics,E3 ,7 | Excluded by title |
| 249 | F. A. Alshehri. The role of lasers in the treatment of peri-implant diseases: A review. 2016. | Excluded by title |
| 250 | F. Alqahtani, M. Alqahtani, S. S. Shafqat, Z. Akram, A. A. Al-Kheraif and F. Javed. Efficacy of mechanical debridement with adjunctive probiotic therapy in the treatment of peri-implant mucositis in cigarette-smokers and never-smokers. 2019. | Excluded by title |
| 251 | F. Baino and I. Potestio. Orbital implants: State-of-the-art review with emphasis on biomaterials and recent advances. 2016. | Excluded by title |
| 252 | F. Bassi, P. P. Poli, D. Rancitelli, F. Signorino and C. Maiorana. Surgical Treatment of Peri-Implantitis: A 17-Year Follow-Up Clinical Case Report. 2015. | Excluded by title |
| 253 | F. Cairo, I. Sanz, P. Matesanz, M. Nieri and U. Pagliaro. Quality of reporting of randomized clinical trials in implant dentistry. A systematic review on critical aspects in design, outcome assessment and clinical relevance. 2012. | Excluded by title |
| 254 | F. Chellini, C. Sassoli, D. Nosi, C. Deledda, P. Tonelli, S. Zecchi-Orlandini, L. Formigli and M. Giannelli. Low pulse energy Nd:YAG laser irradiation exerts a biostimulative effect on different cells of the oral microenvironment: "an in vitro study". 2010. | Excluded by title |
| 255 | F. Chellini, M. Giannelli, A. Tani, L. Ballerini, L. Vallone, D. Nosi, S. Zecchi-Orlandini and C. Sassoli. Mesenchymal stromal cell and osteoblast responses to oxidized titanium surfaces pre-treated with lambda = 808 nm GaAlAs diode laser or chlorhexidine: in vitro study. 2017. | Excluded by title |
| 256 | F. De Siena, L. Francetti, S. Corbella, S. Taschieri and M. Del Fabbro. Topical application of 1% chlorhexidine gel versus 0.2% mouthwash in the treatment of peri-implant mucositis. An observational study. 2013. | Excluded by title |
| 257 | F. De Siena, S. Corbella, S. Taschieri, M. Del Fabbro and L. Francetti. Adjunctive glycine powder air-polishing for the treatment of peri-implant mucositis: an observational clinical trial. 2015. | Excluded by title |
| 258 | F. Di Carlo, A. Quaranta, L. Di Alberti, L. F. Ronconi, M. Quaranta and A. Piattelli. Influence of amine fluoride/stannous fluoride mouthwashes with and without chlorhexidine on secretion of proinflammatory molecules by peri-implant crevicular fluid cells. 2008. | Excluded by title |
| 259 | F. G. Draenert, A. L. Nonnenmacher, P. W. Kämmerer, J. Goldschmitt and W. Wagner. BMP-2 and bFGF release and in vitro effect on human osteoblasts after adsorption to bone grafts and biomaterials. 2013. | Excluded by title |
| 260 | F. G. Draenert, P. W. Kämmerer, V. Palarie and W. Wagner. Vertical Bone Augmentation with Simultaneous Dental Implantation Using Crestal Biomaterial Rings: A Rabbit Animal Study. 2012. | Excluded by title |
| 261 | F. Javed and G. E. Romanos. Does photodynamic therapy enhance standard antibacterial therapy in dentistry?. 2013. | Excluded by title |
| 262 | F. Javed, A. S. Alghamdi, A. Ahmed, T. Mikami, H. B. Ahmed and H. C. Tenenbaum. Clinical efficacy of antibiotics in the treatment of peri-implantitis. 2013. | Excluded by title |
| 263 | F. Javed, H. A. Hussain and G. E. Romanos. Re-stability of dental implants following treatment of peri-implantitis. 2013. | Excluded by title |
| 264 | F. Javed, M. S. BinShabaib, S. S. Alharthi and T. Qadri. Role of mechanical curettage with and without adjunct antimicrobial photodynamic therapy in the treatment of peri-implant mucositis in cigarette smokers: A randomized controlled clinical trial. 2017. | Excluded by title |
| 265 | F. Javed, T. Abduljabbar, G. Carranza, E. Gholamiazizi, D. K. Mazgaj, S. V. Kellesarian and F. Vohra. Efficacy of periimplant mechanical debridement with and without adjunct antimicrobial photodynamic therapy in the treatment of periimplant diseases among cigarette smokers and non-smokers. 2016. | Excluded by title |
| 266 | F. Lerario, M. Roncati, A. Gariffo, E. Attorresi, A. Lucchese, A. Galanakis, G. Palaia and U. Romeo. Erratum to: Non-surgical periodontal treatment of peri-implant diseases with the adjunctive use of diode laser: preliminary clinical study. 2016. | Excluded by title |
| 267 | F. Lerario, M. Roncati, A. Gariffo, E. Attorresi, A. Lucchese, A. Galanakis, G. Palaia and U. Romeo. Non-surgical periodontal treatment of peri-implant diseases with the adjunctive use of diode laser: preliminary clinical study. 2016. | Excluded by title |
| 268 | F. Lupi, C. Lorenzi, M. Ricci and A. Genovesi. Use of slow-release products in perimplantitis. 2012. | Excluded by title |
| 269 | F. M. Chen, R. Chen, X. J. Wang, H. H. Sun and Z. F. Wu. In vitro cellular responses to scaffolds containing two microencapulated growth factors. 2009. | Excluded by title |
| 270 | F. Muller. Interventions for edentate elders--what is the evidence?. 2014. | Excluded by title |
| 271 | F. Obadan, S. Craitoiu, H. O. Manolea, M. C. Hincu and M. M. Iacov-Craitoiu. The evaluation of the morphological evolution of the tissue integration of dental implants through conventional histology and immunohistochemistry techniques. 2018. | Excluded by title |
| 272 | F. P. Jorand, S. Debuy, S. F. Kamagate and M. Engels-Deutsch. Evaluation of a biofilm formation by Desulfovibrio fairfieldensis on titanium implants. 2015. | Excluded by title |
| 273 | F. P. Strietzel, S. Rothe, P. A. Reichart and A. M. Schmidt-Westhausen. Implant-prosthetic treatment in HIV-infected patients receiving highly active antiretroviral therapy: Report of cases. 2006. | Excluded by title |
| 274 | F. Pakdel, S. Ghasemi, A. Babaloo, Y. Javadzadeh, R. Momeni, M. Ghanizadeh, S. R. Moaddab and F. Y. Fathi. Antibacterial Effects of Garlic Extracts and Ziziphora Essential Oil on Bacteria Associated with Peri-Implantitis. 2017. | Excluded by title |
| 275 | F. Schwarz, K. Becker and M. Sager. Efficacy of professionally administered plaque removal with or without adjunctive measures for the treatment of peri-implant mucositis. A systematic review and meta-analysis. 2015. | Excluded by title |
| 276 | F. Schwarz, K. Becker and S. Renvert. Efficacy of air polishing for the non-surgical treatment of peri-implant diseases: a systematic review. 2015. | Excluded by title |
| 277 | F. Schwarz, S. Jepsen, M. Herten, M. Sager, D. Rothamel and J. Becker. Influence of different treatment approaches on non-submerged and submerged healing of ligature induced peri-implantitis lesions: an experimental study in dogs. 2006. | Excluded by title |
| 278 | F. Schwendicke, Y. K. Tu and M. Stolpe. Preventing and Treating Peri-Implantitis: A Cost-Effectiveness Analysis. 2015. | Excluded by title |
| 279 | F. Sgolastra, A. Petrucci, M. Severino, R. Gatto and A. Monaco. Periodontitis, implant loss and peri-implantitis. A meta-analysis. 2015. | Excluded by title |
| 280 | F. Suarez, A. Monje, P. Galindo-Moreno and H. L. Wang. Implant surface detoxification: a comprehensive review. 2013. | Excluded by title |
| 281 | F. Suarez-Lopez Del Amo, S. H. Yu and H. L. Wang. Non-Surgical Therapy for Peri-Implant Diseases: a Systematic Review. 2016. | Excluded by title |
| 282 | F. Verdugo, T. Laksmana and A. Uribarri. Systemic antibiotics and the risk of superinfection in peri-implantitis. 2016. | Excluded by title |
| 283 | F. Vohra, M. Q. Al-Rifaiy, G. Lillywhite, M. I. Abu Hassan and F. Javed. Efficacy of mechanical debridement with adjunct antimicrobial photodynamic therapy for the management of peri-implant diseases: a systematic review. 2014. | Excluded by title |
| 284 | F. Vohra, Z. Akram, S. H. Safii, R. D. Vaithilingam, A. Ghanem, K. Sergis and F. Javed. Role of antimicrobial photodynamic therapy in the treatment of aggressive periodontitis: A systematic review. 2016. | Excluded by title |
| 285 | Falahinia, N. and Razeghi, S. and Shamshiri, A. R. and Firoozi, M. and Mohebbi, S. Z.(2023); The effectiveness of motivational interviewing on the oral health of leukemic children and oral health care knowledge, attitude and practice of their mothers: a hospital-based intervention. BMC pediatrics,E3 ,1261 | Excluded by title |
| 286 | Feng, J. and Zhou, Q. and Nie, J.(2024); Effects of Polymeric Ceramic Inlay and Full Crown Restoration on Patients with Dental Defects. Journal of Hard Tissue Biology,E3 ,2125-130 | Excluded by title |
| 287 | G. A. Kotsakis, I. Konstantinidis, I. K. Karoussis, X. Ma and H. Chu. Systematic review and meta-analysis of the effect of various laser wavelengths in the treatment of peri-implantitis. 2014. | Excluded by title |
| 288 | G. A. Mandelaris and S. D. Vlk. Guided implant surgery with placement of a presurgical CAD/CAM patient-specific abutment and provisional in the esthetic zone. 2014. | Excluded by title |
| 289 | G. A. Silva, O. P. Coutinho, P. Ducheyne and R. L. Reis. Materials in particulate form for tissue engineering. 2. Applications in bone. 2007. | Excluded by title |
| 290 | G. Avila-Ortiz, P. Mark Bartold, W. Giannobile, W. Katagiri, S. Nares, H. Rios, D. Spagnoli and U. M. E. Wikesjö. Biologics and cell therapy tissue engineering approaches for the management of the edentulous maxilla: A systematic review. 2016. | Excluded by title |
| 291 | G. Brunello, S. Sivolella, R. Meneghello, L. Ferroni, C. Gardin, A. Piattelli, B. Zavan and E. Bressan. Powder-based 3D printing for bone tissue engineering. 2016. | Excluded by title |
| 292 | G. Chacón and C. Gallego. Peri-implantitis: revisión de la literatura. 2012. | Excluded by title |
| 293 | G. D. Stynes, G. K. Kiroff, R. S. Page, W. A. Morrison and M. A. Kirkland. Surface-bound collagen 4 is significantly more stable than collagen 1. 2017. | Excluded by title |
| 294 | G. E. Chacon, E. A. Stine, P. E. Larsen, F. M. Beck and E. A. McGlumphy. Effect of alendronate on endosseous implant integration: an in vivo study in rabbits. 2006. | Excluded by title |
| 295 | G. E. Romanos, F. Javed, R. A. Delgado-Ruiz and J. L. Calvo-Guirado. Peri-implant diseases: a review of treatment interventions. 2015. | Excluded by title |
| 296 | G. E. Salvi and C. A. Ramseier. Efficacy of patient-administered mechanical and/or chemical plaque control protocols in the management of peri-implant mucositis. A systematic review. 2015. | Excluded by title |
| 297 | G. E. Salvi, B. Carollo-Bittel and N. P. Lang. Effects of diabetes mellitus on periodontal and peri-implant conditions: update on associations and risks. 2008. | Excluded by title |
| 298 | G. E. Salvi, R. C. Williams and S. Offenbacher. Nonsteroidal anti-inflammatory drugs as adjuncts in the management of periodontal diseases and peri-implantitis. 1997. | Excluded by title |
| 299 | G. Favia, A. Tempesta, L. Limongelli, V. Crincoli, A. Piattelli and E. Maiorano. Metastatic Breast Cancer in Medication-Related Osteonecrosis Around Mandibular Implants. 2015. | Excluded by title |
| 300 | G. Fernandez de Grado, L. Keller, Y. Idoux-Gillet, Q. Wagner, A. M. Musset, N. Benkirane-Jessel, F. Bornert and D. Offner. Bone substitutes: a review of their characteristics, clinical use, and perspectives for large bone defects management. 2018. | Excluded by title |
| 301 | G. Gomez-Moreno, A. Aguilar-Salvatierra, J. Rubio Roldan, J. Guardia, J. Gargallo and J. L. Calvo-Guirado. Peri-implant evaluation in type 2 diabetes mellitus patients: a 3-year study. 2015. | Excluded by title |
| 302 | G. H. Lin, F. Suarez Lopez Del Amo and H. L. Wang. Laser therapy for treatment of peri-implant mucositis and peri-implantitis: An American Academy of Periodontology best evidence review. 2018. | Excluded by title |
| 303 | G. Hernandez, V. Paredes, R. M. Lopez-Pintor, A. de Andres, J. C. de Vicente and M. Sanz. Implant treatment in immunosuppressed renal transplant patients: A prospective case-controlled study. 2019. | Excluded by title |
| 304 | G. Iezzi, A. Piattelli, A. Giuliani, C. Mangano, A. Barone, L. Manzon, M. Degidi, A. Scarano, A. Filippone and V. Perrotti. Molecular, cellular and pharmaceutical aspects of bone grafting materials and membranes during maxillary sinus-lift procedures. Part 2: Detailed characteristics of the materials. 2017. | Excluded by title |
| 305 | G. Iezzi, A. Scarano, G. Petrone and A. Piattelli. Two human hydroxyapatite-coated dental implants retrieved after a 14-year loading period: a histologic and histomorphometric case report. 2007. | Excluded by title |
| 306 | G. Intini. The use of platelet-rich plasma in bone reconstruction therapy. 2009. | Excluded by title |
| 307 | G. J. van der Putten, L. De Visschere, C. van der Maarel-Wierink, J. Vanobbergen and J. Schols. The importance of oral health in (frail) elderly people - a review. 2013. | Excluded by title |
| 308 | G. John, J. Becker and F. Schwarz. Effectivity of air-abrasive powder based on glycine and tricalcium phosphate in removal of initial biofilm on titanium and zirconium oxide surfaces in an ex vivo model. 2016. | Excluded by title |
| 309 | G. Koukos, C. Papadopoulos, L. Tsalikis, D. Sakellari, M. Arsenakis and A. Konstantinidis. Prevalence of antibiotic resistance genes in subjects with successful and failing dental implants. A pilot study. 2014. | Excluded by title |
| 310 | G. N. Hasanoglu Erbasar, T. P. Hocaoglu and R. C. Erbasar. Risk factors associated with short dental implant success: a long-term retrospective evaluation of patients followed up for up to 9 years. 2019. | Excluded by title |
| 311 | G. Patianna, N. A. Valente, A. D'Addona and S. Andreana. In vitro evaluation of controlled-release 14% doxycycline gel for decontamination of machined and sandblasted acid-etched implants. 2018. | Excluded by title |
| 312 | G. Pini-Prato, C. Magnani and R. Rotundo. Nonsurgical Treatment of Peri-implantitis Using the Biofilm Decontamination Approach: A Case Report Study. 2016. | Excluded by title |
| 313 | G. Polimeni, U. M. Wikesjo, C. Susin, M. Qahash, R. H. Shanaman, H. S. Prasad, M. D. Rohrer and J. Hall. Alveolar ridge augmentation using implants coated with recombinant human growth/differentiation factor-5: histologic observations. 2010. | Excluded by title |
| 314 | G. R. Mettraux, A. Sculean, W. B. Burgin and G. E. Salvi. Two-year clinical outcomes following non-surgical mechanical therapy of peri-implantitis with adjunctive diode laser application. 2016. | Excluded by title |
| 315 | G. Ramalho-Ferreira, L. P. Faverani, F. B. Prado, I. R. Garcia, Jr. and R. Okamoto. Raloxifene enhances peri-implant bone healing in osteoporotic rats. 2015. | Excluded by title |
| 316 | G. Schenk, T. F. Flemmig, T. Betz, J. Reuther and B. Klaiber. Controlled local delivery of tetracycline HCl in the treatment of periimplant mucosal hyperplasia and mucositis. A controlled case series. 1997. | Excluded by title |
| 317 | G. Sivaramakrishnan and K. Sridharan. Photodynamic therapy for the treatment of peri-implant diseases: A network meta-analysis of randomized controlled trials. 2018. | Excluded by title |
| 318 | G. Tawil and P. Tawil. Peri-implant Infection Concomitant with a Flare-up Episode of Chronic Periodontitis: An Unusual Regeneration Following Treatment and a 5-Year Follow-up. 2019. | Excluded by title |
| 319 | G. Turnbull, J. Clarke, F. Picard, P. Riches, L. Jia, F. Han, B. Li and W. Shu. 3D bioactive composite scaffolds for bone tissue engineering. 2018. | Excluded by title |
| 320 | G. Verardi, M. S. Cenci, T. T. Maske, B. Webber and L. R. Santos. Antiseptics and microcosm biofilm formation on titanium surfaces. 2016. | Excluded by title |
| 321 | G√∂ltz, M. and Koch, M. and Detsch, R. and Karl, M. and Burkovski, A. and Rosiwal, S.(2019); Influence of in-situ electrochemical oxidation on implant surface and colonizing microorganisms evaluated by scanning electron microscopy. Materials,E3 ,23 | Excluded by title |
| 322 | g2q8q, R. B. R.(2023); The effect of Cannabinoids on Oral Mucositis during Cancer treatment. https://trialsearch.who.int/Trial2.aspx?TrialID=RBR-8g2q8q5,E3 , | Excluded by title |
| 323 | Gholami, L. and Shahabi, S. and Jazaeri, M. and Hadilou, M. and Fekrazad, R.(2023); Clinical applications of antimicrobial photodynamic therapy in dentistry. Frontiers in Microbiology,E3 ,47 | Excluded by title |
| 324 | Gil Gonz√°lez, J. and N√∫√±ez M√°rquez, E. and Moreno Mu√±oz, J. and Matos Garrido, N. and Jim√©nez Guerra, A. and Monsalve Guil, L. and Ortiz Garc√≠a, I. and Velasco Ortega, E.(2021); Long-term clinical efficacy of implants with internal connection and sandblasted-acid-etched surface. Avances en Odontoestomatologia,E3 ,143405 | Excluded by title |
| 325 | Gomes, S. C. and Corvello, P. and Romagna, R. and M√ºller, L. H. and Angst, P. D. M. and Oppermann, R. V.(2015); How do peri-implant mucositis and gingivitis respond to supragingival biofilm control an intra-individual longitudinal cohort study. European Journal of Oral Implantology,E3 ,165-73 | Excluded by title |
| 326 | Gomes-Silva, W. and Vechiato, A. J. and Luiz, A. C. and Guollo, A. and de Oliveira, M. C. Q. and Gomes, M. N. and Caparelli, F. C. and Brandao, T. B.(2023); Clinical characterization of stomatitis cases with an epithelial growth factor receptor inhibitor in metastatic colorectal cancer patients: A study of 7 cases and literature review. Oral Surgery Oral Medicine Oral Pathology Oral Radiology,E3 ,2162-172 | Excluded by title |
| 327 | Gong, Z. J. and Lin, Y. and Di, P.(2023); Plaque accumulation on the fitting surface of full-arch implant-supported fixed prostheses with contact or noncontact pontics: A split mouth randomized controlled trial. Journal of Esthetic and Restorative Dentistry,E3 ,71077-1084 | Excluded by title |
| 328 | Graterol-Duran, A. and Ayuso-Montero, R. and Saka-Herr√°n, C. and Blazquez-Hinarejos, M. and Roca-Obis, P. and Mar√≠-Roig, A. and L√≥pez-L√≥pez, J.(2022); Retrospective Study of Biohorizons¬Æ Implants Placed by Postgraduate Students at the University of Barcelona. Applied Sciences (Switzerland),E3 ,6 | Excluded by title |
| 329 | Guarnieri, R. and Reda, R. and Zanza, A. and Xhajanka, E. and Patil, S. and Nardo, D. D. and Testarelli, L.(2024); Relationship between gingival and peri-implant sulcular fluid active matrix metalloproteinase-8 concentration and clinical indices in healthy and diseased conditions. Exploration of Medicine,E3 ,2243-256 | Excluded by title |
| 330 | Guo, J. and Chen, X. and Xie, H. and Li, T.(2024); Efficacy of adjunctive photodynamic therapy to conventional mechanical debridement for peri-implant mucositis. BMC Oral Health,E3 ,1464 | Excluded by title |
| 331 | Gupta, B. and Gupta, S. and Divya, D. and Dev, S. V. and Bhola, S. and Guruprasad, Y.(2024); Comparing the Effectiveness of Different Techniques for the Management of Dental Implant Peri‚ÄëImplant Mucositis: a Randomized Controlled Trial. Journal of pharmacy & bioallied sciences,E3 ,S868‚ÄêS870 | Excluded by title |
| 332 | H. Aludden, A. Mordenfeld, M. Hallman, A. E. Christensen and T. Starch-Jensen. Osteotome-Mediated Sinus Floor Elevation With or Without a Grafting Material: A Systematic Review and Meta-analysis of Long-term Studies (>/=5-Years). 2018. | Excluded by title |
| 333 | H. Arab, F. Shiezadeh, A. Moeintaghavi, N. Anbiaei and S. Mohamadi. Comparison of Two Regenerative Surgical Treatments for Peri-Implantitis Defect using Natix Alone or in Combination with Bio-Oss and Collagen Membrane. 2016. | Excluded by title |
| 334 | H. Arakawa, J. Uehara, E. S. Hara, W. Sonoyama, A. Kimura, M. Kanyama, Y. Matsuka and T. Kuboki. Matrix metalloproteinase-8 is the major potential collagenase in active peri-implantitis. 2012. | Excluded by title |
| 335 | H. C. Lim, J. S. Lee, S. H. Choi and U. W. Jung. The effect of overlaying titanium mesh with collagen membrane for ridge preservation. 2015. | Excluded by title |
| 336 | H. Gursoy, C. Ozcakir-Tomruk, J. Tanalp and S. Yilmaz. Photodynamic therapy in dentistry: a literature review. 2013. | Excluded by title |
| 337 | H. Hallstrom, G. R. Persson, S. Lindgren, M. Olofsson and S. Renvert. Systemic antibiotics and debridement of peri-implant mucositis. A randomized clinical trial. 2012. | Excluded by title |
| 338 | H. Hallstrom, S. Lindgren, C. Widen, S. Renvert and S. Twetman. Probiotic supplements and debridement of peri-implant mucositis: a randomized controlled trial. 2016. | Excluded by title |
| 339 | H. He, Y. Yao, Y. Wang, Y. Wu, Y. Yang and P. Gong. A novel bionic design of dental implant for promoting its long-term success using nerve growth factor (NGF): utilizing nano-springs to construct a stress-cushioning structure inside the implant. 2012. | Excluded by title |
| 340 | H. Liu, W. Li, C. Liu, J. Tan, H. Wang, B. Hai, H. Cai, H. J. Leng, Z. J. Liu and C. L. Song. Incorporating simvastatin/poloxamer 407 hydrogel into 3D-printed porous Ti <inf>6</inf> Al <inf>4</inf> V scaffolds for the promotion of angiogenesis, osseointegration and bone ingrowth. 2016. | Excluded by title |
| 341 | H. Peng, X. Liu, R. Wang, F. Jia, L. Dong and Q. Wang. Emerging nanostructured materials for musculoskeletal tissue engineering. 2014. | Excluded by title |
| 342 | H. S. Alghamdi, R. Bosco, S. K. Both, M. Iafisco, S. C. Leeuwenburgh, J. A. Jansen and J. J. van den Beucken. Synergistic effects of bisphosphonate and calcium phosphate nanoparticles on peri-implant bone responses in osteoporotic rats. 2014. | Excluded by title |
| 343 | H. S. Kim, J. I. Lee, S. S. Yang, B. S. Kim, B. C. Kim and J. Lee. The effect of alendronate soaking and ultraviolet treatment on bone-implant interface. 2017. | Excluded by title |
| 344 | H. S. Ryu, Y. I. Kim, B. S. Lim, Y. J. Lim and S. J. Ahn. Chlorhexidine Uptake and Release From Modified Titanium Surfaces and Its Antimicrobial Activity. 2015. | Excluded by title |
| 345 | H. Schliephake, A. Sicilia, B. A. Nawas, N. Donos, R. Gruber, S. Jepsen, I. Milinkovic, A. Mombelli, J. M. Navarro, M. Quirynen, I. Rocchietta, M. Schiodt, S. Schou, A. Stahli and A. Stavropoulos. Drugs and diseases: Summary and consensus statements of group 1. The 5(th) EAO Consensus Conference 2018. 2018. | Excluded by title |
| 346 | H. Schliephake. Clinical efficacy of growth factors to enhance tissue repair in oral and maxillofacial reconstruction: A systematic review. 2015. | Excluded by title |
| 347 | H. Storrie and S. I. Stupp. Cellular response to zinc-containing organoapatite: An in vitro study of proliferation, alkaline phosphatase activity and biomineralization. 2005. | Excluded by title |
| 348 | H. Tang, B. Wu, X. Qin, L. Zhang, J. Kretlow and Z. Xu. Tissue engineering rib with the incorporation of biodegradable polymer cage and BMSCs/decalcified bone: An experimental study in a canine model. 2013. | Excluded by title |
| 349 | H. Y. Kim, J. H. Park, J. H. Byun, J. H. Lee and S. H. Oh. BMP-2-Immobilized Porous Matrix with Leaf-Stacked Structure as a Bioactive GBR Membrane. 2018. | Excluded by title |
| 350 | H. Yu, L. Chen, Y. Zhu and L. Qiu. Bilamina cortical tenting grafting technique for three-dimensional reconstruction of severely atrophic alveolar ridges in anterior maxillae: A 6-year prospective study. 2016. | Excluded by title |
| 351 | Hallstr√∂m, H. and Persson, G. R. and Lindgren, S. and Olofsson, M. and Renvert, S.(2012); Systemic antibiotics and debridement of peri-implant mucositis. A randomized clinical trial. Journal of Clinical Periodontology,E3 ,6574-581 | Excluded by title |
| 352 | Hammerle, C. H. F. and Jepsen, K. and Sailer, I. and Strasding, M. and Zeltner, M. and Cordaro, L. and Mirisola di Torresanto, V. and Schwarz, F. and Zuhr, O. and Akakpo, D. and et al.(2023); Efficacy of a collagen matrix for soft tissue augmentation after implant placement compared to connective tissue grafts: a multicenter, noninferiority, randomized controlled trial. Clinical oral implants research,E3 ,9999‚Äê1013 | Excluded by title |
| 353 | Han, Q. and Jiang, Y. and Brandt, B. W. and Yang, J. and Chen, Y. and Buijs, M. J. and Crielaard, W. and Cheng, L. and Deng, D.(2019); Regrowth of Microcosm Biofilms on Titanium Surfaces After Various Antimicrobial Treatments. Frontiers in Microbiology,E3 , | Excluded by title |
| 354 | Harrison, P. and Madeley, E. and Nolan, M. and Renvert, S. and Polyzois, I.(2024); A longitudinal analysis of the impact of nonsurgical and surgical treatment of peri-implantitis upon clinical parameters and implant stability quotient values. A 2-3-year follow-up. Clinical and Experimental Dental Research,E3 ,111 | Excluded by title |
| 355 | Harrison, P. and Madeley, E. and Nolan, M. and Renvert, S. and Polyzois, I.(2024); A longitudinal analysis of the impact of nonsurgical and surgical treatment of peri-implantitis upon clinical parameters and implant stability quotient values.¬†A 2-3-year follow-up. Clin Exp Dent Res,E3 ,1e833 | Excluded by title |
| 356 | Hashim, A. and Kheir El Din, N. H. and El-Khazragy, N. and Almalahy, H. G.(2024); Comparison of the efficacy of Er,Cr:YSGG laser on oral biofilm removal from implant surfaces with various application times for the treatment of peri-implantitis defects: ex vivo study. BMC Oral Health,E3 ,1980 | Excluded by title |
| 357 | Hassan, N. A. and Al-Jaboori, A. S. K. and Al-Radha, A. S. D.(2022); Evaluation of Cortical Bone Thickness of Posterior Implant Sites Using CBCT in Iraqi Population. International Journal of Dentistry,E3 , | Excluded by title |
| 358 | He, G. and Wang, Z. and Hu, C. and Yang, Y. and Wang, N. and Shao, L. and You, J.(2023); The effect of motivational interviewing based on the transtheoretical model on oral cleaning behavior of patients with periodontitis who have undergone implant restoration. Technology and health care,E3 ,541‚Äê549 | Excluded by title |
| 359 | Heo, S. and Kim, H. J. and Joo, J. Y. and Lee, J. and Kim, S. J. and Choi, J.(2018); Simplified nonsurgical treatment of peri-implantitis using chlorhexidine and minocycline hydrochloride. Journal of Periodontal and Implant Science,E3 ,5326-333 | Excluded by title |
| 360 | Horv√°th, A. and Windisch, P. and Palkovics, D. and Li, X.(2024); Novel Technique to Reconstruct Peri-Implant Keratinised Mucosa Width Using Xenogeneic Dermal Matrix. Clinical Case Series. Dentistry Journal,E3 ,3 | Excluded by title |
| 361 | hq4kd, R. B. R.(2024); The effect of Stannous Fluoride Toothpaste in the treatment of Experimental Peri-implant Mucositis and Gingivitis: a randomized, double-blind, parallel-arm clinical trial. https://trialsearch.who.int/Trial2.aspx?TrialID=RBR-4hq4kd8,E3 , | Excluded by title |
| 362 | Hu, B. and Qiao, W. and Cao, Y. and Fu, X. and Song, J.(2024); A sono-responsive antibacterial nanosystem co-loaded with metformin and bone morphogenetic protein-2 for mitigation of inflammation and bone loss in experimental peri-implantitis. Frontiers in Bioengineering and Biotechnology,E3 , | Excluded by title |
| 363 | Huang, X. and Zhou, W. and Zhou, X. and Hu, Y. and Xiang, P. and Li, B. and Yang, B. and Peng, X. and Ren, B. and Li, M. and Cheng, L.(2019); Effect of novel micro-arc oxidation implant material on preventing peri-implantitis. Coatings,E3 ,11 | Excluded by title |
| 364 | Hussain, B. and Grytten, J. I. and Rongen, G. and Sanz, M. and Haugen, H. J.(2024); Surface Topography Has Less Influence on Peri-Implantitis than Patient Factors: A Comparative Clinical Study of Two Dental Implant Systems. Acs Biomaterials Science & Engineering,E3 ,74562-4574 | Excluded by title |
| 365 | I. A. Rodriguez, E. A. Growney Kalaf, G. L. Bowlin and S. A. Sell. Platelet-rich plasma in bone regeneration: Engineering the delivery for improved clinical efficacy. 2014. | Excluded by title |
| 366 | I. Atsuta, Y. Ayukawa, A. Furuhashi, T. Yamaza, Y. Tsukiyama and K. Koyano. Promotive effect of insulin-like growth factor-1 for epithelial sealing to titanium implants. 2013. | Excluded by title |
| 367 | I. Brook, M. A. O. Lewis, G. K. B. Sandor, M. Jeffcoat, L. P. Samaranayake and J. V. Rojas. Clindamycin in dentistry: More than just effective prophylaxis for endocarditis?. 2005. | Excluded by title |
| 368 | I. Darby. Periodontal considerations in older individuals. 2015. | Excluded by title |
| 369 | I. Ericsson, L. G. Persson, T. Berglundh, T. Edlund and J. Lindhe. The effect of antimicrobial therapy on periimplantitis lesions. An experimental study in the dog. 1996. | Excluded by title |
| 370 | I. Giovannacci, M. Meleti, M. Manfredi, C. Mortellaro, A. Greco Lucchina, M. Bonanini and P. Vescovi. Medication-Related Osteonecrosis of the Jaw Around Dental Implants: Implant Surgery-Triggered or Implant Presence-Triggered Osteonecrosis?. 2016. | Excluded by title |
| 371 | I. Hauser-Gerspach, J. Vadaszan, I. Deronjic, C. Gass, J. Meyer, M. Dard, T. Waltimo, S. Stubinger and C. Mauth. Influence of gaseous ozone in peri-implantitis: bactericidal efficacy and cellular response. An in vitro study using titanium and zirconia. 2012. | Excluded by title |
| 372 | I. K. Karoussis, K. Kyriakidou, J. Papaparaskevas, I. A. Vrotsos, M. Simopoulou and G. A. Kotsakis. Osteostimulative calcium phosphosilicate biomaterials partially restore the cytocompatibility of decontaminated titanium surfaces in a peri-implantitis model. 2018. | Excluded by title |
| 373 | I. Kanwar, A. K. Sah and P. K. Suresh. Biofilm-mediated Antibiotic-resistant Oral Bacterial Infections: Mechanism and Combat Strategies. 2017. | Excluded by title |
| 374 | I. Kaplan, A. Hirshberg, B. Shlomi, O. Platner, A. Kozlovsky, R. Ofec and D. Schwartz-Arad. The importance of histopathological diagnosis in the management of lesions presenting as peri-implantitis. 2015. | Excluded by title |
| 375 | I. Koban, B. Holtfreter, N. O. Hubner, R. Matthes, R. Sietmann, E. Kindel, K. D. Weltmann, A. Welk, A. Kramer and T. Kocher. Antimicrobial efficacy of non-thermal plasma in comparison to chlorhexidine against dental biofilms on titanium discs in vitro - proof of principle experiment. 2011. | Excluded by title |
| 376 | I. Miyamoto, T. Takahashi, T. Tanaka, B. Hirayama, K. Tanaka, T. Yamazaki, Y. Morimoto and I. Yoshioka. Dense cancellous bone as evidenced by a high HU value is predictive of late implant failure: a preliminary study. 2018. | Excluded by title |
| 377 | Ibrahim, S. S. and Hassanein, F. E. A. and Zaky, H. W. and Gamal, H.(2024); Clinical and biochemical assessment of the effect of glutamine in management of radiation induced oral mucositis in patients with head and neck cancer: Randomized controlled clinical trial. Journal of Stomatology Oral and Maxillofacial Surgery,E3 ,38 | Excluded by title |
| 378 | Ihan Hren, N. and Kiteska, B. and Kopaƒç, I.(2013); Ten-year survival and success rate of implant-prosthodontic treatment. Zdravniski Vestnik,E3 ,11718-731 | Excluded by title |
| 379 | Inchingolo, A. M. and Malcangi, G. and Ferrante, L. and Del Vecchio, G. and Viapiano, F. and Inchingolo, A. D. and Mancini, A. and Annicchiarico, C. and Inchingolo, F. and Dipalma, G. and Minetti, E. and Palermo, A. and Patano, A.(2023); Surface Coatings of Dental Implants: A Review. Journal of Functional Biomaterials,E3 ,518 | Excluded by title |
| 380 | Iqbal, N. and Aziz, H. M. I. and Sultan, F. and Tabussam, S. and Zaheer, A. and Rafique, R.(2023); P-Cab Dependent Dual Therapy Versus PPI Dependent Triple Therapy Against H Pylori Infection in Acid Peptic Disease. Pakistan journal of medical and health sciences,E3 ,2675‚Äê676 | Excluded by title |
| 381 | Irct2013060113543N. the effect of emdogain on peri-implant mucosal inflammation (PIMI). 2013. | Excluded by title |
| 382 | Irct2013100114847N. Local antibiotic therapy in peri- implant inflammation. 2014. | Excluded by title |
| 383 | Irct201311103690N. Effects of minocycline and emdogain in the treatment of peri-implant mucosal inflammation. 2014. | Excluded by title |
| 384 | Irct2016092411770N. Effect of photodynamic therapy with toloeidene blue photosensitizer on nonsurgical management of peri-implant mucosal inflammation and Its matrix metalloproteinase 8 and Cytokine Profile. 2017. | Excluded by title |
| 385 | Irct20230628058607N(2023); Evaluating the effect of professional oral and dental health care and Preventive treatment on the reduction of oral and dental complications caused by radiotherapy and chemotherapy treatment in patients with head and neck cancers. https://trialsearch.who.int/Trial2.aspx?TrialID=IRCT20230628058607N1,E3 , | Excluded by title |
| 386 | Irct20231021059788N(2024); Comparison of the effect of V-One mouthwash (ginger) and Irsha mouthwash (ginger and yarrow) and nystatin in the treatment of denture osteomatitis type 2. https://trialsearch.who.int/Trial2.aspx?TrialID=IRCT20231021059788N1,E3 , | Excluded by title |
| 387 | Irct20231126060189N(2024); Evaluation of the effect of Zataria multiflora extract mouthwash on prevention of chemotherapy induced oral mucositis. https://trialsearch.who.int/Trial2.aspx?TrialID=IRCT20231126060189N1,E3 , | Excluded by title |
| 388 | Irct20240122060773N(2024); The effect of postbiotic mouthwash on oral ulcerative lichen planus lesions. https://trialsearch.who.int/Trial2.aspx?TrialID=IRCT20240122060773N1,E3 , | Excluded by title |
| 389 | Isrctn(2023); A comparative study between the antifungal activity of curcumin and nystatin in denture stomatitis (randomized controlled trial). https://trialsearch.who.int/Trial2.aspx?TrialID=ISRCTN15898291,E3 , | Excluded by title |
| 390 | Isrctn(2023); Clinical trial on the effectiveness of virgin coconut oil as an additional treatment for root scaling and debridement. https://trialsearch.who.int/Trial2.aspx?TrialID=ISRCTN10241517,E3 , | Excluded by title |
| 391 | Isrctn(2023); Efficacy and safety of topical tocopherols in oral biopsies. https://trialsearch.who.int/Trial2.aspx?TrialID=ISRCTN11647292,E3 , | Excluded by title |
| 392 | Isrctn(2023); Non-surgical treatment of inflammation of the soft tissues around a dental implant. https://trialsearch.who.int/Trial2.aspx?TrialID=ISRCTN94266769,E3 , | Excluded by title |
| 393 | Isrctn(2023); Treatment of peri-implantitis with allografts and enamel proteins. https://trialsearch.who.int/Trial2.aspx?TrialID=ISRCTN15000657,E3 , | Excluded by title |
| 394 | Isrctn(2024); Evaluation of tissue conditioner-assisted complete denture restoration. https://trialsearch.who.int/Trial2.aspx?TrialID=ISRCTN95433057,E3 , | Excluded by title |
| 395 | Isrctn(2024); How soft tissue augmentation after tooth extraction improves implant health: findings from a clinical trial. https://trialsearch.who.int/Trial2.aspx?TrialID=ISRCTN18692174,E3 , | Excluded by title |
| 396 | Iwa≈Ñczyk, B. and Wychowa≈Ñski, P. and Minkiewicz-Zochniak, A. and Strom, K. and Jarzynka, S. and Oledzka, G.(2020); Bioactive healing abutment as a potential tool for the treatment of peri-implant disease-in vitro study. Applied Sciences (Switzerland),E3 ,15 | Excluded by title |
| 397 | J. A. Kopman, D. M. Kim, S. S. Rahman, J. A. Arandia, N. Y. Karimbux and J. P. Fiorellini. Modulating the effects of diabetes on osseointegration with aminoguanidine and doxycycline. 2005. | Excluded by title |
| 398 | J. A. Shibli, L. A. Cardoso, T. Onuma, D. Ferrari, A. Blay and E. Marcantonio. Er,Cr: YSGG and systemic antibiotic in the surgical treatment of peri-implantitis. 2012. | Excluded by title |
| 399 | J. A. Shibli, M. C. Martins, L. H. Theodoro, R. F. Lotufo, V. G. Garcia and E. J. Marcantonio. Lethal photosensitization in microbiological treatment of ligature-induced peri-implantitis: a preliminary study in dogs. 2003. | Excluded by title |
| 400 | J. A. Waasdorp, C. I. Evian and M. Mandracchia. Immediate Placement of Implants Into Infected Sites: A Systematic Review of the Literature. 2010. | Excluded by title |
| 401 | J. Ata-Ali, F. Ata-Ali and P. Galindo-Moreno. Treatment of periimplant mucositis: a systematic review of randomized controlled trials. 2015. | Excluded by title |
| 402 | J. Ata-Ali, F. Ata-Ali, N. Di-Benedetto, L. Bagán and J.-V. Bagán. Does HIV infection have an impact upon dental implant osseointegration? A systematic review. 2015. | Excluded by title |
| 403 | J. B. Park, G. Lee, B. G. Yun, C. H. Kim and Y. Ko. Comparative effects of chlorhexidine and essential oils containing mouth rinse on stem cells cultured on a titanium surface. 2014. | Excluded by title |
| 404 | J. B. Park. Treatment of peri-implantitis with deproteinised bovine bone and tetracycline: a case report. 2012. | Excluded by title |
| 405 | J. Boateng and O. Catanzano. Advanced Therapeutic Dressings for Effective Wound Healing - A Review. 2015. | Excluded by title |
| 406 | J. Caballé-Serrano, Y. Abdeslam-Mohamed, A. Munar-Frau, M. Fujioka-Kobayashi, F. Hernández-Alfaro and R. Miron. Adsorption and release kinetics of growth factors on barrier membranes for guided tissue/bone regeneration: A systematic review. 2019. | Excluded by title |
| 407 | J. Cosyn, A. Eghbali, L. Hanselaer, T. De Rouck, I. Wyn, M. M. Sabzevar, R. Cleymaet and H. De Bruyn. Four modalities of single implant treatment in the anterior maxilla: A clinical, radiographic, and aesthetic evaluation. 2013. | Excluded by title |
| 408 | J. D. Da Silva, J. Kazimiroff, A. Papas, F. A. Curro, V. P. Thompson, D. A. Vena, H. Wu, D. Collie and R. G. Craig. Outcomes of implants and restorations placed in general dental practices: a retrospective study by the Practitioners Engaged in Applied Research and Learning (PEARL) Network. 2014. | Excluded by title |
| 409 | J. D. Devoto. Nuevos conceptos sobre cicatrización periodontal y peri-implantaria. 1997. | Excluded by title |
| 410 | J. D. Kretlow, P. P. Spicer, J. A. Jansen, C. A. Vacanti, F. K. Kasper and A. G. Mikos. Uncultured marrow mononuclear cells delivered within fibrin glue hydrogels to porous scaffolds enhance bone regeneration within critical-sized rat cranial defects. 2010. | Excluded by title |
| 411 | J. Dhillon, S. A. Young, S. E. Sherman, G. I. Bell, B. G. Amsden, D. A. Hess and L. E. Flynn. Peptide-modified methacrylated glycol chitosan hydrogels as a cell-viability supporting pro-angiogenic cell delivery platform for human adipose-derived stem/stromal cells. 2019. | Excluded by title |
| 412 | J. E. M. Franco, S. Cai, L. A. P. A. d. Lima, A. S. Gonçalves and T. N. d. Campos. Avaliação da capacidade de descontaminação da terapia fotodinâmica no pós-operatório imediato de implantes: estudo piloto. 2010. | Excluded by title |
| 413 | J. Emrani, W. Chee and J. Slots. Bacterial colonization of oral implants from nondental sources. 2009. | Excluded by title |
| 414 | J. F. Requicha, C. A. Viegas, F. Muñoz, R. L. Reis and M. E. Gomes. Periodontal tissue engineering strategies based on nonoral stem cells. 2014. | Excluded by title |
| 415 | J. Gallo, M. Holinka and C. S. Moucha. Antibacterial surface treatment for orthopaedic implants. 2014. | Excluded by title |
| 416 | J. H. Fu, J. D. Bashutski, K. Al-Hezaimi and H. L. Wang. Statins, Glucocorticoids, and Nonsteroidal Anti-Inflammatory Drugs: Their Influence on Implant Healing. 2012. | Excluded by title |
| 417 | J. H. Lee, M. Y. Ryu, H. R. Baek, J. H. Seo, K. M. Lee and J. H. Lee. Generation of an rhBMP-2-loaded beta-tricalcium phosphate/hydrogel composite and evaluation of its efficacy on peri-implant bone formation. 2014. | Excluded by title |
| 418 | J. J. Shi, Z. B. Luo and W. X. Chen. Bone morphogenetic protein-4 compounded with platelet-rich plasma promotes bone healing. 2014. | Excluded by title |
| 419 | J. J. Suh, Z. Simon, Y. S. Jeon, B. G. Choi and C. K. Kim. The use of implantoplasty and guided bone regeneration in the treatment of peri-implantitis: two case reports. 2003. | Excluded by title |
| 420 | J. Jofre, D. Valenzuela, P. Quintana and C. Asenjo-Lobos. Protocol for immediate implant replacement of infected teeth. 2012. | Excluded by title |
| 421 | J. K. Cha, U. W. Jung, D. S. Thoma, C. H. F. Hammerle and R. E. Jung. Osteogenic efficacy of BMP-2 mixed with hydrogel and bone substitute in peri-implant dehiscence defects in dogs: 16 weeks of healing. 2018. | Excluded by title |
| 422 | J. L. Gulinelli, P. L. d. Santos, R. O. d. Moraes, L. S. A. Osorio, T. Calcagnotto, R. A. G. Senko, A. F. B. Condezo and G. A. H. Kudo. Peri-implantite: tratamentos e manutenção peri-implantar parte 2*. 2016. | Excluded by title |
| 423 | J. L. Roberts, S. Khan, C. Emanuel, L. C. Powell, M. F. Pritchard, E. Onsoyen, R. Myrvold, D. W. Thomas and K. E. Hill. An in vitro study of alginate oligomer therapies on oral biofilms. 2013. | Excluded by title |
| 424 | J. Lee, E. N. Lee, J. Yoon, S. M. Chung, H. Prasad, C. Susin and U. M. Wikesjo. Comparative study of Chinese hamster ovary cell versus Escherichia coli-derived bone morphogenetic protein-2 using the critical-size supraalveolar peri-implant defect model. 2013. | Excluded by title |
| 425 | J. Lindhe and J. Meyle. Peri-implant diseases: Consensus Report of the Sixth European Workshop on Periodontology. 2008. | Excluded by title |
| 426 | J. Lorenz, T. Korzinskas, P. Chia, S. A. Maawi, K. Eichler, R. A. Sader and S. Ghanaati. Do Clinical and Radiological Assessments Contribute to the Understanding of Biomaterials? Results From a Prospective Randomized Sinus Augmentation Split-Mouth Trial. 2018. | Excluded by title |
| 427 | J. Lyczek, B. Kawala and J. Antoszewska-Smith. Influence of antibiotic prophylaxis on the stability of orthodontic microimplants: A pilot randomized controlled trial. 2018. | Excluded by title |
| 428 | J. M. de Almeida, H. R. Matheus, D. J. Rodrigues Gusman, P. L. Faleiros, N. Januario de Araujo and V. C. Noronha Novaes. Effectiveness of Mechanical Debridement Combined With Adjunctive Therapies for Nonsurgical Treatment of Periimplantitis: A Systematic Review. 2017. | Excluded by title |
| 429 | J. M. Ray and R. G. Triplett. What is the Role of Biofilms in Severe Head and Neck Infections?. 2011. | Excluded by title |
| 430 | J. M. Stein, C. Hammacher and S. S. Michael. Combination of ultrasonic decontamination, soft tissue curettage, and submucosal air polishing with povidone-iodine application for non-surgical therapy of peri-implantitis: 12 Month clinical outcomes. 2017. | Excluded by title |
| 431 | J. M. Stein, C. Hammacher and S. S. Y. Michael. Combination of ultrasonic decontamination, soft tissue curettage, and submucosal air polishing with povidone-iodine application for non-surgical therapy of peri-implantitis: 12-month clinical outcomes. 2018. | Excluded by title |
| 432 | J. Meyle. Mechanical, chemical and laser treatments of the implant surface in the presence of marginal bone loss around implants. 2012. | Excluded by title |
| 433 | J. Mir-Mari, H. Wui, R. E. Jung, C. H. Hammerle and G. I. Benic. Influence of blinded wound closure on the volume stability of different GBR materials: an in vitro cone-beam computed tomographic examination. 2016. | Excluded by title |
| 434 | J. Moradian-Oldak, H. B. Wen, G. B. Schneider and C. M. Stanford. Tissue engineering strategies for the future generation of dental implants. 2006. | Excluded by title |
| 435 | J. P. Albouy, I. Abrahamsson, L. G. Persson and T. Berglundh. Implant surface characteristics influence the outcome of treatment of peri-implantitis: an experimental study in dogs. 2011. | Excluded by title |
| 436 | J. S. Fernandez-Moure, J. L. Van Eps, F. J. Cabrera, Z. Barbosa, G. Medrano del Rosal, B. K. Weiner, W. A. Ellsworth and E. Tasciotti. Platelet-rich plasma: a biomimetic approach to enhancement of surgical wound healing. 2017. | Excluded by title |
| 437 | J. S. Van Epps and J. G. Younger. Implantable device-related infection. 2016. | Excluded by title |
| 438 | J. Shao, E. Kolwijck, J. A. Jansen, F. Yang and X. F. Walboomers. Animal models for percutaneous-device-related infections: a review. 2017. | Excluded by title |
| 439 | J. Sjollema, S. A. J. Zaat, V. Fontaine, M. Ramstedt, R. Luginbuehl, K. Thevissen, J. Li, H. C. van der Mei and H. J. Busscher. In vitro methods for the evaluation of antimicrobial surface designs. 2018. | Excluded by title |
| 440 | J. Slots. Periodontitis: facts, fallacies and the future. 2017. | Excluded by title |
| 441 | J. T. Mellonig and M. Nevins. Guided bone regeneration of bone defects associated with implants: An evidence-based outcome assessment. 1995. | Excluded by title |
| 442 | J. Takebe, K. Miyata, S. Miura and S. Ito. Effects of the nanotopographic surface structure of commercially pure titanium following anodization-hydrothermal treatment on gene expression and adhesion in gingival epithelial cells. 2014. | Excluded by title |
| 443 | J. Torres García-Denche, X. Wu, P. P. Martinez, H. Eimar, D. J. A. Ikbal, G. Hernández, E. Lõpez-Cabarcos, I. Fernandez-Tresguerres and F. Tamimi. Membranes over the lateral window in sinus augmentation procedures: A two-arm and split-mouth randomized clinical trials. 2013. | Excluded by title |
| 444 | J. Vilarrasa, L. M. Delgado, M. Galofre, G. Alvarez, D. Violant, J. M. Manero, V. Blanc, F. J. Gil and J. Nart. In vitro evaluation of a multispecies oral biofilm over antibacterial coated titanium surfaces. 2018. | Excluded by title |
| 445 | J. W. Park, S. G. Lee, B. J. Choi and J. Y. Suh. Effects of a cell adhesion molecule coating on the blasted surface of titanium implants on bone healing in the rabbit femur. 2007. | Excluded by title |
| 446 | J. W. Park, S. H. Han and T. Hanawa. Effects of Surface Nanotopography and Calcium Chemistry of Titanium Bone Implants on Early Blood Platelet and Macrophage Cell Function. 2018. | Excluded by title |
| 447 | J. Waasdorp and M. Reynolds. Nonsurgical treatment of retrograde peri-implantitis: a case report. 2010. | Excluded by title |
| 448 | J. Y. Hong, J. Yon, J. S. Lee, I. K. Lee, C. Yang, M. S. Kim, S. H. Choi and U. W. Jung. Effects of epigallocatechin-3-gallate on the healing of extraction sockets with a periapical lesion: A pilot study in dogs. 2015. | Excluded by title |
| 449 | J. Y. Huang, H. Q. Li, L. Chen, H. M. Zhao and Y. Lin. [Medication compliance and diet compliance in 309 oral lichen planus patients]. 2016. | Excluded by title |
| 450 | J. Y. Li, X. J. Wang, L. N. Wang, X. X. Ying, X. Ren, H. Y. Liu, L. Xu and G. W. Ma. High In Vitro Antibacterial Activity of Pac-525 against Porphyromonas gingivalis Biofilms Cultured on Titanium. 2015. | Excluded by title |
| 451 | Jerv√∏e-Storm, P. M. and Bunke, J. and Worthington, H. V. and Needleman, I. and Cosgarea, R. and MacDonald, L. and Walsh, T. and Lewis, S. R. and Jepsen, S.(2024); Adjunctive antimicrobial photodynamic therapy for treating periodontal and peri-implant diseases. Cochrane Database Syst Rev,E3 ,7Cd011778 | Excluded by title |
| 452 | Jerv√∏e-Storm, P. M. and Jepsen, S. and Worthington, H. V. and Needleman, I. and Eberhard, J.(2015); Adjunctive antimicrobial photodynamic therapy for treating periodontal and peri-implant diseases. Cochrane Database of Systematic Reviews,E3 ,6 | Excluded by title |
| 453 | Juarez, C. and Langa, L. and Mendoza, R. and Guerrero, M. E. and Oliva, J. and Mayta-Tovalino, F.(2023); Antimicrobial photodynamic therapy for the treatment of peri-implantitis: A literature review. Journal of International Society of Preventive and Community Dentistry,E3 ,283-88 | Excluded by title |
| 454 | K. A. Schlegel, C. Prechtl, T. Most, C. Seidl, R. Lutz and C. von Wilmowsky. Osseointegration of SLActive implants in diabetic pigs. 2013. | Excluded by title |
| 455 | K. Dzobo, N. E. Thomford, D. A. Senthebane, H. Shipanga, A. Rowe, C. Dandara, M. Pillay and K. S. C. M. Motaung. Advances in regenerative medicine and tissue engineering: Innovation and transformation of medicine. 2018. | Excluded by title |
| 456 | K. E. Schmidt, T. M. Auschill, C. Heumann, R. Frankenberger, S. Eick, A. Sculean and N. B. Arweiler. Clinical and laboratory evaluation of the effects of different treatment modalities on titanium healing caps: a randomized, controlled clinical trial. 2018. | Excluded by title |
| 457 | K. Gomi, Y. Matsushima, Y. Ujiie, S. Shirakawa, T. Nagano, M. Kanazashi and A. Yashima. Full-mouth scaling and root planing combined with azithromycin to treat peri-implantitis. 2015. | Excluded by title |
| 458 | K. Ito, K. Takahashi, T. Eda, T. Kondoh and A. Goss. Peri-implant squamous cell carcinoma. 2018. | Excluded by title |
| 459 | K. J. Jeong and D. S. Kohane. Surface modification and drug delivery for biointegration. 2011. | Excluded by title |
| 460 | K. J. Kwon and H. Seok. Silk protein-based membrane for guided bone regeneration. 2018. | Excluded by title |
| 461 | K. M. Ainslie, E. M. Bachelder, S. Borkar, A. S. Zahr, A. Sen, J. V. Badding and M. V. Pishko. Cell adhesion on nanofibrous polytetrafluoroethylene (nPTFE). 2007. | Excluded by title |
| 462 | K. M. Fawzy El-Sayed and C. E. Dörfer. Animal Models for Periodontal Tissue Engineering: A Knowledge-Generating Process. 2017. | Excluded by title |
| 463 | K. Mizutani, A. Aoki, D. Coluzzi, R. Yukna, C. Y. Wang, V. Pavlic and Y. Izumi. Lasers in minimally invasive periodontal and peri-implant therapy. 2016. | Excluded by title |
| 464 | K. N. Leknes, J. Yang, M. Qahash, G. Polimeni, C. Susin and U. M. E. Wikesjö. Alveolar ridge augmentation using implants coated with recombinant human bone morphogenetic protein-7 (rhBMP-7rhOP-1): Radiographic observations. 2008. | Excluded by title |
| 465 | K. Nickles, B. Schacher, P. Ratka-Kruger, M. Krebs and P. Eickholz. Long-term results after treatment of periodontitis in patients with Papillon-Lefevre syndrome: success and failure. 2013. | Excluded by title |
| 466 | K. Pałka and R. Pokrowiecki. Porous Titanium Implants: A Review. 2018. | Excluded by title |
| 467 | K. Prasad, O. Bazaka, M. Chua, M. Rochford, L. Fedrick, J. Spoor, R. Symes, M. Tieppo, C. Collins, A. Cao, D. Markwell, K. Ostrikov and K. Bazaka. Metallic biomaterials: Current challenges and opportunities. 2017. | Excluded by title |
| 468 | K. R. Nagaraj. Use of lasers in prosthodontics: A review. 2012. | Excluded by title |
| 469 | K. Shimono, M. Oshima, H. Arakawa, A. Kimura, K. Nawachi and T. Kuboki. The effect of growth factors for bone augmentation to enable dental implant placement: A systematic review. 2010. | Excluded by title |
| 470 | K. Sinjab, C. Garaicoa-Pazmino and H. L. Wang. Decision Making for Management of Periimplant Diseases. 2018. | Excluded by title |
| 471 | K. W. H. Lo, B. D. Ulery, M. Deng, K. M. Ashe and C. T. Laurencin. Current patents on osteoinductive molecules for bone tissue engineering. 2011. | Excluded by title |
| 472 | K. Yuan, K. C. Chen, Y. J. Chan, C. C. Tsai, H. H. Chen and C. C. Shih. Dental implant failure associated with bacterial infection and long-term bisphosphonate usage: a case report. 2012. | Excluded by title |
| 473 | K. Zhao, F. Wang, W. Huang, X. Wang and Y. Wu. Comparison of Dental Implant Performance Following Vertical Alveolar Bone Augmentation With Alveolar Distraction Osteogenesis or Autogenous Onlay Bone Grafts: A Retrospective Cohort Study. 2017. | Excluded by title |
| 474 | Kadkhodazadeh, M. and Amid, R. and Amirinasab, O. and Amirbandeh, O. and Moscowchi, A.(2024); Risk Indicators of Peri-Implant Diseases in Public and Private Clinics: A Multicenter Study. International Journal of Dentistry,E3 , | Excluded by title |
| 475 | Kadkhodazadeh, M. and Haririan, H. and Amid, R. and Rezaei, F. and Yazdani, A. and Baghban, A. A. and Azadi, A.(2024); An Analysis of Scientific Research Trends in Oral Implantology Between 2016 and 2022. Journal of Oral Implantology,E3 ,4322-327 | Excluded by title |
| 476 | Kang, P. and Sanz-Miralles, E. and Li, J. and Linden, E. and Momen-Heravi, F.(2023); Efficacy of Er,Cr:YSGG Laser Application in Nonsurgical Treatment of Peri-implantitis: A Human Randomized Controlled Trial. International Journal of Periodontics & Restorative Dentistry,E3 ,1E1-E9 | Excluded by title |
| 477 | Karande, V. and Ranade, S. and Karande, P. and Bagchi, P.(2024); Comparative evaluation of buccal pad of fat with and without bovine collagen membrane in the management of oral submucous fibrosis: a prospective clinical study. African journal of biological sciences (south africa),E3 ,2025‚Äê2029 | Excluded by title |
| 478 | Karimi, M. R. and Hasani, A. and Khosroshahian, S.(2016); Efficacy of antimicrobial photodynamic therapy as an adjunctive to mechanical debridement in the treatment of peri-implant diseases: A randomized controlled clinical trial. Journal of Lasers in Medical Sciences,E3 ,3139-145 | Excluded by title |
| 479 | Kauark-Fontes, E. and Migliorati, C. A. and Epstein, J. B. and Bensadoun, R. J. and Gueiros, L. A. M. and Carroll, J. and Ramalho, L. M. P. and Santos-Silva, A. R.(2023); Twenty-year analysis of photobiomodulation clinical studies for oral mucositis: a scoping review. Oral Surgery Oral Medicine Oral Pathology Oral Radiology,E3 ,5626-641 | Excluded by title |
| 480 | Kct(2024); Clinical study comparing short-diameter implants with 3.5 mm diameter to long-diameter implants with 5.0 mm diameter for implant placement. https://trialsearch.who.int/Trial2.aspx?TrialID=KCT0009415,E3 , | Excluded by title |
| 481 | Khalifah, M. A. and Elgendy, A. M. A. and Elgendy, E.(2023); Untreated Mineralized Dentin Graft Versus Xenograft Around Immediately Placed Dental Implants in the Mandibular Anterior Region: a Randomized Controlled Clinical Trial. International journal of oral & maxillofacial implants,E3 ,1‚Äê29 | Excluded by title |
| 482 | Kikuchi, T. and Wada, M. and Mameno, T. and Hasegawa, D. and Serino, G. and Ikebe, K.(2022); Longitudinal study on the effect of keratinized mucosal augmentation surrounding dental implants in preventing peri-implant bone loss. PeerJ,E3 , | Excluded by title |
| 483 | Kohal, R. J. and Vach, K. and Butz, F. and Spies, B. C. and Patzelt, S. B. M. and Burkhardt, F.(2023); One-Piece Zirconia Oral Implants for the Support of Three-Unit Fixed Dental Prostheses: Three-Year Results from a Prospective Case Series. Journal of Functional Biomaterials,E3 ,1 | Excluded by title |
| 484 | Kontogiannopoulos, K. N. and Kapourani, A. and Gkougkourelas, I. and Anagnostaki, M. E. and Tsalikis, L. and Assimopoulou, A. N. and Barmpalexis, P.(2023); A Review of the Role of Natural Products as Treatment Approaches for Xerostomia. Pharmaceuticals,E3 ,826 | Excluded by title |
| 485 | Koukos, G. and Papadopoulos, C. and Tsalikis, L. and Sakellari, D. and Arsenakis, M. and Konstantinidis, A.(2014); Prevalence of antibiotic resistance genes in subjects with successful and failing dental implants. A pilot study. Open Dentistry Journal,E3 ,1257-263 | Excluded by title |
| 486 | Krennmair, G. and Weinl√§nder, M. and Forstner, T. and Krennmair, S. and Stimmelmayr, M. and Malek, M.(2023); Transsinusoidal lateral nasal floor augmentation for implant placement in the atrophic premaxilla: A within-patient, 5-year, prospective comparative study. Clin Oral Implants Res,E3 ,8822-838 | Excluded by title |
| 487 | Kreve, S. and Pinheiro De Carvalho, G. and Ramos, E. V. and Dias, S. C.(2016); Clinical Evaluation of Hygiene Maintenance of Full-arch Implant-supported Prostheses. Journal of International Oral Health,E3 ,9903-910 | Excluded by title |
| 488 | ksffhm, R. B. R.(2023); Evaluation of Irradiation over the radial artery with Low-Intensity Laser as a method for controlling Oral Mucositis in patients undergoing Radiotherapy. https://trialsearch.who.int/Trial2.aspx?TrialID=RBR-8ksffhm,E3 , | Excluded by title |
| 489 | L. C. Zhang and L. Y. Chen. A Review on Biomedical Titanium Alloys: Recent Progress and Prospect. 2019. | Excluded by title |
| 490 | L. Canullo, J. F. Dehner, D. Penarrocha, V. Checchi, A. Mazzoni and L. Breschi. Soft Tissue Response to Titanium Abutments with Different Surface Treatment: Preliminary Histologic Report of a Randomized Controlled Trial. 2016. | Excluded by title |
| 491 | L. Canullo, T. Genova, H. L. Wang, S. Carossa and F. Mussano. Plasma of Argon Increases Cell Attachment and Bacterial Decontamination on Different Implant Surfaces. 2017. | Excluded by title |
| 492 | L. Chambrone, H. L. Wang and G. E. Romanos. Antimicrobial photodynamic therapy for the treatment of periodontitis and peri-implantitis: An American Academy of Periodontology best evidence review. 2018. | Excluded by title |
| 493 | L. Ding, P. Zhang, X. Wang and S. Kasugai. A doxycycline-treated hydroxyapatite implant surface attenuates the progression of peri-implantitis: A radiographic and histological study in mice. 2019. | Excluded by title |
| 494 | L. Drago, M. Bortolin, S. Taschieri, E. De Vecchi, S. Agrappi, M. Del Fabbro, L. Francetti and R. Mattina. Erythritol/chlorhexidine combination reduces microbial biofilm and prevents its formation on titanium surfaces in vitro. 2017. | Excluded by title |
| 495 | L. Drago, M. Del Fabbro, M. Bortolin, C. Vassena, E. De Vecchi and S. Taschieri. Biofilm removal and antimicrobial activity of two different air-polishing powders: an in vitro study. 2014. | Excluded by title |
| 496 | L. Feller, Y. Jadwat, R. Chandran, I. Lager, M. Altini and J. Lemmer. Radiolucent Inflammatory Implant Periapical Lesions: A Review of the Literature. 2014. | Excluded by title |
| 497 | L. G. Persson, I. Ericsson, T. Berglundh and J. Lindhe. Guided bone regeneration in the treatment of periimplantitis. 1996. | Excluded by title |
| 498 | L. G. Persson, M. G. Araujo, T. Berglundh, K. Grondahl and J. Lindhe. Resolution of peri-implantitis following treatment. An experimental study in the dog. 1999. | Excluded by title |
| 499 | L. G. Persson, T. Berglundh, J. Lindhe and L. Sennerby. Re-osseointegration after treatment of peri-implantitis at different implant surfaces. An experimental study in the dog. 2001. | Excluded by title |
| 500 | L. G. Persson, T. Berglundh, L. Sennerby and A. Lindhe. Re-osseointegration after treatment of peri-implantitis at different implant surfaces - An experimental study in the dog. 2001. | Excluded by title |
| 501 | L. Gu, Q. Wang and Y. C. Yu. Eleven dental implants placed in a liver transplantation patient: a case report and 5-year clinical evaluation. 2011. | Excluded by title |
| 502 | L. H. Silverstein, J. P. Koch, M. D. Lefkove, J. J. Garnick, B. Singh and D. E. Steflik. Nifedipine-induced gingival enlargement around dental implants: a clinical report. 1995. | Excluded by title |
| 503 | L. J. A. Heitz-Mayfield and G. E. Salvi. Peri-implant mucositis. 2018. | Excluded by title |
| 504 | L. J. Heitz-Mayfield and N. P. Lang. Antimicrobial treatment of peri-implant diseases. 2004. | Excluded by title |
| 505 | L. J. Heitz-Mayfield, G. E. Salvi, D. Botticelli, A. Mombelli, M. Faddy and N. P. Lang. Anti-infective treatment of peri-implant mucositis: a randomised controlled clinical trial. 2011. | Excluded by title |
| 506 | L. J. Ling. Current concept of guided tissue regeneration with non-absorbable membrane. 1998. | Excluded by title |
| 507 | L. J. Tavares, A. C. Pavarina, C. E. Vergani and E. D. de Avila. The impact of antimicrobial photodynamic therapy on peri-implant disease: What mechanisms are involved in this novel treatment?. 2017. | Excluded by title |
| 508 | L. K. Cheung and A. C. F. Leung. Dental Implants in Reconstructed Jaws: Implant Longevity and Peri-Implant Tissue Outcomes. 2003. | Excluded by title |
| 509 | L. Karygianni, S. Ruf, M. Follo, E. Hellwig, M. Bucher, A. C. Anderson, K. Vach and A. Al-Ahmad. Novel Broad-Spectrum Antimicrobial Photoinactivation of In Situ Oral Biofilms by Visible Light plus Water-Filtered Infrared A. 2014. | Excluded by title |
| 510 | L. Larsson, A. M. Decker, L. Nibali, S. P. Pilipchuk, T. Berglundh and W. V. Giannobile. Regenerative Medicine for Periodontal and Peri-implant Diseases. 2016. | Excluded by title |
| 511 | L. Lin, Y. Fang, Y. Liao, G. Chen, C. Gao and P. Zhu. 3D Printing and Digital Processing Techniques in Dentistry: A Review of Literature. 2019. | Excluded by title |
| 512 | L. Malchiodi, A. Cucchi, P. Ghensi, D. Consonni and P. F. Nocini. Influence of crown-implant ratio on implant success rates and crestal bone levels: a 36-month follow-up prospective study. 2014. | Excluded by title |
| 513 | L. Nastri, A. De Rosa, V. De Gregorio, V. Grassia and G. Donnarumma. A New Controlled-Release Material Containing Metronidazole and Doxycycline for the Treatment of Periodontal and Peri-Implant Diseases: Formulation and In Vitro Testing. 2019. | Excluded by title |
| 514 | L. Nibali and N. Donos. Radiographic bone fill of peri-implantitis defects following nonsurgical therapy: report of three cases. 2011. | Excluded by title |
| 515 | L. P. Faverani, T. O. B. Polo, G. Ramalho-Ferreira, G. A. C. Momesso, J. S. Hassumi, A. C. Rossi, A. R. Freire, F. B. Prado, E. R. Luvizuto, R. Gruber and R. Okamoto. Raloxifene but not alendronate can compensate the impaired osseointegration in osteoporotic rats. 2018. | Excluded by title |
| 516 | L. P. Palin, T. O. B. Polo, F. R. S. Batista, P. H. S. Gomes-Ferreira, I. R. Garcia Junior, A. C. Rossi, A. Freire, L. P. Faverani, D. H. Sumida and R. Okamoto. Daily melatonin administration improves osseointegration in pinealectomized rats. 2018. | Excluded by title |
| 517 | L. Pacifici, F. De Angelis, A. Orefici and A. Cielo. Metals used in maxillofacial surgery. 2016. | Excluded by title |
| 518 | L. Polo-Corrales, M. Latorre-Esteves and J. E. Ramirez-Vick. Scaffold design for bone regeneration. 2014. | Excluded by title |
| 519 | L. Sbordone and C. Bortolaia. Oral microbial biofilms and plaque-related diseases: microbial communities and their role in the shift from oral health to disease. 2003. | Excluded by title |
| 520 | L. Sbordone, A. Barone, L. Ramaglia, R. N. Ciaglia and V. J. Iacono. Antimicrobial susceptibility of periodontopathic bacteria associated with failing implants. 1995. | Excluded by title |
| 521 | L. Trombelli and R. Farina. Efficacy of triclosan-based toothpastes in the prevention and treatment of plaque-induced periodontal and peri-implant diseases. 2013. | Excluded by title |
| 522 | L. Xiang, L. Ma, N. Wei, T. Wang, Q. Yao, B. Yang, Y. Xiong, Y. Wu and P. Gong. Effect of lentiviral vector overexpression alpha-calcitonin gene-related peptide on titanium implant osseointegration in alpha-CGRP-deficient mice. 2017. | Excluded by title |
| 523 | L. Xu, Y. Wang, V. T. Nguyen and J. Chen. Effects of Topical Antibiotic Prophylaxis on Wound Healing After Flapless Implant Surgery: A Pilot Study. 2016. | Excluded by title |
| 524 | L. Yin, S. Yang, M. He, Y. Chang, K. Wang, Y. Zhu, Y. Liu, Y. Chang and Z. Yu. Physicochemical and biological characteristics of BMP-2/IGF-1-loaded three-dimensional coaxial electrospun fibrous membranes for bone defect repair. 2017. | Excluded by title |
| 525 | Laheij, Amga and Dillen, L. M. and Nur, E. and Raber-Durlacher, J. E.(2024); Self-perceived oral health in hemato-oncological patients and the relation to quality of life. Supportive Care in Cancer,E3 ,1012 | Excluded by title |
| 526 | Lazar, L. and Dak√≥, T. and Mure?an, I√â and Suciu, M. and Maftei, G. A. and Tatarciuc, M. and Lazar, A. P.(2023); Is Laser Therapy an Adjuvant in the Treatment of Peri-Implant Mucositis? A Randomized Clinical Trial. Diagnostics (Basel, Switzerland),E3 ,6 | Excluded by title |
| 527 | Lazar, L. and Dak√≥, T. and Muresan, I. E. and Suciu, M. and Maftei, G. A. and Tatarciuc, M. and Lazar, A. P.(2023); Is Laser Therapy an Adjuvant in the Treatment of Peri-Implant Mucositis? A Randomized Clinical Trial. Diagnostics,E3 ,612 | Excluded by title |
| 528 | Lee, J. and Lim, J. and Lee, J. and Kim, S. and Koo, K. T. and Seol, Y. J. and Ku, Y. and Lee, Y. M. and Rhyu, I. C.(2015); Efficacy of sonic-powered toothbrushes for plaque removal in patients with peri-implant mucositis. Journal of Periodontal and Implant Science,E3 ,256-61 | Excluded by title |
| 529 | Lee, J. H. and Lee, J. B. and Kim, M. Y. and Yoon, J. H. and Choi, S. H. and Kim, Y. T.(2016); Mechanical and biological complication rates of the modified lateral-screw-retained implant prosthesis in the posterior region: An alternative to the conventional Implant prosthetic system. Journal of Advanced Prosthodontics,E3 ,2150-157 | Excluded by title |
| 530 | Lessa, A. D. N. and Celestino, M. D. and Ferreira, J. M. and Lima, I. V. and Ramos, Y. C. S. and Vieira, F. F. and Amancio, Amtd and Caldeira, P. C. and Sousa, S. F. D. and de Aguiar, M. C. F.(2023); Antimicrobial photodynamic therapy for the treatment of oral mucositis-A comparative study. Photodiagnosis and Photodynamic Therapy,E3 ,7 | Excluded by title |
| 531 | Leung, T. J. T. and Nijland, N. and Gerdes, V. E. A. and Loos, B. G.(2022); Prevalence of Periodontal Disease among Patients at the Outpatient Clinic of Internal Medicine in an Academic Hospital in The Netherlands: A Cross-Sectional Pilot Study. Journal of Clinical Medicine,E3 ,20 | Excluded by title |
| 532 | Levin, L. and Schwartz-Arad, D.(2010); Dental implants - Quo vadis?. Journal of Osseointegration,E3 ,134-36 | Excluded by title |
| 533 | Li, H. and Wang, Q. and Xu, W. and Zhao, B.(2024); Efficacy evaluation of endoscopic-assisted subgingival scaling combined with erythritol subgingival sandblasting in treatment of peri-implantitis. Journal of jilin university medicine edition,E3 ,2465‚Äê472 | Excluded by title |
| 534 | Li, J. and Jin, F. and Wang, R. and Shang, X. and Yang, P. and Zhu, Y. and Tsoi, J. K. H. and Chan, K. and Wang, S.(2023); Guided Bone Regeneration in a Periodontally Compromised Individual with Autogenous Tooth Bone Graft: A Radiomics Analysis. Journal of Functional Biomaterials,E3 ,4 | Excluded by title |
| 535 | Lin, C. J. and Tsai, M. H. and Wu, Y. L. and Lung, H. and Chen, H. S. and Wu, A. Y. J.(2022); The Effect of an Er, Cr: YSGG Laser Combined with Implantoplasty Treatment on Implant Surface Roughness and Morphologic Analysis: A Pilot In Vitro Study. Journal of Functional Biomaterials,E3 ,3 | Excluded by title |
| 536 | Lodolo, M. and Thanasuwat, B. and Veluppillai, P. and Bassani, G. and Villa, A.(2023); Dexamethasone solution and dexamethasone in Mucolox‚Ñ¢ for the treatment of oral inflammatory ulcerative diseases: a phase II randomized clinical trial. Journal of oral pathology & medicine,E3 ,9860‚Äê866 | Excluded by title |
| 537 | Luengo, F. and Sanz-Esporr√≠n, J. and Sanz-S√°nchez, I. and Solonko, M. and Herrera, D. and Sanz, M.(2023); Clinical, microbiological and biochemical impact of a supportive care protocol with an air-polishing device, after surgical treatment of peri-implantitis: Randomized clinical trial. Clinical Oral Implants Research,E3 ,4378-392 | Excluded by title |
| 538 | Luengo, F. and Solonko, M. and Sanz-Esporr√≠n, J. and Sanz-S√°nchez, I. and Herrera, D. and Sanz, M.(2022); Clinical, Microbiological, and Biochemical Impact of the Surgical Treatment of Peri-Implantitis‚ÄîA Prospective Case Series. Journal of Clinical Medicine,E3 ,16 | Excluded by title |
| 539 | M. A. Atieh. Photodynamic therapy as an adjunctive treatment for chronic periodontitis: a meta-analysis. 2010. | Excluded by title |
| 540 | M. A. Bassetti, R. G. Bassetti and D. D. Bosshardt. The alveolar ridge splitting/expansion technique: A systematic review. 2016. | Excluded by title |
| 541 | M. A. Saghiri, A. Asatourian, F. Garcia-Godoy and N. Sheibani. The role of angiogenesis in implant dentistry part I: Review of titanium alloys, surface characteristics and treatments. 2016. | Excluded by title |
| 542 | M. A. Sanchez-Garces and C. Gay-Escoda. Periimplantitis. 2004. | Excluded by title |
| 543 | M. Aimetti, G. M. Mariani, F. Ferrarotti, E. Ercoli, C. C. Liu and F. Romano. Adjunctive efficacy of diode laser in the treatment of peri-implant mucositis with mechanical therapy: A randomized clinical trial. 2019. | Excluded by title |
| 544 | M. Albertini, L. Lopez-Cerero, M. G. O'Sullivan, C. F. Chereguini, S. Ballesta, V. Rios, M. Herrero-Climent and P. Bullon. Assessment of periodontal and opportunistic flora in patients with peri-implantitis. 2015. | Excluded by title |
| 545 | M. Asgari, R. Hang, C. Wang, Z. Yu, Z. Li and Y. Xiao. Biodegradable metallicwires in dental and orthopedic applications: A review. 2018. | Excluded by title |
| 546 | M. Astasov-Frauenhoffer, O. Braissant, I. Hauser-Gerspach, R. Weiger, C. Walter, N. U. Zitzmann and T. Waltimo. Microcalorimetric determination of the effects of amoxicillin, metronidazole, and their combination on in vitro biofilm. 2014. | Excluded by title |
| 547 | M. B. Negroni de Bonvehi and M. d. C. Domenech. Periimplantitis infecciosa: propuesta de un plan preventivo. 1996. | Excluded by title |
| 548 | M. Bassetti, D. Schar, B. Wicki, S. Eick, C. A. Ramseier, N. B. Arweiler, A. Sculean and G. E. Salvi. Anti-infective therapy of peri-implantitis with adjunctive local drug delivery or photodynamic therapy: 12-month outcomes of a randomized controlled clinical trial. 2014. | Excluded by title |
| 549 | M. C. Bottino, D. Pankajakshan and J. E. Nör. Advanced Scaffolds for Dental Pulp and Periodontal Regeneration. 2017. | Excluded by title |
| 550 | M. C. Goiato, A. S. Takamiya, L. M. Alves and D. M. dos Santos. Postsurgical care for rehabilitation with implant-retained extraoral prostheses. 2010. | Excluded by title |
| 551 | M. C. Manz. Factors associated with radiographic vertical bone loss around implants placed in a clinical study. 2000. | Excluded by title |
| 552 | M. C. Sanchez, E. Fernandez, A. Llama-Palacios, E. Figuero, D. Herrera and M. Sanz. Response to antiseptic agents of periodontal pathogens in in vitro biofilms on titanium and zirconium surfaces. 2017. | Excluded by title |
| 553 | M. Chatterjee, K. Hatori, J. Duyck, K. Sasaki, I. Naert and K. Vandamme. High-frequency loading positively impacts titanium implant osseointegration in impaired bone. 2015. | Excluded by title |
| 554 | M. Chevalier, S. Ranque and I. Precheur. Oral fungal-bacterial biofilm models in vitro: a review. 2018. | Excluded by title |
| 555 | M. D. Al Amri, S. V. Kellesarian, A. Ahmed, A. A. Al-Kheraif, G. E. Romanos and F. Javed. Efficacy of periimplant mechanical debridement with and without adjunct antimicrobial photodynamic therapy in patients with type 2 diabetes mellitus. 2016. | Excluded by title |
| 556 | M. d. M. Naves, B. Z. Horbylon, C. d. F. Gomes, H. H. M. d. Menezes, C. Bataglion and D. d. Magalhães. Immediate implants placed into infected sockets: a case report with 3-year follow-up. 2009. | Excluded by title |
| 557 | M. D. R. Vásquez-Segura, S.-L. Gil-Cueva and K. A. Vásquez-Segura. Rehabilitación oral con implantes dentales en paciente con periodontitis crónica. 2015. | Excluded by title |
| 558 | M. Dang, L. Saunders, X. Niu, Y. Fan and P. X. Ma. Biomimetic delivery of signals for bone tissue engineering. 2018. | Excluded by title |
| 559 | M. Del Fabbro, C. Boggian and S. Taschieri. Immediate implant placement into fresh extraction sites with chronic periapical pathologic features combined with plasma rich in growth factors: preliminary results of single-cohort study. 2009. | Excluded by title |
| 560 | M. Du, T. Zhu, X. Duan, S. Ge, N. Li, Q. Sun and P. Yang. Acellular dermal matrix loading with bFGF achieves similar acceleration of bone regeneration to BMP-2 via differential effects on recruitment, proliferation and sustained osteodifferentiation of mesenchymal stem cells. 2017. | Excluded by title |
| 561 | M. E. Fritz, J. Malmquist, D. Koth, M. Jeffcoat, R. Hardwick, L. D. Braswell and J. Lemons. The use of guided bone regeneration to fill large mandibular defects in monkeys: A pilot study. 1994. | Excluded by title |
| 562 | M. Esposito, H. V. Worthington, P. Coulthard and A. Jokstad. Interventions for replacing missing teeth: maintaining and re-establishing healthy tissues around dental implants. 2002. | Excluded by title |
| 563 | M. Esposito, H. V. Worthington, P. Thomsen and P. Coulthard. Interventions for replacing missing teeth: maintaining health around dental implants. 2004. | Excluded by title |
| 564 | M. Esposito, J. M. Hirsch, U. Lekholm and P. Thomsen. Biological factors contributing to failures of osseointegrated oral implants. (II). Etiopathogenesis. 1998. | Excluded by title |
| 565 | M. Esposito, M. G. Grusovin and H. V. Worthington. Interventions for replacing missing teeth: treatment of peri-implantitis. 2012. | Excluded by title |
| 566 | M. Esposito, M. G. Grusovin and H. V. Worthington. Treatment of peri-implantitis: what interventions are effective? A Cochrane systematic review. 2012. | Excluded by title |
| 567 | M. Esposito, M. G. Grusovin, I. Kakisis, P. Coulthard and H. V. Worthington. Interventions for replacing missing teeth: treatment of perimplantitis. 2008. | Excluded by title |
| 568 | M. Esposito, M. G. Grusovin, N. De Angelis, A. Camurati, M. Campailla and P. Felice. The adjunctive use of light-activated disinfection (LAD) with FotoSan is ineffective in the treatment of peri-implantitis: 1-year results from a multicentre pragmatic randomised controlled trial. 2013. | Excluded by title |
| 569 | M. Esposito, M. G. Grusovin, P. Coulthard and H. V. Worthington. The efficacy of interventions to treat peri-implantitis: a Cochrane systematic review of randomised controlled clinical trials. 2008. | Excluded by title |
| 570 | M. F. Sola-Ruiz, J. Del Rio Highsmith, C. Labaig-Rueda and R. Agustin-Panadero. Biologically oriented preparation technique (BOPT) for implant-supported fixed prostheses. 2017. | Excluded by title |
| 571 | M. Faramarzi, Z. Goharfar, R. Pourabbas, A. Kashefimehr and A. Shirmohammadi. Corrigendum: Microbiological and clinical effects of enamel matrix derivative and sustained-release micro-spherical minocycline application as an adjunct to non-surgical therapy in peri-implant mucosal inflammation. 2016. | Excluded by title |
| 572 | M. Faramarzi, Z. Goharfar, R. Pourabbas, A. Kashefimehr and A. Shirmohmmadi. Microbiological and clinical effects of enamel matrix derivative and sustained-release micro-spherical minocycline application as an adjunct to non-surgical therapy in peri-implant mucosal inflammation. 2015. | Excluded by title |
| 573 | M. G. Grusovin, P. Coulthard, E. Jourabchian, H. V. Worthington and M. A. Esposito. Interventions for replacing missing teeth: maintaining and recovering soft tissue health around dental implants. 2008. | Excluded by title |
| 574 | M. G. Patino, M. E. Neiders, S. Andreana, B. Noble and R. E. Cohen. Collagen as an implantable material in medicine and dentistry. 2002. | Excluded by title |
| 575 | M. Ghasemi, A. Etemadi, M. Nedaei, N. Chiniforush and M. Pourhajibagher. Antimicrobial Efficacy of Photodynamic Therapy Using Two Different Light Sources on the Titanium-Adherent Biofilms of Aggregatibacter actinomycetemcomitans: An in vitro study. 2019. | Excluded by title |
| 576 | M. Giannelli, G. Landini, F. Materassi, F. Chellini, A. Antonelli, A. Tani, D. Nosi, S. Zecchi-Orlandini, G. M. Rossolini and D. Bani. Effects of photodynamic laser and violet-blue led irradiation on Staphylococcus aureus biofilm and Escherichia coli lipopolysaccharide attached to moderately rough titanium surface: in vitro study. 2017. | Excluded by title |
| 577 | M. Giannelli, G. Landini, F. Materassi, F. Chellini, A. Antonelli, A. Tani, S. Zecchi-Orlandini, G. M. Rossolini and D. Bani. The effects of diode laser on Staphylococcus aureus biofilm and Escherichia coli lipopolysaccharide adherent to titanium oxide surface of dental implants. An in vitro study. 2016. | Excluded by title |
| 578 | M. Godoy-Gallardo, M. C. Manzanares-Cespedes, P. Sevilla, J. Nart, N. Manzanares, J. M. Manero, F. J. Gil, S. K. Boyd and D. Rodriguez. Evaluation of bone loss in antibacterial coated dental implants: An experimental study in dogs. 2016. | Excluded by title |
| 579 | M. Hallman and A. Thor. Bone substitutes and growth factors as an alternative/complement to autogenous bone for grafting in implant dentistry. 2008. | Excluded by title |
| 580 | M. Hoyos-Nogues, S. Brosel-Oliu, N. Abramova, F. X. Munoz, A. Bratov, C. Mas-Moruno and F. J. Gil. Impedimetric antimicrobial peptide-based sensor for the early detection of periodontopathogenic bacteria. 2016. | Excluded by title |
| 581 | M. Htet, M. Madi, O. Zakaria, T. Miyahara, W. Xin, Z. Lin, K. Aoki and S. Kasugai. Decontamination of Anodized Implant Surface With Different Modalities for Peri-Implantitis Treatment: Lasers and Mechanical Debridement With Citric Acid. 2016. | Excluded by title |
| 582 | M. I. Almagro, J. A. Roman-Blas, M. Bellido, S. Castaneda, R. Cortez and G. Herrero-Beaumont. PTH [1-34] enhances bone response around titanium implants in a rabbit model of osteoporosis. 2013. | Excluded by title |
| 583 | M. Irshad, W. A. van der Reijden, W. Crielaard and M. L. Laine. In vitro invasion and survival of Porphyromonas gingivalis in gingival fibroblasts; role of the capsule. 2012. | Excluded by title |
| 584 | M. J. Lerman, J. Lembong, G. Gillen and J. P. Fisher. 3D printing in cell culture systems and medical applications. 2018. | Excluded by title |
| 585 | M. J. Whitaker, R. A. Quirk, S. M. Howdle and K. M. Shakesheff. Growth factor release from tissue engineering scaffolds. 2001. | Excluded by title |
| 586 | M. Jäger, C. Zilkens, K. Zanger and R. Krauspe. Significance of nano- and microtopography for cell-surface interactions in orthopaedic implants. 2007. | Excluded by title |
| 587 | M. Kaku, Y. Akiba, K. Akiyama, D. Akita and M. Nishimura. Cell-based bone regeneration for alveolar ridge augmentation - Cell source, endogenous cell recruitment and immunomodulatory function. 2015. | Excluded by title |
| 588 | M. M. Querido, L. Aguiar, P. Neves, C. C. Pereira and J. P. Teixeira. Self-disinfecting surfaces and infection control. 2019. | Excluded by title |
| 589 | M. Mahdizade-Ari, M. Pourhajibagher and A. Bahador. Changes of microbial cell survival, metabolic activity, efflux capacity, and quorum sensing ability of Aggregatibacter actinomycetemcomitans due to antimicrobial photodynamic therapy-induced bystander effects. 2019. | Excluded by title |
| 590 | M. Moreno-Sánchez, F. Monje Gil, R. González-García and D. Manzano Solo de Zaldívar. Bifosfonatos e implantes dentales, ¿son incompatibles? Revisión de la literatura. 2016. | Excluded by title |
| 591 | M. Muthukuru, A. Zainvi, E. O. Esplugues and T. F. Flemmig. Non-surgical therapy for the management of peri-implantitis: a systematic review. 2012. | Excluded by title |
| 592 | M. N. Abdallah, Z. Badran, O. Ciobanu, N. Hamdan and F. Tamimi. Strategies for Optimizing the Soft Tissue Seal around Osseointegrated Implants. 2017. | Excluded by title |
| 593 | M. Neuschl, S. T. Becker, E. Behrens and J. Wiltfang. Therapeutic options in the treatment of peri-implant diseases. 2011. | Excluded by title |
| 594 | M. O. Freire, A. Devaraj, A. Young, J. B. Navarro, J. S. Downey, C. Chen, L. O. Bakaletz, H. H. Zadeh and S. D. Goodman. A bacterial-biofilm-induced oral osteolytic infection can be successfully treated by immuno-targeting an extracellular nucleoid-associated protein. 2017. | Excluded by title |
| 595 | M. O. Gabriel, T. Grunheid and A. Zentner. Glycosylation pattern and cell attachment-inhibiting property of human salivary mucins. 2005. | Excluded by title |
| 596 | M. O. Klein, P. W. Kämmerer, T. Scholz, M. Moergel, C. M. Kirchmaier and B. Al-Nawas. Modulation of platelet activation and initial cytokine release by alloplastic bone substitute materials. 2010. | Excluded by title |
| 597 | M. P. Mills, P. S. Rosen, L. Chambrone, H. Greenwell, R. T. Kao, P. R. Klokkevold, B. S. McAllister, M. A. Reynolds, G. E. Romanos and H. L. Wang. American Academy of Periodontology best evidence consensus statement on the efficacy of laser therapy used alone or as an adjunct to non-surgical and surgical treatment of periodontitis and peri-implant diseases. 2018. | Excluded by title |
| 598 | M. Peñarrocha-Diago, A. Aloy-Prósper, D. Peñarrocha-Oltra, J. L. C. Guirado and M. Peñarrocha-Diago. Localized lateral alveolar ridge augmentation with block bone grafts: Simultaneous versus delayed implant placement: A clinical and radiographic retrospective study. 2013. | Excluded by title |
| 599 | M. Penarrocha-Diago, A. Boronat-Lopez and B. Garcia-Mira. Inflammatory implant periapical lesion: etiology, diagnosis, and treatment--presentation of 7 cases. 2009. | Excluded by title |
| 600 | M. Peñarrocha-Diago, R. Alonso-González, A. Aloy-Prósper, D. Peñarrocha-Oltra, F. Camacho and M. Peñarrocha-Diago. Use of buccal fat pad to repair post-extraction peri-implant bone defects in the posterior maxilla. A preliminary prospective study. 2015. | Excluded by title |
| 601 | M. Perán, M. A. García, E. Lopez-Ruiz, G. Jiménez and J. A. Marchal. How can nanotechnology help to repair the body? Advances in cardiac, skin, bone, cartilage and nerve tissue regeneration. 2013. | Excluded by title |
| 602 | M. Pourhajibagher and A. Bahador. In silico identification of a therapeutic target for photo-activated disinfection with indocyanine green: Modeling and virtual screening analysis of Arg-gingipain from Porphyromonas gingivalis. 2017. | Excluded by title |
| 603 | M. R. Karimi, A. Hasani and S. Khosroshahian. Efficacy of Antimicrobial Photodynamic Therapy as an Adjunctive to Mechanical Debridement in the Treatment of Peri-implant Diseases: A Randomized Controlled Clinical Trial. 2016. | Excluded by title |
| 604 | M. Rehman, A. Madni and T. J. Webster. The era of biofunctional biomaterials in orthopedics: what does the future hold?. 2018. | Excluded by title |
| 605 | M. Riool, L. De Boer, V. Jaspers, C. M. Van Der Loos, W. J. B. Van Wamel, G. Wu, P. H. S. Kwakman and S. A. J. Zaat. Staphylococcus epidermidis originating from titanium implants infects surrounding tissue and immune cells. 2014. | Excluded by title |
| 606 | M. Rismanchian, S. Nosouhian, M. Shahabouee, A. Davoudi and F. Nourbakhshian. Effect of conventional and contemporary disinfectant techniques on three peri-implantitis associated microbiotas. 2017. | Excluded by title |
| 607 | M. Roccuzzo, G. Grasso and P. Dalmasso. Keratinized mucosa around implants in partially edentulous posterior mandible: 10-year results of a prospective comparative study. 2016. | Excluded by title |
| 608 | M. Roccuzzo, M. Savoini, P. Dalmasso and G. Ramieri. Long-term outcomes of implants placed after vertical alveolar ridge augmentation in partially edentulous patients: a 10-year prospective clinical study. 2017. | Excluded by title |
| 609 | M. Roncati, A. Lucchese and F. Carinci. Non-surgical treatment of peri-implantitis with the adjunctive use of an 810-nm diode laser. 2013. | Excluded by title |
| 610 | M. S. Marques, K. M. Zepon, F. C. Petronilho, V. Soldi and L. A. Kanis. Characterization of membranes based on cellulose acetate butyrate/poly(caprolactone)triol/doxycycline and their potential for guided bone regeneration application. 2017. | Excluded by title |
| 611 | M. Sanz and M. Simion. Surgical techniques on periodontal plastic surgery and soft tissue regeneration: consensus report of Group 3 of the 10th European Workshop on Periodontology. 2014. | Excluded by title |
| 612 | M. Sartori, G. Giavaresi, A. Parrilli, A. Ferrari, N. N. Aldini, M. Morra, C. Cassinelli, D. Bollati and M. Fini. Collagen type I coating stimulates bone regeneration and osteointegration of titanium implants in the osteopenic rat. 2015. | Excluded by title |
| 613 | M. Schlund, G. Raoul, J. Ferri and R. Nicot. Mandibular Osteomyelitis Following Implant Placement. 2017. | Excluded by title |
| 614 | M. Shahabooei, S. M. Razavi, M. Minaiyan, R. Birang, P. Behfarnia, J. Yaghini, N. Naghsh, P. Ghalayani and S. Hajisadeghi. A histomorphometric study of the effect of doxycycline and erythromycin on bone formation in dental alveolar socket of rat. 2015. | Excluded by title |
| 615 | M. Shimabukuro, Y. Tsutsumi, R. Yamada, M. Ashida, P. Chen, H. Doi, K. Nozaki, A. Nagai and T. Hanawa. Investigation of Realizing Both Antibacterial Property and Osteogenic Cell Compatibility on Titanium Surface by Simple Electrochemical Treatment. 2019. | Excluded by title |
| 616 | M. Shimogishi, Y. Tsutsumi, S. Kuroda, M. Munakata, T. Hanawa and S. Kasugai. Effects of acidic sodium fluoride-treated, commercially pure titanium on periodontal pathogens and rat bone marrow cells. 2014. | Excluded by title |
| 617 | M. Shlezinger, L. Khalifa, Y. Houri-Haddad, S. Coppenhagen-Glazer, G. Resch, Y. A. Que, S. Beyth, E. Dorfman, R. Hazan and N. Beyth. Phage Therapy: A New Horizon in the Antibacterial Treatment of Oral Pathogens. 2017. | Excluded by title |
| 618 | M. Taba, Jr., Q. Jin, J. V. Sugai and W. V. Giannobile. Current concepts in periodontal bioengineering. 2005. | Excluded by title |
| 619 | M. Taberner-Vallverdú, M. Á. Sánchez-Garcés and C. Gay-Escoda. Efficacy of different methods used for dry socket prevention and risk factor analysis: A systematic review. 2017. | Excluded by title |
| 620 | M. Tatullo, M. Marrelli and F. Paduano. The regenerative medicine in oral and maxillofacial surgery: The most important innovations in the clinical application of mesenchymal stem cells. 2015. | Excluded by title |
| 621 | M. Troeltzsch, C. Pache, M. Troeltzsch, G. Kaeppler, M. Ehrenfeld, S. Otto and F. Probst. Etiology and clinical characteristics of symptomatic unilateral maxillary sinusitis: A review of 174 cases. 2015. | Excluded by title |
| 622 | M. Troeltzsch, D. Cagna, P. Stahler, F. Probst, G. Kaeppler, M. Troeltzsch, M. Ehrenfeld and S. Otto. Clinical features of peri-implant medication-related osteonecrosis of the jaw: Is there an association to peri-implantitis?. 2016. | Excluded by title |
| 623 | M. Yang, P. Jiang, Y. Ge, F. Lan, X. Zhou, J. He and Y. Wu. Dopamine self-polymerized along with hydroxyapatite onto the preactivated titanium percutaneous implants surface to promote human gingival fibroblast behavior and antimicrobial activity for biological sealing. 2018. | Excluded by title |
| 624 | M. Yu, X. M. Hui, F. Wang, N. Zhang, H. P. Zhang and Y. L. Song. [Effect of sustained release of recombinant rat insulin-like growth factor-1 from poly (lactide-CO-glycolide ) microspheres on bone formation in the peri-implant areas in Goto-Kakizaki rats with type 2 diabetes]. 2010. | Excluded by title |
| 625 | M. Zurlohe, A. Ortiz-Vigón and A. Bascones Martínez. Tratamiento no quirúrgico en el tratamiento de periimplantitis. Revisión narrativa. 2014. | Excluded by title |
| 626 | M√ºller, J. and Junker, R.(2022); Customized Titanium Mesh for Guided Bone Regeneration in the Posterior Mandible in a Patient Previously Treated with Bisphosphonates. Case Reports in Dentistry,E3 , | Excluded by title |
| 627 | Manifar, S. and Koopaie, M. and Jahromi, Z. M. and Kolahdooz, S.(2023); Effect of synbiotic mouthwash on oral mucositis induced by radiotherapy in oral cancer patients: a double-blind randomized clinical trial. Supportive Care in Cancer,E3 ,111 | Excluded by title |
| 628 | Martins, A. F. L. and Pereira, C. H. and Morais, M. O. and de Sousa-Neto, S. S. and Valadares, M. C. and Freitas, N. M. A. and Leles, C. R. and de Mendon√ßa, E. F.(2023); Effects of a mucoadhesive phytomedicine (<i>Curcuma longa</i> L. and <i>Bidens pilosa</i> L<i>.</i>) on radiotherapy-induced oral mucositis and quality of life of patients undergoing head and neck cancer treatment: randomized clinical trial. Supportive Care in Cancer,E3 ,914 | Excluded by title |
| 629 | Materni, A. and Pasquale, C. and Longo, E. and Frosecchi, M. and Benedicenti, S. and Bozzo, M. and Amaroli, A.(2023); Prevention of Dry Socket with Ozone Oil-Based Gel after Inferior Third Molar Extraction: A Double-Blind Split-Mouth Randomized Placebo-Controlled Clinical Trial. Gels,E3 ,412 | Excluded by title |
| 630 | Matthes, R. and Jablonowski, L. and Pitchika, V. and Holtfreter, B. and Eberhard, C. and Gerling, T. and Wagner, J. and Floerke, C. and Eisenbeiss, A. K. and Cosgarea, R. and Jepsen, K. and Bunke, J. and Ramanauskaite, A. and Begic, A. and Obreja, K. and Mksoud, M. and Kocher, T.(2024); Training in the use of the water jet and cold atmospheric plasma jet for the decontamination of dental implants. Clinical Oral Investigations,E3 ,611 | Excluded by title |
| 631 | Mazraeh, E. J. and Sadighi, S. and Manifar, S. and Bakhshandeh, H. and Rajabi, M.(2023); Assessment of thyme honey oral gel for the prevention of adriamycin and cyclophosphamide chemotherapy-induced oral mucositis in patients with breast cancer. Supportive Care in Cancer,E3 ,810 | Excluded by title |
| 632 | Mehr, A. K. and Pourabbas, R. and Khajeh, M. S.(2018); Effect of photodynamic therapy with toluidine blue photosensitizer on nonsurgical management of peri-implant mucosal inflammation. Annals of Tropical Medicine and Public Health,E3 ,2SP45 | Excluded by title |
| 633 | Mehrabi, A. and Negahdari, R. and Parnia, F. and Garjani, A.(2021); Effect of tetracycline on IL-1Œ≤ and IL-6 levels of the peri-implant sulcular fluid. Journal of Advanced Periodontology and Implant Dentistry,E3 ,256-60 | Excluded by title |
| 634 | Mohammed, A. I. and Fedoruk, L. and Fisher, N. and Liu, A. X. and Khanna, S. and Naylor, K. and Gong, Z. Y. and Celentano, A. and Alrashdan, M. S. and Cirillo, N.(2024); Systemic Anti-Inflammatory Agents in the Prevention of Chemoradiation-Induced Mucositis: A Review of Randomised Controlled Trials. Biomolecules,E3 ,515 | Excluded by title |
| 635 | Morsy, B. M. and El Domiaty, S. and Meheissen, M. A. M. and Heikal, L. A. and Meheissen, M. A. and Aly, N. M.(2023); Omega-3 nanoemulgel in prevention of radiation-induced oral mucositis and its associated effect on microbiome: a randomized clinical trial. Bmc Oral Health,E3 ,112 | Excluded by title |
| 636 | N. A. Valente and S. Andreana. Treatment of Peri-implantitis Using a Combined Decontaminative and Regenerative Protocol: Case Report. 2018. | Excluded by title |
| 637 | N. A. Valente, T. Mang, M. Hatton, L. Mikulski and S. Andreana. Effects of Two Diode Lasers With and Without Photosensitization on Contaminated Implant Surfaces: An Ex Vivo Study. 2017. | Excluded by title |
| 638 | N. Caplanis, T. J. Sigurdsson, M. D. Rohrer and U. M. E. Wikesjo. Effect of allogeneic, freeze-dried, demineralized bone matrix on guided bone regeneration in supra-alveolar peri-implant defects in dogs. 1997. | Excluded by title |
| 639 | N. Casap, C. Zeltser, A. Wexler, E. Tarazi and R. Zeltser. Immediate Placement of Dental Implants Into Debrided Infected Dentoalveolar Sockets. 2007. | Excluded by title |
| 640 | N. De Angelis, P. Felice, G. Pellegrino, A. Camurati, P. Gambino and M. Esposito. Guided bone regeneration with and without a bone substitute at single post-extractive implants: 1-year post-loading results from a pragmatic multicentre randomised controlled trial. 2011. | Excluded by title |
| 641 | N. Eliaz and N. Metoki. Calcium phosphate bioceramics: A review of their history, structure, properties, coating technologies and biomedical applications. 2017. | Excluded by title |
| 642 | N. Jiang, P. Du, W. Qu, L. Li, Z. Liu and S. Zhu. The synergistic effect of TiO2 nanoporous modification and platelet-rich plasma treatment on titanium-implant stability in ovariectomized rats. 2016. | Excluded by title |
| 643 | N. L. Euctr. Effect of Delmopinol on treatment of inflammation around dental implants. 2016. | Excluded by title |
| 644 | N. Mahato, X. Wu and L. Wang. Management of peri-implantitis: a systematic review, 2010-2015. 2016. | Excluded by title |
| 645 | N. Mardas, X. Dereka, N. Donos and M. Dard. Experimental model for bone regeneration in oral and cranio-maxillo-facial surgery. 2014. | Excluded by title |
| 646 | N. Mattheos, S. Collier and A. D. Walmsley. Specialists' management decisions and attitudes towards mucositis and peri-implantitis. 2012. | Excluded by title |
| 647 | N. P. Lang, A. Mombelli, M. S. Tonetti, U. Bragger and C. H. Hammerle. Clinical trials on therapies for peri-implant infections. 1997. | Excluded by title |
| 648 | N. Savickiene, A. Jekabsone, L. Raudone, A. S. Abdelgeliel, A. Cochis, L. Rimondini, E. Makarova, S. Grinberga, O. Pugovics, M. Dambrova, I. M. Pacauskiene, N. Baseviciene and P. Viskelis. Efficacy of Proanthocyanidins from Pelargonium sidoides Root Extract in Reducing P. gingivalis Viability While Preserving Oral Commensal S. salivarius. 2018. | Excluded by title |
| 649 | N. Yan, X. Zhang, Q. Cai, X. Yang, X. Zhou, B. Wang and X. Deng. The effects of lactidyl/glycolidyl ratio and molecular weight of poly(D,L-lactide-co-glycolide) on the tetracycline entrapment and release kinetics of drug-loaded nanofibers. 2012. | Excluded by title |
| 650 | Naeini, E. N. and Atashkadeh, M. and Jacquet, W. and D‚ÄôHaese, J. and De Bruyn, H.(2023); Incidence of Peri-Implantitis, Technical and Biological Complications of Single Implants Placed with Flap or Flapless Surgery‚ÄîA 10‚Äì12-Year Case-Series. Journal of Clinical Medicine,E3 ,11 | Excluded by title |
| 651 | Naeini, E. N. and De Bruyn, H. and Bronkhorst, E. M. and D‚Äôhaese, J.(2022); Case Series on the Long-Term Effect of Three Different Types of Maxillary Implant-Supported Overdentures on Clinical Outcomes and Complications. Journal of Clinical Medicine,E3 ,8 | Excluded by title |
| 652 | Naiem, S. N. and Hosny, M. and ElNahass, H.(2023); Esthetics and bone Changes of Immediate Implants with or without Vascularized Interpositional Periosteal Connective Tissue Grafting: a 2-year Randomized Controlled Trial. Clinical oral implants research,E3 ,5498‚Äê511 | Excluded by title |
| 653 | Naumann, M. and Scholz, P. and Krois, J. and Schwendicke, F. and Sterzenbach, G. and Happe, A.(2023); Monolithic hybrid abutment crowns (screw-retained) versus monolithic hybrid abutments with adhesively cemented monolithic crowns. Clinical Oral Implants Research,E3 ,3209-220 | Excluded by title |
| 654 | Nct(2023); Antibacterial Photodynamic Therapy in the Management of Peri-implantitis. https://clinicaltrials.gov/ct2/show/NCT06017817,E3 , | Excluded by title |
| 655 | Nct(2023); Application of Information-Motivation-Behavioral Model-based Continuity of Care on the Peri-implantitis Recovery in Diabetic Implant Overdenture Patients. https://clinicaltrials.gov/ct2/show/NCT06103799,E3 , | Excluded by title |
| 656 | Nct(2023); Bacterial Microleakage and Bone Loss in Internal Connection Dental Implants Based on the Type of Abutment Used in Zirconia Partial Fixed Prosthesis: in Vivo Study. https://clinicaltrials.gov/ct2/show/NCT06156111,E3 , | Excluded by title |
| 657 | Nct(2023); Biological Responses Affecting Early-stage Dental Implant Placement in Patients With History of Periodontitis. https://clinicaltrials.gov/show/NCT05834946,E3 , | Excluded by title |
| 658 | Nct(2023); Clinical Efficiency of Xanthan Hydrogels Containing Local Anesthetics Encapsulated in Nanostructured Lipid Carries. https://clinicaltrials.gov/show/NCT05912335,E3 , | Excluded by title |
| 659 | Nct(2023); Comparing Two Different Tunneling Technique for Gingival Recession Treatment Using Two Different Matertial. https://clinicaltrials.gov/ct2/show/NCT06065774,E3 , | Excluded by title |
| 660 | Nct(2023); Curcumin VS Photo-bio-modulation Therapy of Oral Mucositis in Pediatric Patients Undergoing Anti-Cancer Non-invasive Treatment. https://clinicaltrials.gov/ct2/show/NCT06044142,E3 , | Excluded by title |
| 661 | Nct(2023); Effectiveness of Meshed Free Gingival Graft for Widening of Keratinized Tissues. https://clinicaltrials.gov/ct2/show/NCT06037694,E3 , | Excluded by title |
| 662 | Nct(2023); Efficacy of A Novel RinSe Device to Reduce Oral Bacteria in Intubated IntEnsive CaRe Patients: a Pilot Study. https://clinicaltrials.gov/ct2/show/NCT06193512,E3 , | Excluded by title |
| 663 | Nct(2023); Efficacy of Air-polishing on Pain Perception and Compliance Rate. https://clinicaltrials.gov/ct2/show/NCT06109701,E3 , | Excluded by title |
| 664 | Nct(2023); Elamrousy Modified Approach for Socket Shield Technique. https://clinicaltrials.gov/ct2/show/NCT06043037,E3 , | Excluded by title |
| 665 | Nct(2023); Er,Cr: YSGG Laser Application in Peri-implantitis. https://clinicaltrials.gov/ct2/show/NCT06031467,E3 , | Excluded by title |
| 666 | Nct(2023); Evaluation of Prophylactic Photobiomodulation Therapy in Patients With Osteosarcoma. https://clinicaltrials.gov/ct2/show/NCT06217224,E3 , | Excluded by title |
| 667 | Nct(2023); Glucosamine Sulphate Versus Ginger in Non-Surgical Periodontal Therapy. https://clinicaltrials.gov/ct2/show/NCT06200415,E3 , | Excluded by title |
| 668 | Nct(2023); Immediate Versus Delayed Loading of Maxillary Overdenture Implants. https://clinicaltrials.gov/ct2/show/NCT06038487,E3 , | Excluded by title |
| 669 | Nct(2023); Impact of Non-surgical Periodontal Therapy in the Improvement of Early Endothelial Dysfunction in Subjects With Peri-implantitis and Peri-implant Mucositis. https://clinicaltrials.gov/ct2/show/NCT05906810,E3 , | Excluded by title |
| 670 | Nct(2023); Investigation of the Effect of Oral Care With Coconut Oil on the Degree of Oral Mucositis in Pediatric Oncology Patients. https://clinicaltrials.gov/show/NCT05849571,E3 , | Excluded by title |
| 671 | Nct(2023); Low Power Laser Therapy As Prevention Of Oral Mucositis And Oropharyngeal Pain In Patients Undergoing Allogenetic HSCT. https://clinicaltrials.gov/ct2/show/NCT06071637,E3 , | Excluded by title |
| 672 | Nct(2023); Marginal Bone Changes in Fixed All-on-Four Mandibular Prosthesis Using OT Bridge Attachment System. https://clinicaltrials.gov/show/NCT05924386,E3 , | Excluded by title |
| 673 | Nct(2023); Oro-nasal Decontamination to Prevent Ventilator-associated Pneumonia. https://clinicaltrials.gov/show/NCT05895773,E3 , | Excluded by title |
| 674 | Nct(2023); Prevention of Dental Implant Diseases. https://clinicaltrials.gov/show/NCT05871229,E3 , | Excluded by title |
| 675 | Nct(2023); Ridge Splitting With Implant Placement Using Autogenous Tooth Graft. https://clinicaltrials.gov/ct2/show/NCT06131541,E3 , | Excluded by title |
| 676 | Nct(2023); The Efficacy of Nigella Sativa Oil Mouth Rinse in the Management of Recurrent Minor Aphthous Ulcer. https://clinicaltrials.gov/ct2/show/NCT06013202,E3 , | Excluded by title |
| 677 | Nct(2023); Treatment of Peri-implant Mucositis and Supportive Peri-implant Therapy. https://clinicaltrials.gov/ct2/show/NCT06137846,E3 , | Excluded by title |
| 678 | Nct(2023); Treatment of Peri-implant Mucositis by Sodium Hypochlorite Gel and Cross-linked Hyaluronic Acid Gel. https://clinicaltrials.gov/ct2/show/NCT05926297,E3 , | Excluded by title |
| 679 | Nct(2023); Using of Collagen Matrix for Maxillary Tuberosity Donor Area Preservation. https://clinicaltrials.gov/ct2/show/NCT06039839,E3 , | Excluded by title |
| 680 | Nct(2023); Vertical Versus Inclined Implant to Retain Locator Maxillary Overdenture. https://clinicaltrials.gov/show/NCT05843331,E3 , | Excluded by title |
| 681 | Nct(2024); Adjunctive Use of Dermal Matrix to Compensate Dimensional Changes in the Reconstructive Therapy of Peri-implantitis. https://clinicaltrials.gov/ct2/show/NCT06398288,E3 , | Excluded by title |
| 682 | Nct(2024); Anti-reflux Mucosal Valvuloplasty Versus PPIs for GERD Treatment. https://clinicaltrials.gov/ct2/show/NCT06348420,E3 , | Excluded by title |
| 683 | Nct(2024); Chitosan Brushes vs Air-Abrasive Devices on Peri-implant Mucositis Treatment: a Randomized Clinical. https://clinicaltrials.gov/ct2/show/NCT06287957,E3 , | Excluded by title |
| 684 | Nct(2024); Comparison Between the Effect of Using Conventional and Digital Oral Positional Radiation Stent. https://clinicaltrials.gov/ct2/show/NCT06353724,E3 , | Excluded by title |
| 685 | Nct(2024); Comparison of Dental Implants With Different Surface Properties. https://clinicaltrials.gov/ct2/show/NCT06508723,E3 , | Excluded by title |
| 686 | Nct(2024); Effect of Aloe Vera Gel and Manuka Honey on Radiation Induced Oral Mucositis. https://clinicaltrials.gov/ct2/show/NCT06381635,E3 , | Excluded by title |
| 687 | Nct(2024); Effect of Free Gingival Grafting on Peri-implant Health. https://clinicaltrials.gov/ct2/show/NCT06392256,E3 , | Excluded by title |
| 688 | Nct(2024); Effect of Hawthorn Vinegar, Black Mulberry Syrup and Green Tea on Oral Mucositis. https://clinicaltrials.gov/ct2/show/NCT06481254,E3 , | Excluded by title |
| 689 | Nct(2024); Efficacy of Non-surgical and Surgical Surface Decontamination Methods on Peri-implantitis-affected Implants. https://clinicaltrials.gov/ct2/show/NCT06430268,E3 , | Excluded by title |
| 690 | Nct(2024); Efficacy of Thyme Honey in The Management of Oral Aphthous Ulcers. https://clinicaltrials.gov/ct2/show/NCT06421038,E3 , | Excluded by title |
| 691 | Nct(2024); Elamrousy Novel Approach of Socket Shield Technique. https://clinicaltrials.gov/ct2/show/NCT06263842,E3 , | Excluded by title |
| 692 | Nct(2024); Erythritol Air Polishing in Mucositis Treatment. https://clinicaltrials.gov/ct2/show/NCT06455306,E3 , | Excluded by title |
| 693 | Nct(2024); Immediate Dental Implants in The Upper Anterior Region. https://clinicaltrials.gov/ct2/show/NCT06382337,E3 , | Excluded by title |
| 694 | Nct(2024); Impact of Pulsed Electromagnetic Field (PEMF) on Human Peri-implant Tissues. https://clinicaltrials.gov/ct2/show/NCT06516523,E3 , | Excluded by title |
| 695 | Nct(2024); Lactobacillus Reuteri Alleviates Oral Mucositis in Patients Undergoing Radiotherapy for Malignant Head and Neck Tumors. https://clinicaltrials.gov/ct2/show/NCT06285591,E3 , | Excluded by title |
| 696 | Nct(2024); Leveraging Telehealth to Improve Oral Health Among Cancer Survivors. https://clinicaltrials.gov/ct2/show/NCT06315855,E3 , | Excluded by title |
| 697 | Nct(2024); Maxillary Labial Frenectomy: diode Lasers Versus Surgical Scalpel. https://clinicaltrials.gov/ct2/show/NCT06548516,E3 , | Excluded by title |
| 698 | Nct(2024); Non-Surgical Treatment of Peri-Implantitis With Ultrasonic Carbon Tip. https://clinicaltrials.gov/ct2/show/NCT06514677,E3 , | Excluded by title |
| 699 | Nct(2024); PROSpECT-PRIOR-2-CHEMO: PRIOR Dental Intervention Before Chemo to Reduce Chemotherapy Complications. https://clinicaltrials.gov/ct2/show/NCT06450821,E3 , | Excluded by title |
| 700 | Nct(2024); The Efficacy of Thyme Honey Mouth Rinse on Polypharmacy-induced Xerostomia. https://clinicaltrials.gov/ct2/show/NCT06201923,E3 , | Excluded by title |
| 701 | Nct(2024); Treatment of Peri-implant Bone Dehiscence Using Autogenous Tooth Plate. https://clinicaltrials.gov/ct2/show/NCT06313216,E3 , | Excluded by title |
| 702 | Nct(2024); Ultrasonographic Evaluation of the Connective Tissue Grafts Obtained With Two Different Methods in Root Coverage. https://clinicaltrials.gov/ct2/show/NCT06373783,E3 , | Excluded by title |
| 703 | Nct. 0.2% Chx Gel vs Implant Bacterial Contamination. 2018. | Excluded by title |
| 704 | Nct. A New Approach for Controlling Hemostasis During Canal Treatment. 2017. | Excluded by title |
| 705 | Nct. Anti-infective Effect of Non-surgical Treatment With and Without Photodynamic Therapy on Initial Peri-implantitis. 2016. | Excluded by title |
| 706 | Nct. Assessment of Immediate Implant Stability When Adding Mixture of Hyaluronic Acid and Melatonin. 2018. | Excluded by title |
| 707 | Nct. Clinical Evaluation of Vertical Inter-implant Papilla Height in Platform Matched Implants in Comparison to Morse Connection Abutments. 2018. | Excluded by title |
| 708 | Nct. Dental Implant Placement in Adjunction With Autologous Alveolar Bone-Marrow Derived Mesenchymal Stem Cells (aBM-MSCs). 2017. | Excluded by title |
| 709 | Nct. Diode Laser for Treatment of Peri-implantitis. 2017. | Excluded by title |
| 710 | Nct. Efficacy of 0.12% Chlorhexidine Gluconate for Peri-implant Mucositis and Gingivitis. 2015. | Excluded by title |
| 711 | Nct. Efficacy of 0.12% Chlorhexidine Gluconate for Peri-implant Mucositis. 2015. | Excluded by title |
| 712 | Nct. Efficacy of Diode Laser in Peri-implantitis. 2014. | Excluded by title |
| 713 | Nct. Metronidazole as an Adjunct of Non- Surgical Treatment of Peri-implantitis. 2018. | Excluded by title |
| 714 | Nct. Ozone Therapy as an Adjunct to the Surgical Treatment of Peri-implantitis. 2017. | Excluded by title |
| 715 | Nct. Patient Satisfaction, Peri-implant Parameters and Crestal Bone Evaluation of PEEK (Polyetheretherketone) Abutments Versus Zirconium Abutments Restored With PEEK Based Superstructure. 2018. | Excluded by title |
| 716 | Nct. Regenerative Surgical Treatment of Peri-implantitis. 2015. | Excluded by title |
| 717 | Nct. Ridge Regeneration in Three Wall Sockets. 2018. | Excluded by title |
| 718 | Nct. Surgical Treatment of Peri-implantitis. 2017. | Excluded by title |
| 719 | Nct. The Effect of Platform-Matching Versus Platform-Switching Dental Implants on Peri-implant Hard and Soft Tissue Healing. 2015. | Excluded by title |
| 720 | Nct. The Influence of Mucosal Tissue Thickness on Soft and Hard Tissue Changes Around Implants. 2015. | Excluded by title |
| 721 | Nct. Triclosan Toothpaste in the Maintenance Phase of Peri-implantitis Treatment. 2017. | Excluded by title |
| 722 | Nct. Use of Topical Subgingival Application of Simvastatin Gel in the Treatment of Peri-Implant Mucositis. 2018. | Excluded by title |
| 723 | Nickles, K. and Krebs, M. and Schacher, B. and Petsos, H. and Eickholz, P.(2022); Long-Term Results after Placing Dental Implants in Patients with Papillon-Lef√®vre Syndrome: Results 2.5‚Äì20 Years after Implant Insertion. Journal of Clinical Medicine,E3 ,9 | Excluded by title |
| 724 | Nicola, D. and Isabella, D. and Carolina, C. and Baldini, N. and Raffaele, M.(2024); Treatment of peri-implant mucositis: Adjunctive effect of glycine powder air polishing to professional mechanical biofilm removal. 12 months randomized clinical study. Clinical Implant Dentistry and Related Research,E3 ,2415-426 | Excluded by title |
| 725 | No disponible. . . | Excluded by title |
| 726 | Nobre, M. A. and Guedes, C. M. and Almeida, R. and Silva, A. and Sereno, N.(2020); Hybrid polyetheretherketone (Peek)‚Äìacrylic resin prostheses and the all-on-4 concept: A full-arch implant-supported fixed solution with 3 years of follow-up. Journal of Clinical Medicine,E3 ,743101 | Excluded by title |
| 727 | Nobre, M. A. and Salvado, F. and Nogueira, P. and Rocha, E. and Ilg, P. and Mal√≥, P.(2019); A prognostic model for the outcome of nobel biocare dental implants with peri-implant disease after one year. Journal of Clinical Medicine,E3 ,9 | Excluded by title |
| 728 | Ntr. Non-surgical treatment of peri-implantitis. 2012. | Excluded by title |
| 729 | O. Carcuac, I. Abrahamsson, G. Charalampakis and T. Berglundh. The effect of the local use of chlorhexidine in surgical treatment of experimental peri-implantitis in dogs. 2015. | Excluded by title |
| 730 | O. Cipriani, V. Vellone, P. Arangio and F. Della Rocca. Sinus Disventilation and Atrophy of the Upper Maxilla: A Combined Surgical Approach Is Possible?. 2017. | Excluded by title |
| 731 | O. I. Larsen, M. Enersen, A. K. Kristoffersen, A. Wennerberg, D. F. Bunaes, S. A. Lie and K. N. Leknes. Antimicrobial Effects of Three Different Treatment Modalities on Dental Implant Surfaces. 2017. | Excluded by title |
| 732 | O. M. Goudouri, E. Kontonasaki, U. Lohbauer and A. R. Boccaccini. Antibacterial properties of metal and metalloid ions in chronic periodontitis and peri-implantitis therapy. 2014. | Excluded by title |
| 733 | O. Salomo-Coll, J. E. Mate-Sanchez de Val, M. P. Ramirez-Fernandez, F. Hernandez-Alfaro, J. Gargallo-Albiol and J. L. Calvo-Guirado. Topical applications of vitamin D on implant surface for bone-to-implant contact enhance: a pilot study in dogs part II. 2016. | Excluded by title |
| 734 | O. Teronen, Y. T. Konttinen, C. Lindqvist, T. Salo, T. Ingman, A. Lauhio, Y. Ding, S. Santavirta and T. Sorsa. Human neutrophil collagenase MMP-8 in peri-implant sulcus fluid and its inhibition by clodronate. 1997. | Excluded by title |
| 735 | O. Z. Andersen, V. Offermanns, M. Sillassen, K. P. Almtoft, I. H. Andersen, S. Sorensen, C. S. Jeppesen, D. C. Kraft, J. Bottiger, M. Rasse, F. Kloss and M. Foss. Accelerated bone ingrowth by local delivery of strontium from surface functionalized titanium implants. 2013. | Excluded by title |
| 736 | Oliveira, F. M. and Borges, M. M. and Malta, C. E. and Moura, J. F. and Forte, C. P. and Barbosa, J. V. and Silva, P. G. and Dantas, T. S.(2024); Comparison of a daily and alternate-day photobiomodulation protocol in the prevention of oral mucositis in patients undergoing radiochemotherapy for oral cancer: a triple-blind, controlled clinical trial. Medicina oral, patologia oral y cirugia bucal,E3 ,3e430‚Äêe440 | Excluded by title |
| 737 | Ono, K. and Ueno, T. and Kido, M. A. and Hitomi, S. and Naniwa, M. and Nakatomi, C. and Yoshimoto, R. U. and Sawada, T. and Kato, T.(2024); Recent advances in the treatment of oral ulcerative mucositis from clinical and basic perspectives. Journal of Oral Biosciences,E3 ,3504-510 | Excluded by title |
| 738 | P. A. Norowski, Jr. and J. D. Bumgardner. Biomaterial and antibiotic strategies for peri-implantitis: a review. 2009. | Excluded by title |
| 739 | P. Avci, M. Karimi, M. Sadasivam, W. C. Antunes-Melo, E. Carrasco and M. R. Hamblin. In-vivo monitoring of infectious diseases in living animals using bioluminescence imaging. 2018. | Excluded by title |
| 740 | P. C. Aravena, C. P. Oyarzun, M. F. Arias, H. Monardes, A. Jerez and B. Benso. Single-Dose Bioavailability for Prophylactic Coverage in Patients Undergoing Dental Implant Surgery. 2018. | Excluded by title |
| 741 | P. C. Smith, C. Martínez, M. Cáceres and J. Martínez. Research on growth factors in periodontology. 2015. | Excluded by title |
| 742 | P. Chondros, D. Nikolidakis, N. Christodoulides, R. Rossler, N. Gutknecht and A. Sculean. Photodynamic therapy as adjunct to non-surgical periodontal treatment in patients on periodontal maintenance: a randomized controlled clinical trial. 2009. | Excluded by title |
| 743 | P. E. Bottura, J. Milanezi, L. A. Femandes, H. C. Caldas, M. Abbud, V. G. Garcia and M. Baptista. Nonsurgical Periodontal Therapy Combined with Laser and Photodynamic Therapies for Periodontal Disease in Immunosuppressed Rats. 2011. | Excluded by title |
| 744 | P. Hassanzadeh, F. Atyabi and R. Dinarvand. Tissue engineering: Still facing a long way ahead. 2018. | Excluded by title |
| 745 | P. Heasman, Z. Esmail and C. Barclay. Peri-implant diseases. 2010. | Excluded by title |
| 746 | P. J. Schoen, G. M. Raghoebar, J. Bouma, H. Reintsema, A. Vissink, W. Sterk and J. L. Roodenburg. Rehabilitation of oral function in head and neck cancer patients after radiotherapy with implant-retained dentures: effects of hyperbaric oxygen therapy. 2007. | Excluded by title |
| 747 | P. K. Sreenivasan, Y. Vered, A. Zini, J. Mann, H. Kolog, D. Steinberg, J. J. Zambon, V. I. Haraszthy, M. P. da Silva and W. De Vizio. A 6-month study of the effects of 0.3% triclosan/copolymer dentifrice on dental implants. 2011. | Excluded by title |
| 748 | P. Korn, M. C. Schulz, V. Hintze, U. Range, R. Mai, U. Eckelt, M. Schnabelrauch, S. Möller, J. Becher, D. Scharnweber and B. Stadlinger. Chondroitin sulfate and sulfated hyaluronan-containing collagen coatings of titanium implants influence peri-implant bone formation in a minipig model. 2014. | Excluded by title |
| 749 | P. M. Lambert, H. F. Morris and S. Ochi. The influence of smoking on 3-year clinical success of osseointegrated dental implants. 2000. | Excluded by title |
| 750 | P. Mandracci, F. Mussano, P. Rivolo and S. Carossa. Surface treatments and functional coatings for biocompatibility improvement and bacterial adhesion reduction in dental implantology. 2016. | Excluded by title |
| 751 | P. Meisel and T. Kocher. Photodynamic therapy for periodontal diseases: state of the art. 2005. | Excluded by title |
| 752 | P. O. Tessare Jr and M. B. Fonseca. Terapia fotodinâmica aplicada na periimplantite. 2008. | Excluded by title |
| 753 | P. Pripatnanont, T. Nuntanaranont, S. Vongvatcharanon and K. Phurisat. The primacy of platelet-rich fibrin on bone regeneration of various grafts in rabbit's calvarial defects. 2013. | Excluded by title |
| 754 | P. Ramberg, J. Lindhe, D. Botticelli and A. Botticelli. The effect of a triclosan dentifrice on mucositis in subjects with dental implants: a six-month clinical study. 2009. | Excluded by title |
| 755 | P. Robles Raya, A. P. Javierre Miranda, N. Moreno Millán, A. Mas Casals, E. d. Frutos Echániz and M. L. Morató Agustí. Manejo de las infecciones odontogénicas en las consultas de atención primaria: ¿antibiótico?. 2017. | Excluded by title |
| 756 | P. Robles Raya, A. P. Javierre Miranda, N. Moreno Millan, A. Mas Casals, E. de Frutos Echaniz and M. L. Morato Agusti. [Management of odontogenic infections in Primary Care: Antibiotic?]. 2017. | Excluded by title |
| 757 | P. S. Kumar. Systemic Risk Factors for the Development of Periimplant Diseases. 2019. | Excluded by title |
| 758 | P. S. Rosen, M. Qari, S. J. Froum, S. Dibart and L. L. Chou. A Pilot Study on the Efficacy of a Treatment Algorithm to Detoxify Dental Implant Surfaces Affected by Peri-implantitis. 2018. | Excluded by title |
| 759 | P. S. Talreja, G. V. Gayathri and D. S. Mehta. Treatment of an early failing implant by guided bone regeneration using resorbable collagen membrane and bioactive glass. 2013. | Excluded by title |
| 760 | P. Scolozzi and B. Jaques. Treatment of midfacial defects using prostheses supported by ITI dental implants. 2004. | Excluded by title |
| 761 | P. Slepička, J. Siegel, O. Lyutakov, N. Slepičková Kasálková, Z. Kolská, L. Bačáková and V. Švorčík. Polymer nanostructures for bioapplications induced by laser treatment. 2018. | Excluded by title |
| 762 | Pactr(2024); Immediate Implant Placement Using Tunneling Technique with Connective Tissue Graft versus Coated and Non-Coated DuraGen Matrix with Concentrated Growth Factors. https://trialsearch.who.int/Trial2.aspx?TrialID=PACTR202407826889857,E3 , | Excluded by title |
| 763 | Pantaleo, G. and Acerra, A. and Giordano, F. and D‚ÄôAmbrosio, F. and Langone, M. and Caggiano, M.(2022); Immediate Loading of Fixed Prostheses in Fully Edentulous Jaws: A 7-Year Follow-Up from a Single-Cohort Retrospective Study. Applied Sciences (Switzerland),E3 ,23 | Excluded by title |
| 764 | Park, J. S. and Herr, Y. and Chung, J. H. and Shin, S. I. and Lim, H. C.(2022); Retrospective analysis of keratinized tissue augmentation using a xenogeneic collagen matrix for resolving peri-implant mucositis and peri-implantitis. Journal of Periodontal and Implant Science,E3 ,5 | Excluded by title |
| 765 | Parmar, N.(2023); Antibiotics in the non-surgical treatment of peri-implantitis. Evid Based Dent,E3 ,269-70 | Excluded by title |
| 766 | Parreira, G. J. G. and De Ara√∫jo Nobre, M. A. and Moreira, A. G. C. R. and Lu√≠s, H. P. S.(2024); Limosilactobacillus reuteri as an Adjuvant in the Treatment of Peri-implant Mucositis in Total Rehabilitation: An Exploratory Study. European Journal of Dentistry,E3 ,2610-618 | Excluded by title |
| 767 | Pasquale, C. and De Angelis, N. and Dellacasa, E. and Raiteri, R. and Barberis, F. and Lagazzo, A. and Benedicenti, S. and Amaroli, A.(2023); A Comparative Study on the Effect of Peri-Implant Infection Management Lasers (1064-nm Q-Switch Nd:YAG, 1064-nm Nd:YAG and 980-nm Diode) on Titanium Grade 4 Surface. Applied Sciences-Basel,E3 ,112 | Excluded by title |
| 768 | Passariello, C. and Lucchese, A. and Pera, F. and Gigola, P.(2012); Clinical, microbiological and inflammatory evidence of the efficacy of combination therapy including serratiopeptidase in the treatment of periimplantitis. European Journal of Inflammation,E3 ,3463-472 | Excluded by title |
| 769 | Patil, K. and Sanjay, C. J. and Guledgud, M. V. and Harshitha, N. and Shiny, A. and Suresh, N.(2023); Efficacy of Vitamin D Gel in Curbing and Curing Radiation-Induced Oral Mucositis. Journal of Indian Academy of Oral Medicine and Radiology,E3 ,4488-492 | Excluded by title |
| 770 | Patil, K. and Sanjay, C. J. and Mahima, V. G. and Devi, K. R. R. and Ramesh, A. R. and Shiny, A.(2024); Comparative Efficacy of Zinc Sulfate and Benzydamine Mouthwash in the Management of Radiation-Induced Oral Mucositis. Journal of Indian Academy of Oral Medicine and Radiology,E3 ,145445 | Excluded by title |
| 771 | Pigozzo, M. N. and Cunha, N. and Amorim, K. and Lagana, D. C.(2023); Cumulative success rate and marginal bone loss for immediate and early loading protocols in a single implant-supported crown: a randomized controlled split-mouth clinical trial. Journal of prosthetic dentistry,E3 , | Excluded by title |
| 772 | Polak, D. and Maayan, E. and Chackartchi, T.(2017); The impact of implant design, defect size, and type of superstructure on the accessibility of nonsurgical and surgical approaches for the treatment of peri-implantitis. International Journal of Oral and Maxillofacial Implants,E3 ,2356-362 | Excluded by title |
| 773 | Possebon, A. P. R. and Schuster, A. J. and Chagas-J√∫nior, O. L. and Pinto, L. R. and Faot, F.(2023); Immediate versus conventional loading of mandibular implant-retained overdentures: a 3‚Äêyear follow‚Äêup of a randomized controlled trial. Clinical Oral Investigations,E3 ,105935-5946 | Excluded by title |
| 774 | Pourabbas, R. and Khorramdel, A. and Sadighi, M. and Kashefimehr, A. and Mousavi, S. A.(2023); Effect of photodynamic therapy as an adjunctive to mechanical debridement on the nonsurgical treatment of peri-implant mucositis: a randomized controlled clinical trial. Dental research journal,E3 ,1 | Excluded by title |
| 775 | Puisys, A. and Auzbikaviciute, V. and Simkunaite-Rizgeliene, R. and Razukevicius, D. and Linkevicius, R. and Linkevicius, T.(2019); Bone Remineralization around Dental Implants following Conservative Treatment after Peri-Implantitis. Case Reports in Dentistry,E3 , | Excluded by title |
| 776 | Q. Ma, W. Wang, P. K. Chu, S. Mei, K. Ji, L. Jin and Y. Zhang. Concentration- and time-dependent response of human gingival fibroblasts to fibroblast growth factor 2 immobilized on titanium dental implants. 2012. | Excluded by title |
| 777 | Q. Mao, K. Su, Y. Zhou, M. Hossaini-Zadeh, G. S. Lewis and J. Du. Voxel-based micro-finite element analysis of dental implants in a human cadaveric mandible: Tissue modulus assignment and sensitivity analyses. 2019. | Excluded by title |
| 778 | Q. Shi, K. Song, X. Zhou, Z. Xiong, T. Du, X. Lu and Y. Cao. Effects of non-equilibrium plasma in the treatment of ligature-induced peri-implantitis. 2015. | Excluded by title |
| 779 | Q. Xie, L. N. Jia, H. Y. Xu, X. G. Hu, W. Wang and J. Jia. Fabrication of Core-Shell PEI/pBMP2-PLGA Electrospun Scaffold for Gene Delivery to Periodontal Ligament Stem Cells. 2016. | Excluded by title |
| 780 | R. A. Fahmy, N. Mahmoud, S. Soliman, S. R. Nouh, L. Cunningham and A. El-Ghannam. Acceleration of Alveolar Ridge Augmentation Using a Low Dose of Recombinant Human Bone Morphogenetic Protein-2 Loaded on a Resorbable Bioactive Ceramic. 2015. | Excluded by title |
| 781 | R. Al Habashneh, F. A. Asa'ad and Y. Khader. Photodynamic therapy in periodontal and peri-implant diseases. 2015. | Excluded by title |
| 782 | R. B. R. qhv4f. Evaluation of bone loss around implants with Morse cone connection. 2018. | Excluded by title |
| 783 | R. Bettach, B. Guillaume, S. Taschieri and M. Del Fabbro. Clinical performance of a highly porous beta-TCP as the grafting material for maxillary sinus augmentation. 2014. | Excluded by title |
| 784 | R. Bosco, M. Iafisco, A. Tampieri, J. A. Jansen, S. C. G. Leeuwenburgh and J. J. J. P. Van Den Beucken. Hydroxyapatite nanocrystals functionalized with alendronate as bioactive components for bone implant coatings to decrease osteoclastic activity. 2015. | Excluded by title |
| 785 | R. Burgers, C. Witecy, S. Hahnel and M. Gosau. The effect of various topical peri-implantitis antiseptics on Staphylococcus epidermidis, Candida albicans, and Streptococcus sanguinis. 2012. | Excluded by title |
| 786 | R. C. V. Casarin, F. V. Ribeiro, W. A. Sallum, E. A. Sallum, F. H. Nociti Junior and M. Z. Casati. Utilização de biomateriais e de tecido conjuntivo subepitelial no tratamento de periimplantite: relato de caso clínico. 2007. | Excluded by title |
| 787 | R. Conte, A. Di Salle, F. Riccitiello, O. Petillo, G. Peluso and A. Calarco. Biodegradable polymers in dental tissue engineering and regeneration. 2018. | Excluded by title |
| 788 | R. E. Jung, G. I. Benic, D. Scherrer and C. H. Hammerle. Cone beam computed tomography evaluation of regenerated buccal bone 5 years after simultaneous implant placement and guided bone regeneration procedures--a randomized, controlled clinical trial. 2015. | Excluded by title |
| 789 | R. E. Jung, R. Glauser, P. Scharer, C. H. Hammerle, H. F. Sailer and F. E. Weber. Effect of rhBMP-2 on guided bone regeneration in humans. 2003. | Excluded by title |
| 790 | R. Farina, A. Simonelli, L. Minenna, G. Rasperini, G. P. Schincaglia, C. Tomasi and L. Trombelli. Change in the Gingival Margin Profile After the Single Flap Approach in Periodontal Intraosseous Defects. 2015. | Excluded by title |
| 791 | R. Haas, O. Dortbudak, N. Mensdorff-Pouilly and G. Mailath. Elimination of bacteria on different implant surfaces through photosensitization and soft laser. An in vitro study. 1997. | Excluded by title |
| 792 | R. Khanna, R. Khanna, N. D. Pardhe, N. Srivastava, M. Bajpai and S. Gupta. Pure titanium membrane (Ultra – Ti®) in the treatment of periodontal osseous defects: A split-mouth comparative study. 2016. | Excluded by title |
| 793 | R. Koishi, Y. Taguchi, M. Okuda, A. Tanaka and M. Umeda. Behavior of Human Gingival Epithelial Cells on Titanium Following Abrasion of the Adjunctive Glycine Air Polishing Powder. 2016. | Excluded by title |
| 794 | R. Liu, Y. Tang, L. Zeng, Y. Zhao, Z. Ma, Z. Sun, L. Xiang, L. Ren and K. Yang. In vitro and in vivo studies of anti-bacterial copper-bearing titanium alloy for dental application. 2018. | Excluded by title |
| 795 | R. Lorenzo, V. Garcia, M. Orsini, C. Martin and M. Sanz. Clinical efficacy of a xenogeneic collagen matrix in augmenting keratinized mucosa around implants: a randomized controlled prospective clinical trial. 2012. | Excluded by title |
| 796 | R. M. Meffert. Periodontitis vs. peri-implantitis: The same disease? The same treatment?. 1996. | Excluded by title |
| 797 | R. Matthes, K. Duske, T. G. Kebede, C. Pink, R. Schluter, T. von Woedtke, K. D. Weltmann, T. Kocher and L. Jablonowski. Osteoblast growth, after cleaning of biofilm-covered titanium discs with air-polishing and cold plasma. 2017. | Excluded by title |
| 798 | R. Pokrowiecki, A. Mielczarek, T. Zareba and S. Tyski. Oral microbiome and peri-implant diseases: where are we now?. 2017. | Excluded by title |
| 799 | R. Pokrowiecki. The paradigm shift for drug delivery systems for oral and maxillofacial implants. 2018. | Excluded by title |
| 800 | R. R. Resende, E. A. Fonseca, F. M. P. Tonelli, B. R. Sousa, A. K. Santos, K. N. Gomes, S. Guatimosim, A. H. Kihara and L. O. Ladeira. Scale/topography of substrates surface resembling extracellular matrix for tissue engineering. 2014. | Excluded by title |
| 801 | R. S. Fraga, L. A. A. Antunes, K. Fontes, E. C. Kuchler, N. Iorio and L. S. Antunes. Is Antimicrobial Photodynamic Therapy Effective for Microbial Load Reduction in Peri-implantitis Treatment? A Systematic Review and Meta-Analysis. 2018. | Excluded by title |
| 802 | R. Saini. Ozone therapy in dentistry: A strategic review. 2011. | Excluded by title |
| 803 | R. Smeets, A. Henningsen, O. Jung, M. Heiland, C. Hammacher and J. M. Stein. Definition, etiology, prevention and treatment of peri-implantitis - a review. 2014. | Excluded by title |
| 804 | R. T. Kao, S. Murakami and O. R. Beirne. The use of biologic mediators and tissue engineering in dentistry. 2009. | Excluded by title |
| 805 | R. T. Stramandinoli-Zanicotti, L. M. Sassi, J. L. Schussel, M. F. Torres, S. A. M. Ferreira and A. L. Carvalho. Effect of Radiotherapy on Osseointegration of Dental Implants Immediately Placed in Postextraction Sites of Minipigs Mandibles. 2014. | Excluded by title |
| 806 | R. Tejero, E. Anitua and G. Orive. Toward the biomimetic implant surface: Biopolymers on titanium-based implants for bone regeneration. 2014. | Excluded by title |
| 807 | Ragucci, G. M. and Giralt-Hernando, M. and M√©ndez-Manj√≥n, I. and Cant√≥-Nav√©s, O. and Hern√°ndez-Alfaro, F.(2020); Factors affecting implant failure and marginal bone loss of implants placed by post-graduate students: A 1-year prospective cohort study. Materials,E3 ,2041640 | Excluded by title |
| 808 | Rahim, N. S. A. E. and Ashour, A. A.(2022); Impact of Platform Switched Implants on Marginal Bone Level in Mandibular Overdentures: A Six-Year Follow-Up Longitudinal Study. Clinical, Cosmetic and Investigational Dentistry,E3 ,307-319 | Excluded by title |
| 809 | Ramesh, D. and Sridhar, S. and Siddiqui, D. A. and Valderrama, P. and Rodrigues, D. C.(2017); Detoxification of Titanium Implant Surfaces: Evaluation of Surface Morphology and Bone-Forming Cell Compatibility. Journal of Bio- and Tribo-Corrosion,E3 ,4 | Excluded by title |
| 810 | Rammelsberg, P. and Klotz, A. L.(2024); Long-term retention and survival of cemented implant-supported zirconia and metal-ceramic single crowns: A retrospective study. Clinical Oral Implants Research,E3 , | Excluded by title |
| 811 | Reinedahl, D. and Galli, S. and Albrektsson, T. and Tengvall, P. and Johansson, C. B. and Johansson, P. H. and Wennerberg, A.(2019); Aseptic ligatures induce marginal peri-implant bone loss‚Äîan 8-week trial in rabbits. Journal of Clinical Medicine,E3 ,8 | Excluded by title |
| 812 | Renvert, S. and Lindahl, C. and Jans√•ker, A. M. R. and Persson, R. G.(2011); Treatment of peri-implantitis using an Er:YAG laser or an air-abrasive device: A randomized clinical trial. Journal of Clinical Periodontology,E3 ,165-73 | Excluded by title |
| 813 | Rosu, S. and Barattini, D. F. and Murina, F. and Gafencu, M.(2023); New medical device coating mouth gel for temporary relief of teething symptoms: a pilot randomized, open-label, controlled study. Minerva pediatrics,E3 ,4514‚Äê527 | Excluded by title |
| 814 | S. B. Khoury, L. Thomas, J. D. Walters, J. F. Sheridan and B. Leblebicioglu. Early wound healing following one-stage dental implant placement with and without antibiotic prophylaxis: a pilot study. 2008. | Excluded by title |
| 815 | S. B. Qasim, M. S. Zafar, S. Najeeb, Z. Khurshid, A. H. Shah, S. Husain and I. U. Rehman. Electrospinning of chitosan-based solutions for tissue engineering and regenerative medicine. 2018. | Excluded by title |
| 816 | S. Bhatt, J. Pulpytel and F. Aref-Khonsari. Low and atmospheric plasma polymerisation of nanocoatings for bio-applications. 2015. | Excluded by title |
| 817 | S. E. Smith and T. S. Roukis. Bone and Wound Healing Augmentation with Platelet-Rich Plasma. 2009. | Excluded by title |
| 818 | S. Eick, I. Meier, F. Spoerle, P. Bender, A. Aoki, Y. Izumi, G. E. Salvi and A. Sculean. In Vitro-Activity of Er:YAG Laser in Comparison with other Treatment Modalities on Biofilm Ablation from Implant and Tooth Surfaces. 2017. | Excluded by title |
| 819 | S. Eick, N. Gloor, C. Puls, J. Zumbrunn and A. Sculean. In vitro activity of taurolidine gel on bacteria associated with periodontitis. 2016. | Excluded by title |
| 820 | S. Elangovan. Complete regeneration of peri-implantitis-induced bony defects using guided bone regeneration is unpredictable. 2013. | Excluded by title |
| 821 | S. F. Lamolle, M. Monjo, M. Rubert, H. J. Haugen, S. P. Lyngstadaas and J. E. Ellingsen. The effect of hydrofluoric acid treatment of titanium surface on nanostructural and chemical changes and the growth of MC3T3-E1 cells. 2009. | Excluded by title |
| 822 | S. Fabrizi, A. Ortiz-Vigón Carnicero and A. Bascones Martínez. ¿Cuál es la indicación y eficacia de la terapia quirúrgica regenerativa en el tratamiento de la periimplantitis?. 2014. | Excluded by title |
| 823 | S. Flury, A. Peutzfeldt, P. R. Schmidlin and A. Lussi. Exposed Dentin: Influence of Cleaning Procedures and Simulated Pulpal Pressure on Bond Strength of a Universal Adhesive System. 2017. | Excluded by title |
| 824 | S. Ghanaati, C. Herrera-Vizcaino, S. Al-Maawi, J. Lorenz, R. J. Miron, K. Nelson, F. Schwarz, J. Choukroun and R. Sader. Fifteen Years of Platelet Rich Fibrin in Dentistry and Oromaxillofacial Surgery: How High is the Level of Scientific Evidence?. 2018. | Excluded by title |
| 825 | S. H. Kim, K. H. Kim, B. M. Seo, K. T. Koo, T. I. Kim, Y. J. Seol, Y. Ku, I. C. Rhyu, C. P. Chung and Y. M. Lee. Alveolar bone regeneration by transplantation of periodontal ligament stem cells and bone marrow stem cells in a canine peri-implant defect model: A pilot study. 2009. | Excluded by title |
| 826 | S. H. Lee, E. B. Bae, S. E. Kim, Y. P. Yun, H. J. Kim, J. W. Choi, J. J. Lee and J. B. Huh. Effects of immobilizations of rhBMP-2 and/or rhPDGF-BB on titanium implant surfaces on osseointegration and bone regeneration. 2018. | Excluded by title |
| 827 | S. H. Lee, J. H. Moon, C. M. Jeong, E. B. Bae, C. E. Park, G. R. Jeon, J. J. Lee, Y. C. Jeon and J. B. Huh. The Mechanical Properties and Biometrical Effect of 3D Preformed Titanium Membrane for Guided Bone Regeneration on Alveolar Bone Defect. 2017. | Excluded by title |
| 828 | S. H. Park, W. P. Sorensen and H. L. Wang. Management and prevention of retrograde peri-implant infection from retained root tips: two case reports. 2004. | Excluded by title |
| 829 | S. Hafner, M. Ehrenfeld, E. Storz and A. Wieser. Photodynamic Inactivation of Actinomyces naeslundii in Comparison With Chlorhexidine and Polyhexanide-A New Approach for Antiseptic Treatment of Medication-Related Osteonecrosis of the Jaw?. 2016. | Excluded by title |
| 830 | S. J. Ferguson, J. D. Langhoff, K. Voelter, B. von Rechenberg, D. Scharnweber, S. Bierbaum, M. Schnabelrauch, A. R. Kautz, V. M. Frauchiger, T. L. Mueller, G. H. van Lenthe and F. Schlottig. Biomechanical comparison of different surface modifications for dental implants. 2008. | Excluded by title |
| 831 | S. J. Froum and W. C. W. Wang. Risks and Benefits of Probing Around Natural Teeth and Dental Implants. 2018. | Excluded by title |
| 832 | S. J. Renou, M. B. Guglielmotti, A. de la Torre and R. L. Cabrini. Effect of total body irradiation on peri-implant tissue reaction: an experimental study. 2001. | Excluded by title |
| 833 | S. Jepsen, F. Schwarz, L. Cordaro, J. Derks, C. H. F. Hammerle, L. J. Heitz-Mayfield, F. Hernandez-Alfaro, H. J. A. Meijer, N. Naenni, A. Ortiz-Vigon, B. Pjetursson, G. M. Raghoebar, S. Renvert, I. Rocchietta, M. Roccuzzo, I. Sanz-Sanchez, M. Simion, C. Tomasi, L. Trombelli and I. Urban. REGENERATION OF ALVEOLAR RIDGE DEFECTS. Consensus report of group 4 of the 15th European Workshop on Periodontology on Bone Regeneration. 2019. | Excluded by title |
| 834 | S. Jepsen, T. Berglundh, R. Genco, A. M. Aass, K. Demirel, J. Derks, E. Figuero, J. L. Giovannoli, M. Goldstein, F. Lambert, A. Ortiz-Vigon, I. Polyzois, G. E. Salvi, F. Schwarz, G. Serino, C. Tomasi and N. U. Zitzmann. Primary prevention of peri-implantitis: managing peri-implant mucositis. 2015. | Excluded by title |
| 835 | S. Karakoca, C. Aydin, H. Yilmaz and B. T. Bal. Survival rates and periimplant soft tissue evaluation of extraoral implants over a mean follow-up period of three years. 2008. | Excluded by title |
| 836 | S. Kotsovilis, I. K. Karoussis, M. Trianti and I. Fourmousis. Therapy of peri-implantitis: a systematic review. 2008. | Excluded by title |
| 837 | S. Kuroshima, M. Sasaki and T. Sawase. Medication-related Osteonecrosis of the Jaw: A Literature Review. 2019. | Excluded by title |
| 838 | S. L. Oh, H. J. Shiau and M. A. Reynolds. Survival of dental implants at sites after implant failure: A systematic review. 2019. | Excluded by title |
| 839 | S. Listl, N. Fruhauf, B. Dannewitz, C. Weis, Y. K. Tu, H. J. Chang and C. M. Faggion, Jr.. Cost-effectiveness of non-surgical peri-implantitis treatments. 2015. | Excluded by title |
| 840 | S. M. Heckmann, J. G. Heckmann and H. P. Weber. Clinical outcomes of three Parkinson's disease patients treated with mandibular implant overdentures. 2000. | Excluded by title |
| 841 | S. M. Heckmann, J. G. Heckmann, J. J. Linke, W. Hohenberger and A. Mombelli. Implant therapy following liver transplantation: clinical and microbiological results after 10 years. 2004. | Excluded by title |
| 842 | S. M. Lupi, A. R. Y. Baena, C. Cassinelli, G. Iviglia, M. Tallarico, M. Morra and R. R. Y. Baena. Covalently-linked hyaluronan versus acid etched titanium dental implants: A crossover RCT in humans. 2019. | Excluded by title |
| 843 | S. M. Meloni, M. Tallarico, M. Pisano, E. Xhanari and L. Canullo. Immediate Loading of Fixed Complete Denture Prosthesis Supported by 4-8 Implants Placed Using Guided Surgery: A 5-Year Prospective Study on 66 Patients with 356 Implants. 2017. | Excluded by title |
| 844 | S. M. Popescu, H. Manolea, O. A. Diaconu, V. Mercuţ, M. Scrieciu, I. T. Dascǎlu, M. J. Ţuculina, F. Obadan and F. D. Popescu. Zirconia biocompatibility in animal studies - A systematic review. 2017. | Excluded by title |
| 845 | S. Madeswaran and S. Jayachandran. Sodium bicarbonate: A review and its uses in dentistry. 2018. | Excluded by title |
| 846 | S. Millot, P. Lesclous, M. L. Colombier, L. Radoi, C. Messeca, M. Ballanger, J. L. Charrier, P. Tramba, S. Simon, A. Berrebi, F. Doguet, E. Lansac, C. Tribouilloy, G. Habib, X. Duval and B. Iung. Position paper for the evaluation and management of oral status in patients with valvular disease: Groupe de Travail Valvulopathies de la Societe Francaise de Cardiologie, Societe Frangaise de Chinirgie Orale, Societe Frangaise de Parodontologie et d'Implantologie Orale, Societe Frangaise d'Endodontie et Societe de Pathologie Infectieuse de Langue Frangaise. 2017. | Excluded by title |
| 847 | S. Overgaard. Calcium phosphate coatings for fixation of bone implants. Evaluated mechanically and histologically by stereological methods. 2001. | Excluded by title |
| 848 | S. P. Pimentel, R. C. Casarin, F. V. Ribeiro, F. R. Cirano, K. Rovaris, F. Haiter Neto and M. Z. Casati. Impact of micronutrients supplementation on bone repair around implants: microCT and counter-torque analysis in rats. 2016. | Excluded by title |
| 849 | S. P. Szafranski, A. Winkel and M. Stiesch. The use of bacteriophages to biocontrol oral biofilms. 2017. | Excluded by title |
| 850 | S. Patntirapong, P. Habibovic and P. V. Hauschka. Effects of soluble cobalt and cobalt incorporated into calcium phosphate layers on osteoclast differentiation and activation. 2009. | Excluded by title |
| 851 | S. Pina, J. M. Oliveira and R. L. Reis. Natural-based nanocomposites for bone tissue engineering and regenerative medicine: A review. 2015. | Excluded by title |
| 852 | S. Preissner, H. C. Wirtz, A. K. Tietz, S. Abu-Sirhan, S. R. Herbst, S. Hartwig, P. Pierdzioch, A. M. Schmidt-Westhausen, H. Dommisch and M. Hertel. Bactericidal efficacy of tissue tolerable plasma on microrough titanium dental implants: An in-vitro-study. 2016. | Excluded by title |
| 853 | S. R. Miranda, H. N. Filho, L. E. Marques Padovan, D. A. Ribeiro, D. Nicolielo and M. A. Matsumoto. Use of platelet-rich plasma under autogenous onlay bone grafts. 2006. | Excluded by title |
| 854 | S. Rajesh, E. Koshi, K. Philip and A. Mohan. Antimicrobial photodynamic therapy: An overview. 2011. | Excluded by title |
| 855 | S. Renvert and I. N. Polyzois. Clinical approaches to treat peri-implant mucositis and peri-implantitis. 2015. | Excluded by title |
| 856 | S. Renvert, A. M. Roos-Jansaker and N. Claffey. Non-surgical treatment of peri-implant mucositis and peri-implantitis: a literature review. 2008. | Excluded by title |
| 857 | S. Renvert, I. Polyzois and N. Claffey. Surgical therapy for the control of peri-implantitis. 2012. | Excluded by title |
| 858 | S. Renvert, I. Polyzois and R. Maguire. Re-osseointegration on previously contaminated surfaces: a systematic review. 2009. | Excluded by title |
| 859 | S. Renvert, J. Lessem, C. Lindahl and M. Svensson. Treatment of incipient peri-implant infections using topical minocycline microspheres versus topical chlorhexidine gel as an adjunct to mechanical debridement. 2004. | Excluded by title |
| 860 | S. Renvert, J. Lessem, G. Dahlen, C. Lindahl and M. Svensson. Topical minocycline microspheres versus topical chlorhexidine gel as an adjunct to mechanical debridement of incipient peri-implant infections: a randomized clinical trial. 2006. | Excluded by title |
| 861 | S. Rogers, K. Honma and T. S. Mang. Confocal fluorescence imaging to evaluate the effect of antimicrobial photodynamic therapy depth on P. gingivalis and T. denticola biofilms. 2018. | Excluded by title |
| 862 | S. Rupf, A. N. Idlibi, F. A. Marrawi, M. Hannig, A. Schubert, L. von Mueller, W. Spitzer, H. Holtmann, A. Lehmann, A. Rueppell and A. Schindler. Removing biofilms from microstructured titanium ex vivo: a novel approach using atmospheric plasma technology. 2011. | Excluded by title |
| 863 | S. S. Linsen, M. Martini and H. Stark. Long-term results of endosteal implants following radical oral cancer surgery with and without adjuvant radiation therapy. 2012. | Excluded by title |
| 864 | S. Sakka and R. Alissa. Radiographic evaluation of healing tissues of early-failing implants after therapy: a case report. 2011. | Excluded by title |
| 865 | S. Schou, P. Holmstrup, T. Jorgensen, L. T. Skovgaard, K. Stoltze, E. Hjorting-Hansen and A. Wenzel. Anorganic porous bovine-derived bone mineral (Bio-Oss) and ePTFE membrane in the treatment of peri-implantitis in cynomolgus monkeys. 2003. | Excluded by title |
| 866 | S. Shanbhag, N. Pandis, K. Mustafa, J. R. Nyengaard and A. Stavropoulos. Bone tissue engineering in oral peri-implant defects in preclinical in vivo research: A systematic review and meta-analysis. 2018. | Excluded by title |
| 867 | S. Stübinger, J. Waser, T. Hefti, A. Drechsler, M. Sidler, K. Klein, B. von Rechenberg and F. Schlottig. Evaluation of local cancellous bone amelioration by poly-l-dl-lactide copolymers to improve primary stability of dental implants: A biomechanical study in sheep. 2015. | Excluded by title |
| 868 | S. T. Lee, M. G. Subu and T. G. Kwon. Emphysema following air-powder abrasive treatment for peri-implantitis. 2018. | Excluded by title |
| 869 | S. Taschieri, A. Pigato, G. Rosano, T. Weinstein and M. Del Fabbro. Immediate post-extraction implant placement in presence of endodontic lesions. 2009. | Excluded by title |
| 870 | S. Taschieri, S. Corbella and M. Del Fabbro. Mini-invasive osteotome sinus floor elevation in partially edentulous atrophic maxilla using reduced length dental implants: interim results of a prospective study. 2014. | Excluded by title |
| 871 | S. Tomita, S. Kasai, Y. Ihara, K. Imamura, D. Kita, K. Ota, T. Kinumatsu, T. Nakagawa and A. Saito. Effects of systemic administration of sitafloxacin on subgingival microflora and antimicrobial susceptibility profile in acute periodontal lesions. 2014. | Excluded by title |
| 872 | S. V. Dorozhkin. Calcium orthophosphate cements for biomedical application. 2008. | Excluded by title |
| 873 | S. V. Dorozhkin. Calcium orthophosphate deposits: Preparation, properties and biomedical applications. 2015. | Excluded by title |
| 874 | S. V. Dorozhkin. Calcium orthophosphates in dentistry. 2013. | Excluded by title |
| 875 | S. Vervaeke, B. Collaert, S. Vandeweghe, J. Cosyn, E. Deschepper and H. De Bruyn. The effect of smoking on survival and bone loss of implants with a fluoride-modified surface: a 2-year retrospective analysis of 1106 implants placed in daily practice. 2012. | Excluded by title |
| 876 | S. Windael, S. Vervaeke, L. Wijnen, W. Jacquet, H. De Bruyn and B. Collaert. Ten-year follow-up of dental implants used for immediate loading in the edentulous mandible: A prospective clinical study. 2018. | Excluded by title |
| 877 | S. Y. Yoo, S. K. Kim, S. J. Heo, J. Y. Koak, J. H. Lee and J. M. Park. Biochemical responses of anodized titanium implants with a poly(lactide-co-glycolide)/bone morphogenic protein-2 submicron particle coating. part 1: An in vitro study. 2015. | Excluded by title |
| 878 | Sampaio-Fernandes, M. and Vaz, P. C. and Braga, A. C. and Figueiral, M. H.(2015); IL1RN gene polymorphism in a Portuguese population with implant-supported overdentures - An observational study. Revista Portuguesa de Estomatologia, Medicina Dentaria e Cirurgia Maxilofacial,E3 ,4207-214 | Excluded by title |
| 879 | Sangkhamanee, S. S. and Teparat-Burana, T.(2024); Correction of Peri-Implant Soft Tissue Fenestration with Bony Dehiscence Associated with Intrabony Defect: A 2-Year Case Report. Case Reports in Dentistry,E3 , | Excluded by title |
| 880 | Santhosh, V. C. and Karishma and Khader, A. A. and Ramachandra, V. and Singh, R. and Shetty, B. K. and Nimbalkar, V. K.(2023); Effect of periostin in peri-implant sulcular fluid and gingival crevicular fluid: A comparative study. Ann Afr Med,E3 ,4465-469 | Excluded by title |
| 881 | Santos, P. S. D. and Granzotto, F. C. N. and Antunes, H. S. and de Lima, E. M. and Varanda, R. D. and Maccari, K. and Bezinelli, L. M. and Melo, W. R. and Soares, L. A. V., Jr. and De Macedo, L. D. and Eduardo, F. D.(2023); Dentistry consensus on HSCT - Part III: Special topics - Dentistry on HSCT. Hematology Transfusion and Cell Therapy,E3 ,3379-386 | Excluded by title |
| 882 | Satpathy, A. and Grover, V. and Kumar, A. and Jain, A. and Gopalakrishnan, D. and Grover, H. S. and Kolte, A. and Melath, A. and Khatri, M. and Dani, N. and Thakur, R. and Tiwari, V. and Yadav, V. S. and Thomas, B. and Chahal, G. S. and Bhasin, M. T. and Pandit, N. and Lawande, S. A. and Manjunath, R. G. S. and Sachdeva, S. and Bhardwaj, A. and Pradeep, A. R. and Nichani, A. S. and Singh, B. and Ganesh, P. R. and Deshpande, N. C. and Reddy, S. S. P. and Raj, S. C.(2024); Indian Society of Periodontology Good Clinical Practice Recommendations for Peri-implant Care. Journal of Indian Society of Periodontology,E3 ,143252 | Excluded by title |
| 883 | Sbricoli, L. and Cecoro, G. and Leone, C. and Nastri, L. and Guazzo, R. and Guida, L. and Annunziata, M.(2021); Bacterial adhesion to grade 4 and grade 5 turned and mildly acid-etched titanium implant surfaces: An in vitro and ex vivo study. Applied Sciences (Switzerland),E3 ,16 | Excluded by title |
| 884 | Schwarz, F. and Becker, J.(2015); Peri-implant diseases: An update on the epidemiology, etiology, diagnosis, prevention, and therapy. Implantologie,E3 ,3247-259 | Excluded by title |
| 885 | Sciuca, A. M. and Neamtu, M. and Marcu, D. and Costan, V. V. and Popa, C.(2024); ORAL MUCOSITIS IN PATIENTS WITH CHEMOTHERAPY TREATMENT. Romanian Journal of Oral Rehabilitation,E3 ,1452-460 | Excluded by title |
| 886 | Setti, P. and Pesce, P. and Dellepiane, E. and Bagnasco, F. and Zunino, P. and Menini, M.(2020); Angled implant brush for hygienic maintenance of full-arch fixed-implant rehabilitations: a pilot study. Journal of Periodontal and Implant Science,E3 ,5340-354 | Excluded by title |
| 887 | Shakibaie, B. and Sabri, H. and Blatz, M. B. and Barootchi, S.(2023); Comparison of the minimally-invasive roll-in envelope flap technique to the holding suture technique in implant surgery: a prospective case series. Journal of esthetic and restorative dentistry,E3 ,4625‚Äê631 | Excluded by title |
| 888 | Shaya, F. and Butler, B. and Hsu, Y. T.(2023); Role of Keratinized Tissue on the Management of Peri-implantitis: A Case Report. International Journal of Periodontics & Restorative Dentistry,E3 ,4517-523 | Excluded by title |
| 889 | Sheikh, Z. and Sima, C. and Glogauer, M.(2015); Bone replacement materials and techniques used for achieving vertical alveolar bone augmentation. Materials,E3 ,62953-2993 | Excluded by title |
| 890 | Shi, B. and Yan, Q. and Wu, X.(2020); Clinical application and complications of short implants (‚â§6 mm). Journal of Prevention and Treatment for Stomatological Diseases,E3 ,3137-145 | Excluded by title |
| 891 | Shiba, T. and Komatsu, K. and Watanabe, T. and Takeuchi, Y. and Nemoto, T. and Ohsugi, Y. and Katagiri, S. and Shimogishi, M. and Marukawa, E. and Iwata, T.(2023); Peri-implantitis management by resective surgery combined with implantoplasty and Er:YAG laser irradiation, accompanied by free gingival graft: a case report. Therapeutic Advances in Chronic Disease,E3 , | Excluded by title |
| 892 | Shirazian, S. and Manifar, S. and Kazemian, A. and Pourshahidi, S. and Rashtiani, A.(2023); Effectiveness of green tea mouthwash on radiation-induced mucositis in patients with head and neck cancer: a randomised clinical trial. Australian journal of herbal and naturopathic medicine,E3 ,174‚Äê78 | Excluded by title |
| 893 | Silva, D. N. A. and Monajemzadeh, S. and Casarin, M. and Chalmers, J. and Lubben, J. and Magyar, C. E. and Tetradis, S. and Pirih, F. Q.(2024); Diabetes mellitus exacerbates inflammation in a murine model of ligature-induced peri-implantitis: A histological and microtomographic study. Journal of Clinical Periodontology,E3 , | Excluded by title |
| 894 | Skora, P. and Jepsen, K.(2020); Surgical treatment of peri-implantitis. Implantologie,E3 ,2129-143 | Excluded by title |
| 895 | Sledzinska, A. and Sledzinska, P. and Bebyn, M. and Komisarek, O.(2023); Chemotherapy-Induced Oral Complications and Prophylaxis Strategies. Cancer Investigation,E3 ,5432-455 | Excluded by title |
| 896 | Srivastava, R. and Sarkar, S. and Gupta, P. and Pradhan, D. and Bagde, H. and Dhopte, A.(2024); Efficacy of Topical Turmeric, Calendula officinalis, and Tulsi (Ayurvedic Gel) in the Management of Erosive Lichen Planus: a Placebo‚ÄëControlled Study. Journal of pharmacy & bioallied sciences,E3 ,S871‚ÄêS873 | Excluded by title |
| 897 | Storelli, S. and Palandrani, G. and Manfredi, B. and Romeo, E. and De Martis, D. and Todaro, C. and Baena, R. and Lupi, S. M.(2023); Difference of Two Antiseptic Gels for the Treatment of Peri-Implant Mucositis on Plaque Index and Bleeding Score: A Randomized Controlled Clinical Study. Applied Sciences-Basel,E3 ,311 | Excluded by title |
| 898 | Sun, J. S. and Liu, K. C. and Hung, M. C. and Lin, H. Y. and Chuang, S. L. and Lin, P. J. and Chang, J. Z. C.(2023); A cross-sectional study for prevalence and risk factors of peri-implant marginal bone loss. Journal of Prosthetic Dentistry,E3 , | Excluded by title |
| 899 | T E Rams 1, T W Roberts, D Feik, A K Molzan, J Slots. Clinical and microbiological findings on newly inserted hydroxyapatite-coated and pure titanium human dental implants. 1991. | Excluded by title |
| 900 | T. Abduljabbar. Effect of mechanical debridement with adjunct antimicrobial photodynamic therapy in the treatment of peri-implant diseases in type-2 diabetic smokers and non-smokers. 2017. | Excluded by title |
| 901 | T. Abduljabbar. Effect of mechanical debridement with and without adjunct antimicrobial photodynamic therapy in the treatment of peri-implant diseases in prediabetic patients. 2017. | Excluded by title |
| 902 | T. E. Donovan, R. Marzola, K. R. Murphy, D. R. Cagna, F. Eichmiller, J. R. McKee, J. E. Metz, J. P. Albouy and M. Troeltzsch. Annual review of selected scientific literature: Report of the committee on scientific investigation of the American Academy of Restorative Dentistry. 2017. | Excluded by title |
| 903 | T. E. Van Dyke. The impact of genotypes and immune reactivity on peri-implant inflammation: Identification and therapeutic use of anti-inflammatory drugs and immunomodulators. 2012. | Excluded by title |
| 904 | T. Flemmig, W. Giannobile, P. McClain, W. Aldredge and M. Lynch. Multi-center six-month study on ARESTIN for adjunctive treatment of peri-implantitis. 2011. | Excluded by title |
| 905 | T. Goslinski, K. Konopka, J. Piskorz, M. Kryjewski, M. Wierzchowski and S. Sobiak. Prospects for Photodynamic Antimicrobial Chemotherapy - PACT. 2008. | Excluded by title |
| 906 | T. J. Gilbert, A. A. Anoushiravani, Z. Sayeed, M. C. Chambers, M. M. El-Othmani and K. J. Saleh. Osteolysis complicating total knee arthroplasty. 2016. | Excluded by title |
| 907 | T. Kano Dr, R. Yamamoto, A. Miyashita, K. Komatsu, T. Hayakawa, M. Sato and S. Oida. Regeneration of periodontal ligament for apatite-coated tooth-shaped titanium implants with and without occlusion using rat molar model. 2012. | Excluded by title |
| 908 | T. Kawasaki, S. Ohba, Y. Nakatani and I. Asahina. Clinical study of guided bone regeneration with resorbable polylactide-co-glycolide acid membrane. 2018. | Excluded by title |
| 909 | T. L. Aghaloo and A. D. Le. Growth factors in implant site development. 2004. | Excluded by title |
| 910 | T. L. Hedrick, J. D. Adams and R. G. Sawyer. Implant-associated infections: An overview. 2006. | Excluded by title |
| 911 | T. M. Marianetti, F. Grussu, D. Cervelli, G. Gasparini and S. Pelo. Equine pericardium membrane to prevent dorsal irregularities in rhinoplasty. 2014. | Excluded by title |
| 912 | T. M. You, B. H. Choi, J. Li, J. H. Jung, H. J. Lee, S. H. Lee and S. M. Jeong. The effect of platelet-rich plasma on bone healing around implants placed in bone defects treated with Bio-Oss: a pilot study in the dog tibia. 2007. | Excluded by title |
| 913 | T. Nguyen-Hieu, A. Borghetti and G. Aboudharam. Peri-implantitis: from diagnosis to therapeutics. 2012. | Excluded by title |
| 914 | T. Ogawa, K. Vandamme, X. Zhang, I. Naert, T. Possemiers, A. Chaudhari, K. Sasaki and J. Duyck. Stimulation of titanium implant osseointegration through high-frequency vibration loading is enhanced when applied at high acceleration. 2014. | Excluded by title |
| 915 | T. Rybaczek, S. Tangl, T. Dobsak, R. Gruber and U. Kuchler. The Effect of Parathyroid Hormone on Osseointegration in Insulin-Treated Diabetic Rats. 2015. | Excluded by title |
| 916 | T. Sorsa, L. Tjaderhane, Y. T. Konttinen, A. Lauhio, T. Salo, H. M. Lee, L. M. Golub, D. L. Brown and P. Mantyla. Matrix metalloproteinases: contribution to pathogenesis, diagnosis and treatment of periodontal inflammation. 2006. | Excluded by title |
| 917 | T. Sorsa, T. Tervahartiala, J. Leppilahti, M. Hernandez, J. Gamonal, A. M. Tuomainen, A. Lauhio, P. J. Pussinen and P. Mantyla. Collagenase-2 (MMP-8) as a point-of-care biomarker in periodontitis and cardiovascular diseases. Therapeutic response to non-antimicrobial properties of tetracyclines. 2011. | Excluded by title |
| 918 | T. Taiete, M. d. F. Monteiro, M. A. G. Palma, K. G. S. Ruiz, R. C. V. Casarin, F. H. Nociti Jr and M. Z. Casati. Regeneração óssea guiada no tratamento de peri-implantite: dois anos de acompanhamento clínico. 2016. | Excluded by title |
| 919 | T. Tsukeoka, M. Suzuki, C. Ohtsuki, Y. Tsuneizumi, J. Miyagi, A. Sugino, T. Inoue, R. Michihiro and H. Moriya. Enhanced fixation of implants by bone ingrowth to titanium fiber mesh: Effect of incorporation of hydroxyapatite powder. 2005. | Excluded by title |
| 920 | T. Wachi, T. Shuto, Y. Shinohara, Y. Matono and S. Makihira. Release of titanium ions from an implant surface and their effect on cytokine production related to alveolar bone resorption. 2015. | Excluded by title |
| 921 | T. Yuan, S. C. Guo, P. Han, C. Q. Zhang and B. F. Zeng. Applications of leukocyte- and platelet-rich plasma (L-PRP) in trauma surgery. 2012. | Excluded by title |
| 922 | Tadin, A. and Galic, S. and Gavic, L.(2023); Assessment of Color Change, Esthetic Perception, Treatment Satisfaction, and Side Effects Following the Use of Over-the-Counter Whitening Products. Acta stomatologica Croatica,E3 ,4300‚Äê315 | Excluded by title |
| 923 | Tallarico, M. and Ortensi, L. and Martinolli, M. and Casucci, A. and Ferrari, E. and Malaguti, G. and Montanari, M. and Scrascia, R. and Vaccaro, G. and Venezia, P. and Xhanari, E. and Rodriguez y Baena, R.(2018); Multicenter retrospective analysis of implant overdentures delivered with different design and attachment systems: Results between one and 17 years of follow-Up. Dentistry Journal,E3 ,4 | Excluded by title |
| 924 | Tavelli, L. and Barootchi, S.(2024); Soft tissue elasticity at teeth and implant sites. A novel outcome measure of the soft tissue phenotype. Journal of Periodontal Research,E3 , | Excluded by title |
| 925 | Tctr(2024); EFFECT OF TREATMENT MODALITY AND MUCOSAL EMERGENCE ANGLE ON PERI-IMPLANT MUCOSITIS TREATMENT OUTCOMES AND BACTERIAL PROFILE: RANDOMIZED CLINICAL TRIAL. https://trialsearch.who.int/Trial2.aspx?TrialID=TCTR20240512001,E3 , | Excluded by title |
| 926 | Thiebot, N. and Hamdani, A. and Blanchet, F. and Dame, M. and Tawfik, S. and Mbapou, E. and Kaddouh, A. A. and Alantar, A.(2022); Implant failure rate and the prevalence of associated risk factors: A 6-year retrospective observational survey. Journal of Oral Medicine and Oral Surgery,E3 ,2 | Excluded by title |
| 927 | Thoma, D. S. and Haas, R. and Sporniak-Tutak, K. and Garcia, A. and Taylor, T. D. and Tutak, M. and Pohl, V. and Haemmerle, C. H. F.(2024); Randomized controlled multi-centre study comparing shorter dental implants (6 mm) to longer dental implants (11-15 mm) in combination with sinus floor elevation procedures: 10-year data. Journal of Clinical Periodontology,E3 ,4499-509 | Excluded by title |
| 928 | Thoma, D. S. and Haas, R. and Sporniak-Tutak, K. and Garcia, A. and Taylor, T. D. and Tutak, M. and Pohl, V. and Hammerle, C. H. F.(2024); Randomized controlled multi-centre study comparing shorter dental implants (6 mm) to longer dental implants (11‚Äì15 mm) in combination with sinus floor elevation procedures: 10-year data. Journal of clinical periodontology,E3 , | Excluded by title |
| 929 | Tino, R. and Roach, M. A. and Fuentes, G. D. and Agrawal, A. and Zaid, M. and Cooper, D. J. and Bajaj, N. and Lin, R. and Xiao, L. and Mayo, L. L. and et al.(2024); Development and clinical implementation of a digital workflow utilizing 3D-printed oral stents for patients with head and neck cancer receiving radiotherapy. Oral oncology,E3 ,106944 | Excluded by title |
| 930 | Tino, R. and Roach, M. A. and Fuentes, G. D. and Agrawal, A. and Zaid, M. and Cooper, D. J. and Bajaj, N. and Lin, R. T. and Xiao, L. C. and Mayo, L. L. and Wiederhold, L. R. and Shah, S. J. and Tate, M. K. and Chronowski, G. M. and Reddy, J. P. and Mezera, M. and Mann, J. M. and Augspurger, M. and Ottum, A. O. and Chambers, M. S. and Koay, E. J.(2024); Development and clinical implementation of a digital workflow utilizing 3D-printed oral stents for patients with head and neck cancer receiving radiotherapy. Oral Oncology,E3 ,8 | Excluded by title |
| 931 | Tokg√∂z, S. E. and Bilhan, H.(2021); The influence of the implant-abutment complex on marginal bone and peri-implant conditions: A retrospective study. Journal of Advanced Prosthodontics,E3 ,146-54 | Excluded by title |
| 932 | Tomina, D. C. and Petru»õiu, »ò A. and Cri»ôan, B. and Leucu»õa, D. C. and Dinu, C. M.(2023); Influence of Periodontal Status and Prosthetic Treatment on Survival and Success Rates in Implant Therapy: A 5-Year Retrospective Follow-Up Study. Journal of Clinical Medicine,E3 ,13 | Excluded by title |
| 933 | U. D. Ramos, F. A. Suaid, U. M. E. Wikesjo, C. Susin, M. Taba, Jr. and A. B. Novaes, Jr.. Comparison between two antimicrobial protocols with or without guided bone regeneration in the treatment of peri-implantitis. A histomorphometric study in dogs. 2017. | Excluded by title |
| 934 | U. D. Ramos, F. Suaid, U. M. E. Wikesjo, C. Susin, P. C. Vital, S. L. S. de Souza, M. R. Messora, D. B. Palioto and A. B. Novaes, Jr.. Microbiologic effect of two topical anti-infective treatments on ligature-induced peri-implantitis: A pilot study in dogs. 2018. | Excluded by title |
| 935 | U. Kuchler, T. Spilka, K. Baron, S. Tangl, G. Watzek and R. Gruber. Intermittent parathyroid hormone fails to stimulate osseointegration in diabetic rats. 2011. | Excluded by title |
| 936 | U. M. Wikesjo, M. Qahash, G. Polimeni, C. Susin, R. H. Shanaman, M. D. Rohrer, J. M. Wozney and J. Hall. Alveolar ridge augmentation using implants coated with recombinant human bone morphogenetic protein-2: histologic observations. 2008. | Excluded by title |
| 937 | U. W. Jung, I. K. Lee, J. Y. Park, D. S. Thoma, C. H. Hammerle and R. E. Jung. The efficacy of BMP-2 preloaded on bone substitute or hydrogel for bone regeneration at peri-implant defects in dogs. 2015. | Excluded by title |
| 938 | Ueki, Y. and Ohshima, S. and Yokoyama, Y. and Takahashi, T. and Shodo, R. and Yamazaki, K. and Ohtaki, K. and Saijo, K. and Tanaka, R. and Togashi, T. and et al.(2024); Multicenter prospective phase II trial of concurrent chemoradiotherapy with weekly low-dose carboplatin for cisplatin-ineligible patients with advanced head and neck squamous cell carcinoma. International journal of clinical oncology,E3 ,120‚Äê26 | Excluded by title |
| 939 | Ueki, Y. and Ohshima, S. and Yokoyama, Y. and Takahashi, T. and Shodo, R. and Yamazaki, K. and Ohtaki, K. and Saijo, K. and Tanaka, R. and Togashi, T. and Sato, Y. and Takano, S. and Omata, J. and Takahashi, N. and Okabe, R. and Horii, A.(2024); Multicenter prospective phase II trial of concurrent chemoradiotherapy with weekly low-dose carboplatin for cisplatin-ineligible patients with advanced head and neck squamous cell carcinoma. International Journal of Clinical Oncology,E3 ,120-26 | Excluded by title |
| 940 | Urban, K.(2019); Conservative therapy and implantological rehabilitation with CAD/CAM manufactured prosthetics: Therapy of generalized periodontitis stage III (grade C). Implantologie,E3 ,3265-282 | Excluded by title |
| 941 | V. Goriainov, R. Cook, J. M. Latham, D. G. Dunlop and R. O. C. Oreffo. Bone and metal: An orthopaedic perspective on osseointegration of metals. 2014. | Excluded by title |
| 942 | V. John, D. Shin, A. Marlow and Y. Hamada. Peri-Implant Bone Loss and Peri-Implantitis: A Report of Three Cases and Review of the Literature. 2016. | Excluded by title |
| 943 | V. Montangero and R. Pomeraniec. Terapia antineoplásica química y radiante durante la oseointegración de implantes dentarios: caso clínico. 2005. | Excluded by title |
| 944 | V. Pedrazzi, E. C. Escobar, J. R. Cortelli, A. N. Haas, A. K. Andrade, C. M. Pannuti, E. R. Almeida, F. O. Costa, S. C. Cortelli and M. Rode Sde. Antimicrobial mouthrinse use as an adjunct method in peri-implant biofilm control. 2014. | Excluded by title |
| 945 | V. Perrotti, I. Vozza, M. Tumedei, G. Pompa, F. Iaculli and A. Quaranta. Rehabilitation of Postextractive Socket in the Premaxilla: A 12-Year Study on 27 Titanium Plasma Spray Resorbable Calcium Phosphate Coated Single Implants. 2018. | Excluded by title |
| 946 | V. Serrano Cuenca and B. Noguerol Rodrígez. Prevención y mantenimiento en la patología periodontal y periimplantaria. 2004. | Excluded by title |
| 947 | Vala, D. and Gupta, N. and Nayak, P. P. and Mehta, D. N. and Alam, M. W. and Kumar, S.(2023); Pharmaceutical Interventions in the Management of Oral Mucositis in Cancer Patients Undergoing Dental Treatments: a Randomized Controlled Trial. International journal of drug delivery technology,E3 ,3971‚Äê975 | Excluded by title |
| 948 | Velasco Ortega, E. and Jimenez Guerra, A. and Moreno Mu√±oz, J. and Ortiz Garc√≠a, I. and Rond√≥n Romero, J. L. and N√∫√±ez M√°rquez, E. and Matos Garrido, N. and Monsalve Guil, L.(2023); Long-term clinical research of treatment with dental implants. Avances en Odontoestomatologia,E3 ,341-50 | Excluded by title |
| 949 | Villa, A. and Sonis, S. T.(2023); Radiotherapy-induced severe oral mucositis: pharmacotherapies in recent and current clinical trials. Expert Opinion on Investigational Drugs,E3 ,4301-310 | Excluded by title |
| 950 | W. A. Camargo, S. Takemoto, J. W. Hoekstra, S. C. G. Leeuwenburgh, J. A. Jansen, J. van den Beucken and H. S. Alghamdi. Effect of surface alkali-based treatment of titanium implants on ability to promote in vitro mineralization and in vivo bone formation. 2017. | Excluded by title |
| 951 | W. C. Tan, M. Ong, J. Han, N. Mattheos, B. E. Pjetursson, A. Y. M. Tsai, I. Sanz, M. C. M. Wong, N. P. Lang and I. T. I. A. S. Grp. Effect of systemic antibiotics on clinical and patient-reported outcomes of implant therapy - a multicenter randomized controlled clinical trial. 2014. | Excluded by title |
| 952 | W. Chen, M. Zhi, Z. Feng, P. Gao, Y. Yuan, C. Zhang, Y. Wang and A. Dong. Sustained co-delivery of ibuprofen and basic fibroblast growth factor by thermosensitive nanoparticle hydrogel as early local treatment of peri-implantitis. 2019. | Excluded by title |
| 953 | W. D. Nordquist and D. J. Krutchkoff. Part III: crystalline fluorapatite-coated hydroxyapatite; potential use as a bacteriostatic agent for both pre-implant cases and retreatment of infected implant sites: a report of 4 cases. 2011. | Excluded by title |
| 954 | W. J. Shon, K. M. Woo, H. K. Kim, H. B. Kwon, S. Y. Shin and Y. S. Park. Time-dependent periimplant bone reaction of acidic monomer-treated injection molded zirconia implants in rabbit tibiae. 2015. | Excluded by title |
| 955 | W. Oshiro, Y. Ayukawa, I. Atsuta, A. Furuhashi, J. Yamazoe, R. Kondo, M. Sakaguchi, Y. Matsuura, Y. Tsukiyama and K. Koyano. Effects of CaCl2 hydrothermal treatment of titanium implant surfaces on early epithelial sealing. 2015. | Excluded by title |
| 956 | Wal, P. and Samal, H. B. and Khare, R. and Arora, K. and Soni, M. and Rathode, S. and Wal, A.(2023); Aloe Vera: A Potential Herb for Periodontitis Management. Current Traditional Medicine,E3 ,12 | Excluded by title |
| 957 | Wang, C. W. and Di Gianfilippo, R. and Kaciroti, N. and Ou, A. and Feng, S. W. and Wang, H. L.(2023); Stability of peri-implantitis surgical reconstructive therapy-a (>‚Äâ2 years) follow-up of a randomized clinical trial. Clinical oral investigations,E3 ,130 | Excluded by title |
| 958 | Wang, C. W. and Di Gianfilippo, R. and Kaciroti, N. and Ou, A. and Feng, S. W. and Wang, H. L.(2023); Stability of peri-implantitis surgical reconstructive therapy-a (>‚Äâ2¬†years) follow-up of a randomized clinical trial. Clin Oral Investig,E3 ,130 | Excluded by title |
| 959 | Wang, C. W. and Di Gianfilippo, R. and Kaciroti, N. and Ou, A. L. C. and Feng, S. W. and Wang, H. L.(2023); Stability of peri-implantitis surgical reconstructive therapy-a (&gt; 2 years) follow-up of a randomized clinical trial. Clinical Oral Investigations,E3 ,19 | Excluded by title |
| 960 | Wang, L. and Wang, J.(2019); Chinese consensus on diagnosis and treatment of radiation proctitis (2018). Chinese Journal of Inflammatory Bowel Diseases,E3 ,143952 | Excluded by title |
| 961 | Wang, P. and Xie, H. X. and Qu, Y. L. and Man, Y.(2010); Evidence-based treatment for a smoker with periimplantitis. Chinese Journal of Evidence-Based Medicine,E3 ,6759-762 | Excluded by title |
| 962 | Wang, S. and Chen, X. and Ling, Z. and Xie, Y. and Chen, C. and Shen, X. and He, F.(2024); Clinical and radiographic outcomes of implant-supported fixed prostheses with cantilever extension in anterior mandible: A retrospective study. Clinical Oral Implants Research,E3 , | Excluded by title |
| 963 | Webb, L. and Coleman, A. and Nixon, P.(2017); Dental implants: What have we learnt from long-Term follow-up?. Dental Update,E3 ,111023-1033 | Excluded by title |
| 964 | Wolfart, S. and Naujokat, H. and Wiltfang, J. and Kern, M.(2017); Implantology in the endentulous patients-An update after 25 years. Implantologie,E3 ,4327-344 | Excluded by title |
| 965 | wr, R. B. R.(2024); Use of tansagem, as a mouthwash, in cancer-related lesions. https://trialsearch.who.int/Trial2.aspx?TrialID=RBR-92784wr,E3 , | Excluded by title |
| 966 | Wychowa≈Ñski, P. and Nowak, M. and Miskiewicz, A. and Morawiec, T. and Woli≈Ñski, J. and Kucharski, Z. and Passarelli, P. C. and Bodnarenko, A. and Lopez, M. A.(2023); The Effectiveness of a Bioactive Healing Abutment as a Local Drug Delivery System to Impact Peri-Implant Mucositis: A Prospective Case Series Study. Pharmaceutics,E3 ,1 | Excluded by title |
| 967 | X. Chen, H. Hirt, Y. Li, S. U. Gorr and C. Aparicio. Antimicrobial GL13K peptide coatings killed and ruptured the wall of Streptococcus gordonii and prevented formation and growth of biofilms. 2014. | Excluded by title |
| 968 | X. Shi, L. Xu, T. B. Le, G. Zhou, C. Zheng, K. Tsuru and K. Ishikawa. Partial oxidation of TiN coating by hydrothermal treatment and ozone treatment to improve its osteoconductivity. 2016. | Excluded by title |
| 969 | X. Struillou, M. Rakic, Z. Badran, L. Macquigneau, C. Colombeix, P. Pilet, C. Verner, O. Gauthier, P. Weiss and A. Soueidan. The association of hydrogel and biphasic calcium phosphate in the treatment of dehiscence-type peri-implant defects: An experimental study in dogs. 2013. | Excluded by title |
| 970 | X. Xu, L. Wang, Z. Luo, Y. Ni, H. Sun, X. Gao, Y. Li, S. Zhang, Y. Li and S. Wei. Facile and Versatile Strategy for Construction of Anti-Inflammatory and Antibacterial Surfaces with Polydopamine-Mediated Liposomes Releasing Dexamethasone and Minocycline for Potential Implant Applications. 2017. | Excluded by title |
| 971 | Y. Bachour, S. P. Verweij, S. Gibbs, J. C. F. Ket, M. J. P. F. Ritt, F. B. Niessen and M. G. Mullender. The aetiopathogenesis of capsular contracture: A systematic review of the literature. 2018. | Excluded by title |
| 972 | Y. C. Cheng, B. Gao, X. H. Liu, X. H. Zhao, W. G. Sun, H. F. Ren and J. Wu. In vivo evaluation of an antibacterial coating containing halogenated furanone compound-loaded poly(L-lactic acid) nanoparticles on microarc-oxidized titanium implants. 2016. | Excluded by title |
| 973 | Y. C. Tsang, E. F. Corbet and L. J. Jin. Subgingival glycine powder air-polishing as an additional approach to nonsurgical periodontal therapy in subjects with untreated chronic periodontitis. 2018. | Excluded by title |
| 974 | Y. D. Rakhmatia, Y. Ayukawa, A. Furuhashi and K. Koyano. Carbonate apatite containing statin enhances bone formation in healing incisal extraction sockets in rats. 2018. | Excluded by title |
| 975 | Y. F. Chen, Y. J. Huang, G. B. Song, Q. B. Wan, J. Wang and Y. L. Chao. [Osteoblastic early attachment onto the surface of bioadhesive peptide modified pure titanium]. 2010. | Excluded by title |
| 976 | Y. Gabet, R. Muller, J. Levy, R. Dimarchi, M. Chorev, I. Bab and D. Kohavi. Parathyroid hormone 1-34 enhances titanium implant anchorage in low-density trabecular bone: a correlative micro-computed tomographic and biomechanical analysis. 2006. | Excluded by title |
| 977 | Y. J. Ji, Z. H. Tang, R. Wang, J. Cao, C. F. Cao and L. J. Jin. Effect of glycine powder air-polishing as an adjunct in the treatment of peri-implant mucositis: a pilot clinical trial. 2014. | Excluded by title |
| 978 | Y. Kinoshita and H. Maeda. Recent developments of functional scaffolds for craniomaxillofacial bone tissue engineering applications. 2013. | Excluded by title |
| 979 | Y. Liu, J. Lim and S. H. Teoh. Review: Development of clinically relevant scaffolds for vascularised bone tissue engineering. 2013. | Excluded by title |
| 980 | Y. Ohyama, J. Ito, V. J. Kitano, J. Shimada and Y. Hakeda. The polymethoxy flavonoid sudachitin suppresses inflammatory bone destruction by directly inhibiting osteoclastogenesis due to reduced ROS production and MAPK activation in osteoclast precursors. 2018. | Excluded by title |
| 981 | Y. Oshida, E. B. Tuna, O. Aktören and K. Gençay. Dental implant systems. 2010. | Excluded by title |
| 982 | Y. Raita, K. Komatsu, A. Nifuji, M. Sato, M. Morito and T. Hayakawa. Promotion of bone formation around alendronate-immobilized screw-type titanium implants after implantation into rat molar tooth sockets. 2014. | Excluded by title |
| 983 | Y. S. Jang, S. H. Moon, T. D. T. Nguyen, M. H. Lee, T. J. Oh, A. L. Han and T. S. Bae. In vivo bone regeneration by differently designed titanium membrane with or without surface treatment: a study in rat calvarial defects. 2019. | Excluded by title |
| 984 | Y. Su, S. Komasa, P. Li, M. Nishizaki, L. Chen, C. Terada, S. Yoshimine, H. Nishizaki and J. Okazaki. Synergistic effect of nanotopography and bioactive ions on peri-implant bone response. 2017. | Excluded by title |
| 985 | Y. T. Hsu, S. A. Mason and H. L. Wang. Biological implant complications and their management. 2014. | Excluded by title |
| 986 | Y. Y. Chang, J. S. Lee, M. S. Kim, S. H. Choi, J. K. Chai and U. W. Jung. Comparison of collagen membrane and bone substitute as a carrier for rhBMP-2 in lateral onlay graft. 2015. | Excluded by title |
| 987 | Yamamoto, E. and Nakazawa, M. and Hirai, M. and Hashimoto, Y. and Baba, S. and Hontsu, S.(); Cell adhesion ability of Œ±-tricalcium phosphate films formed on titanium substrates by an er:Yag laser deposition method: Imprecations for management of peri-implant inflammation - Key Engineering Materials. ,E3 ,157-163 | Excluded by title |
| 988 | Yildirm, T. T. and Kaya, F. A. and Yokus, B. and Colak, M. and Ozdemir, E. and Tekin, G. G. and Saribas, E. and Uysal, E.(2017); Clinical and radiographic comparison by analyzed cone beam CT between one stage and two stage dental implants. Journal of International Dental and Medical Research,E3 ,2368-373 | Excluded by title |
| 989 | Yoo, S. Y. and Kim, S. K. and Heo, S. J. and Koak, J. Y. and Jeon, H. R.(2021); New rehabilitation concept for maxillary edentulism: A clinical retrospective study of implant crown retained removable partial dentures. Journal of Clinical Medicine,E3 ,8 | Excluded by title |
| 990 | Yoon, S. W. and Kim, M. J. and Paeng, K. W. and Yu, K. A. and Lee, C. K. and Song, Y. W. and Cha, J. K. and Sanz, M. and Jung, U. W.(2020); Locally applied slow-release of minocycline microspheres in the treatment of peri-implant mucositis: An experimental in vivo study. Pharmaceutics,E3 ,741275 | Excluded by title |
| 991 | Yu, X. and Wu, C. and Wang, F. and Ni, J. and Wu, Y. and Li, C.(2024); Dimensional changes in free gingival grafts at implant sites in the reconstructed mandible: a retrospective study. Clin Oral Investig,E3 ,9467 | Excluded by title |
| 992 | Z. Badran, M. N. Abdallah, J. Torres and F. Tamimi. Platelet concentrates for bone regeneration: Current evidence and future challenges. 2018. | Excluded by title |
| 993 | Z. Guo, S. Wu, H. Li, Q. Li, G. Wu and C. Zhou. In vitro evaluation of electrospun PLGA/PLLA/PDLLA blend fibers loaded with naringin for guided bone regeneration. 2018. | Excluded by title |
| 994 | Z. Guobis, I. Pacauskiene and I. Astramskaite. General Diseases Influence on Peri-Implantitis Development: a Systematic Review. 2016. | Excluded by title |
| 995 | Z. H. Li, R. Muller and D. Ruffoni. Bone remodeling and mechanobiology around implants: Insights from small animal imaging. 2018. | Excluded by title |
| 996 | Z. Kadkhoda, Z. Amarlu, S. Eshraghi and N. Samiei. Antimicrobial effect of chlorhexidine on Aggregatibacter actinomycetemcomitans biofilms associated with peri-implantitis. 2016. | Excluded by title |
| 997 | Z. Qu, O. Andrukhov, M. Laky, C. Ulm, M. Matejka, M. Dard and X. Rausch-Fan. Effect of enamel matrix derivative on proliferation and differentiation of osteoblast cells grown on the titanium implant surface. 2011. | Excluded by title |
| 998 | Z. Sheikh, J. Qureshi, A. M. Alshahrani, H. Nassar, Y. Ikeda, M. Glogauer and B. Ganss. Collagen based barrier membranes for periodontal guided bone regeneration applications. 2017. | Excluded by title |
| 999 | Z. Sheikh, N. Hamdan, Y. Ikeda, M. Grynpas, B. Ganss and M. Glogauer. Natural graft tissues and synthetic biomaterials for periodontal and alveolar bone reconstructive applications: A review. 2017. | Excluded by title |
| 1000 | Z. Tang, C. Cao, Y. Sha, Y. Lin and X. Wang. Effects of non-surgical treatment modalities on peri-implantitis. 2002. | Excluded by title |
| 1001 | Z. Tasdemir, M. N. Oskaybas, A. B. Alkan and O. Cakmak. The effects of ozone therapy on periodontal therapy: A randomized placebo-controlled clinical trial. 2019. | Excluded by title |
| 1002 | Z. Z. Guo, H. Zhang, T. T. Zhou, X. Yang, X. Liu and J. B. Wu. [Clinical application of alveolar ridge augment of posterior mandible using modified sandwich technique with immediate implantations]. 2013. | Excluded by title |
| 1003 | Zhang, S. and Wang, M. and Jiang, T. and Zhou, Y. and Wang, Y.(2018); Roles of a new drug-delivery healing abutment in the prevention and treatment of peri-implant infections: A preliminary study. RSC Advances,E3 ,6838836-38843 | Excluded by title |
| 1004 | Zhang, Y. H. and Gao, T. Y. and Fan, X. Y.(2024); Original Minocycline hydrochloride plus metronidazole versus metronidazole alone for peri-implantitis: a comparative study. American Journal of Translational Research,E3 ,52122-2131 | Excluded by title |
| 1005 | Zhao, H. and Zhang, L. and Li, H. and Hieawy, A. and Shen, Y. and Liu, H.(2023); Gingival phenotype determination: Cutoff values, relationship between gingival and alveolar crest bone thickness at different landmarks. Journal of Dental Sciences,E3 ,41544-1552 | Excluded by title |
| 1006 | Zwicker, P. and Freitag, M. and Heidel, F. H. and Kocher, T. and Kramer, A.(2023); Antiseptic efficacy of two mouth rinses in the oral cavity to identify a suitable rinsing solution in radiation- or chemotherapy induced mucositis. Bmc Oral Health,E3 ,17 | Excluded by title |
| 1007 | B. E. Euctr (2017) Antimicrobial therapy in the surgical treatment of peri-implantitis | Excluded; ongoing |
| 1008 | N. L. Euctr (2013) Antibiotics as adjunct to surgical treatment of dental implant infections | Excluded; ongoing |
| 1009 | Nct (2015) Peri-Implantitis Surgical Treatment an RCT Study | Excluded; ongoing |
| 1010 | Nct(2024); The Effect of Different Antibiotic Protocols on Peri-implant Tissue Health. https://clinicaltrials.gov/ct2/show/NCT06378502,E3 , | Excluded; ongoing |
| 1011 | S. E. Euctr (2014) Surgical treatment of peri-implantitis with and without systemically adjunctive antibiotics | Excluded; ongoing |
| 1012 | U. Jprn (2016) Analysis of bacterial flora in peri-implant crevicular fluid and application for clinical treatment of peri-implantitis | Excluded; ongoing |
| 1013 | Daly, A. and McCracken, G.(2019); Peri-implant disease part 2: Management of peri-implant disease. Dental Update,E3 ,10986-992 | Excluded; review |
| 1014 | Nichitean, G. and Luchian, I. and Martu, I. and Diaconu-Popa, D. and Goriuc, A. and Tibeica, C. and Butnaru, O. and Bejan, O. and Tatarciuc, M. and Ursu, R. G.(2023); TOWARDS A BETTER DIAGNOSIS AND PROGNOSIS IN PERI-IMPLANT DISEASE FROM A MICROBIOLOGICAL PERSPECTIVE. A NARRATIVE REVIEW. Romanian Journal of Oral Rehabilitation,E3 ,1101-114 | Excluded; review |
| 1015 | Ramanauskaite, A., et al. (2022). "Surgical interventions for the treatment of peri-implantitis." CLINICAL IMPLANT DENTISTRY AND RELATED RESEARCH. | Excluded; review |
| 1016 | Alovisi, M. and Carossa, M. and Mandras, N. and Roana, J. and Costalonga, M. and Cavallo, L. and Pira, E. and Putzu, M. G. and Bosio, D. and Roato, I. and Mussano, F. and Scotti, N.(2022); Disinfection and Biocompatibility of Titanium Surfaces Treated with Glycine Powder Airflow and Triple Antibiotic Mixture: An In Vitro Study. Materials,E3 ,14 | Excluded; in vitro |
| 1017 | Alshammari, H. and Neilands, J. and Svens√§ter, G. and Stavropoulos, A.(2021); Antimicrobial potential of strontium hydroxide on bacteria associated with peri-implantitis. Antibiotics,E3 ,245536 | Excluded; in vitro |
| 1018 | Camps-Font, O. and Toledano-Serrabona, J. and Juiz-Camps, A. and Gil, J. and Sanchez-Garc√©s, M. A. and Figueiredo, R. and Gay-Escoda, C. and Valmaseda-Castell√≥n, E.(2023); Effect of Implantoplasty on Roughness, Fatigue and Corrosion Behavior of Narrow Diameter Dental Implants. Journal of Functional Biomaterials,E3 ,2 | Excluded; in vitro |
| 1019 | R. Parodi, G. Sontarelli and G. Carusi (1996) Application of Slow-Resorbing Collagen Membrane to Periodontal and Peri-Implant Guided Tissue Regeneration | Excluded; animal study |
| 1020 | N. De Angelis, P. Felice, M. G. Grusovin, A. Camurati and M. Esposito (2012) The effectiveness of adjunctive light-activated disinfection (LAD) in the treatment of peri-implantitis: 4-month results from a multicentre pragmatic randomised controlled trial | Excluded; case report/series |
| 1021 | Neto, J. B. C. and dos Reis, I. N. R. and Lazarin, R. O. and Naenni, N. and Villar, C. C. and Pannuti, C. M.(2024); Ten-year follow-up after peri-implantitis treatment using resective surgery: A case report. Clinical Advances in Periodontics,E3 ,9 | Excluded; case report/series |
| 1022 | Cosyn, J., et al. (2022). "A multi-centre randomized controlled trial comparing connective tissue graft with collagen matrix to increase soft tissue thickness at the buccal aspect of single implants: 1-year results." JOURNAL OF CLINICAL PERIODONTOLOGY 49(9): 911‐921. | Excluded; no peri-implantitis |
| 1023 | N. Moslemi, A. Shahnaz, A. Bahador, S. Torabi, S. Jabbari and Z. A. Oskouei (2016) Effect of Postoperative Amoxicillin on Early Bacterial Colonization of Peri-Implant Sulcus: A Randomized Controlled Clinical Trial | Excluded; no peri-implantitis |
| 1024 | R. Alissa and R. J. Oliver (2012) Influence of prognostic risk indicators on osseointegrated dental implant failure: a matched case-control analysis | Excluded; no peri-implantitis |
| 1025 | Abdulkareem, E. H. and Hammoodi, S. A. R. and Ali, M. R.(2020); Occurrence of peri-implant microflora in single vs. Two piece implants. International Medical Journal,E3 ,4476-480 | Excluded; no surgical treatment |
| 1026 | Ahn, D. H. and Kim, H. J. and Joo, J. Y. and Lee, J. Y.(2019); Prevalence and risk factors of peri-implant mucositis and peri-implantitis after at least 7 years of loading. Journal of Periodontal and Implant Science,E3 ,6394-405 | Excluded; no surgical treatment |
| 1027 | Al-Dahbi, A. M. and Khelkal, I. N. and Naji, E. N.(2020); Comparison of peri-implant microbiota between healthy and diseased implants. Biochemical and Cellular Archives,E3 ,3755-3760 | Excluded; no surgical treatment |
| 1028 | Aljasser, R. N. and Alsarhan, M. A. and Alotaibi, D. H. and Aloraini, S. and Ansari, A. S. and Habib, S. R. and Zafar, M. S.(2021); Analysis of prosthetic factors affecting peri-implant health: An in vivo retrospective study. Journal of Multidisciplinary Healthcare,E3 ,1183-1191 | Excluded; no surgical treatment |
| 1029 | Daubert, D. and Lee, E. D. and Botto, A. and Eftekhar, M. and Palaiologou, A. and Kotsakis, G. A.(2023); Assessment of titanium release following non-surgical peri-implantitis treatment: A randomized clinical trial. Journal of Periodontology,E3 ,91122-1132 | Excluded; no surgical treatment |
| 1030 | De Waal, Y. C. M., et al. (2021). "Systemic antibiotic therapy as an adjunct to non-surgical peri-implantitis treatment: a single-blind RCT." JOURNAL OF CLINICAL PERIODONTOLOGY 48(7): 996‐1006. | Excluded; no surgical treatment |
| 1031 | Delucchi, F. and Pozzetti, E. and Bagnasco, F. and Pesce, P. and Baldi, D. and Pera, F. and Di Tullio, N. and Pera, P. and Menini, M.(2021); Peri-implant tissue behaviour next to different titanium surfaces: 16-year post-trial follow-up. Applied Sciences (Switzerland),E3 ,20 | Excluded; no surgical treatment |
| 1032 | Electromagnetic irradiation may be a new approach to therapy for peri-implantitis | Excluded; no surgical treatment |
| 1033 | J. Neugebauer, F. Kistler, S. Kistler, F. Vizethum, F. Moller, M. Scheer and J. E. Zoller (2011) Practical procedure and clinical experience with antimicrobial photodynamic therapy for peri-implant diseases | Excluded; no surgical treatment |
| 1034 | Park, J. S., et al. (2022). "Retrospective analysis of keratinized tissue augmentation using a xenogeneic collagen matrix for resolving peri-implant mucositis and peri-implantitis." Journal of Periodontal and Implant Science 52(5). | Excluded; no surgical treatment |
| 1035 | R. Thierbach and T. Eger (2013) Clinical outcome of a nonsurgical and surgical treatment protocol in different types of peri-implantitis: A case series | Excluded; no surgical treatment |
| 1036 | Toma, S. and Brecx, M. C. and Lasserre, J. F.(2019); Clinical evaluation of three surgical modalities in the treatment of peri-implantitis: A randomized controlled clinical trial. Journal of Clinical Medicine,E3 ,7 | Excluded; no surgical treatment |
| 1037 | Windael, S., et al. (2021). "Early peri-implant bone loss as a predictor for peri-implantitis: A 10-year prospective cohort study." Clin Implant Dent Relat Res 23(3): 298-308. | Excluded; no surgical treatment |
| 1038 | Zhang, Y. and Gao, T. and Fan, X.(2024); Minocycline hydrochloride plus metronidazole versus metronidazole alone for peri-implantitis: a comparative study. Am J Transl Res,E3 ,52122-2131 | Excluded; no surgical treatment |
| 1039 | Kikuchi, T., et al. (2022). "Longitudinal study on the effect of keratinized mucosal augmentation surrounding dental implants in preventing peri-implant bone loss." PeerJ 10. | Excluded; no antibiotics |
| 1040 | Martins, O., et al. (2022). "Implantoplasty Improves Clinical Parameters over a 2-Year Follow-Up: A Case Series." MEDICINA-LITHUANIA 58(1). | Excluded; no antibiotics |
| 1041 | Solonko, M., et al. (2022). "Efficacy of keratinized mucosal augmentation with a collagen matrix concomitant to the surgical treatment of peri-implantitis: a dual-center randomized clinical trial." CLINICAL ORAL IMPLANTS RESEARCH 33(1): 105‐119. | Excluded; no antibiotics |
| 1042 | Wagner, T. P., et al. (2021). "Surgical and non-surgical debridement for the treatment of peri-implantitis: a two-center 12-month randomized trial." CLINICAL ORAL INVESTIGATIONS 25(10): 5723-5733. | Excluded; no antibiotics |
| 1043 | Bianchini, M. A. and Kuhlkamp, L. D. and Schwarz, F. and Galarraga-Vinueza, M. E.(2024); Clinical and Radiographic Outcomes of Resective Surgery with Adjunctive Implantoplasty Over a 6-to 11-Year Follow-up: A Case Series. International Journal of Periodontics & Restorative Dentistry,E3 ,411 | Excluded; no control |
| 1044 | Derks, J., et al. (2022). "Reconstructive surgical therapy of peri-implantitis: a multicenter randomized controlled clinical trial." CLINICAL ORAL IMPLANTS RESEARCH 33(9): 921‐944. | Excluded; no control |
| 1045 | Di Gianfilippo, R. and Wang, C. W. and Xie, Y. and Kinney, J. and Sugai, J. and Giannobile, W. V. and Wang, H. L.(2023); Effect of Laser-Assisted Reconstructive Surgical Therapy of Peri-implantitis on Protein Biomarkers and Bacterial Load. Clinical oral implants research,E3 ,4393‚Äê403 | Excluded; no control |
| 1046 | Di Gianfilippo, R., et al. (2023). "Effect of laser-assisted reconstructive surgical therapy of peri-implantitis on protein biomarkers and bacterial load." CLINICAL ORAL IMPLANTS RESEARCH 34(4): 393‐403. | Excluded; no control |
| 1047 | Gershenfeld, L. and Kalos, A. and Whittle, T. and Yeung, S.(2018); Randomized clinical trial of the effects of azithromycin use in the treatment of peri-implantitis. Australian Dental Journal,E3 ,3374-381 | Excluded; no control |
| 1048 | Ichioka, Y. and Trullenque-Eriksson, A. and Ortiz-Vigon, A. and Guerrero, A. and Donati, M. and Bressan, E. and Ghensi, P. and Schaller, D. and Tomasi, C. and Karlsson, K. and et al.(2023); Factors influencing outcomes of surgical therapy of peri-implantitis: a secondary analysis of 1-year results from a randomized clinical study. Journal of clinical periodontology,E3 ,101282‚Äê1304 | Excluded; no control |
| 1049 | Isler, S. C., et al. (2022). "Efficacy of concentrated growth factor versus collagen membrane in reconstructive surgical therapy of peri-implantitis: 3-year results of a randomized clinical trial." CLINICAL ORAL INVESTIGATIONS 26(8): 5247‐5260. | Excluded; no control |
| 1050 | Luengo, F., et al. (2022). "Clinical, Microbiological, and Biochemical Impact of the Surgical Treatment of Peri-Implantitis—A Prospective Case Series." JOURNAL OF CLINICAL MEDICINE 11(16). | Excluded; no control |
| 1051 | M. Roccuzzo, F. Bonino, M. Aglietta and P. Dalmasso (2012) Ten-year results of a three arms prospective cohort study on implants in periodontally compromised patients. Part 2: clinical results | Excluded; no control |
| 1052 | Monje, A., et al. (2022). "Resolution of peri-implantitis by means of implantoplasty as adjunct to surgical therapy: A retrospective study." JOURNAL OF PERIODONTOLOGY 93(1): 110-122. | Excluded; no control |
| 1053 | Monje, A., et al. (2022). "Significance of barrier membrane on the reconstructive therapy of peri-implantitis: A randomized controlled trial." JOURNAL OF PERIODONTOLOGY. | Excluded; no control |
| 1054 | Rakasevic, D. and Scepanovic, M. and Mijailovic, I. and Misic, T. and Janjic, B. and Soldatovic, I. and Markovic, A.(2023); Reconstructive Peri-Implantitis Therapy by Using Bovine Bone Substitute with or without Hyaluronic Acid: A Randomized Clinical Controlled Pilot Study. Journal of Functional Biomaterials,E3 ,315 | Excluded; no control |
| 1055 | Rakašević, D., et al. (2023). "Reconstructive Peri-Implantitis Therapy by Using Bovine Bone Substitute with or without Hyaluronic Acid: A Randomized Clinical Controlled Pilot Study." J Funct Biomater 14(3). | Excluded; no control |
| 1056 | Regidor, E., et al. (2023). "The adjunctive effect of a resorbable membrane to a xenogeneic bone replacement graft in the reconstructive surgical therapy of peri-implantitis - a randomized clinical trial." JOURNAL OF CLINICAL PERIODONTOLOGY. | Excluded; no control |
| 1057 | Renvert, S., et al. (2021). "Surgical treatment of peri-implantitis with or without a deproteinized bovine bone mineral and a native bilayer collagen membrane: a randomized clinical trial." JOURNAL OF CLINICAL PERIODONTOLOGY 48(10): 1312‐1321. | Excluded; no control |
| 1058 | Roos-Jans√•ker, A. M. and Lindahl, C. and Persson, G. R. and Renvert, S.(2011); Long-term stability of surgical bone regenerative procedures of peri-implantitis lesions in a prospective case-control study over 3 years. Journal of Clinical Periodontology,E3 ,6590-597 | Excluded; no control |
| 1059 | Successful management of peri-implantitis with a regenerative approach: a consecutive series of 51 treated implants with 3- to 7.5-year follow-up | Excluded; no control |
| 1060 | Teixeira Neves, G. S., et al. (2022). "Peri-Implant Surgical Treatment Downregulates the Expression of sTREM-1 and MMP-8 in Patients with Peri-Implantitis: A Prospective Study." Int J Environ Res Public Health 19(6). | Excluded; no control |
| 1061 | Tu, H. J., et al. (2022). "[Evaluation of the effect of Er:YAG laser combined with guided bone regeneration in the treatment of peri-implantitis with osseous defects]." Shanghai Kou Qiang Yi Xue 31(4): 418-422. | Excluded; no control |
| 1062 | Wang, C. W., et al. (2021). "Laser-assisted regenerative surgical therapy for peri-implantitis: A randomized controlled clinical trial." J Periodontol 92(3): 378-388. | Excluded; no control |
| 1063 | Wen, S. C. and Sabri, H. and Dastouri, E. and Huang, W. X. and Barootchi, S. and Wang, H. L.(2024); Submerged vs Nonsubmerged Reconstructive Approach for Surgical Treatment of Peri-implantitis: Reanalysis of Two Prospective Clinical Studies. Int J Oral Maxillofac Implants,E3 ,4526-536 | Excluded; no control |
| 1064 | Maria Lagervall, and Leif E. Jansson (2013) Treatment outcome in patients with peri-implantitis in a periodontal clinic: a retrospective study | Excluded; mixed surgical/non-surgical or with/without antibiotics |
| 1065 | Berglundh T, Wennström JL, Lindhe J. Long-term outcome of surgical treatment of peri-implantitis. A 2-11-year retrospective study. Clin Oral Implants Res. 2018 Apr;29(4):404-410. | Included |
| 1066 | Carcuac O, Derks J, Abrahamsson I, Wennström JL, Petzold M, Berglundh T. Surgical treatment of peri-implantitis: 3-year results from a randomized controlled clinical trial. J Clin Periodontol. 2017;44(12):1294-1303. | Included |
| 1067 | Carcuac O, Derks J, Charalampakis G, Abrahamsson I, Wennström J, Berglundh T. Adjunctive Systemic and Local Antimicrobial Therapy in the Surgical Treatment of Peri-implantitis: A Randomized Controlled Clinical Trial. J Dent Res. 2016;95(1):50-7. | Included |
| 1068 | Carrillo de Albornoz A, Montero E, Alonso-Español A, Sanz M, Sanz-Sánchez I. Treatment of peri-implantitis with a flapless surgical access combined with implant surface decontamination and adjunctive systemic antibiotics: A retrospective case series study. J Clin Periodontol. 2024 Aug;51(8):968-980. | Included |
| 1069 | Charalampakis G, Rabe P, Leonhardt A, Dahlén G. A follow-up study of peri-implantitis cases after treatment. J Clin Periodontol. 2011 Sep;38(9):864-71. | Included |
| 1070 | Hallström H, Persson GR, Lindgren S, Renvert S. Open flap debridement of peri-implantitis with or without adjunctive systemic antibiotics: A randomized clinical trial. J Clin Periodontol. 2017;44(12):1285-1293. | Included |
| 1071 | Riben Grundström C, Lund B, Kämpe J, Belibasakis GN, Hultin M. Systemic antibiotics in the surgical treatment of peri-implantitis: A randomized placebo-controlled trial. J Clin Periodontol. 2024;51(8):981-996. | Included |
| 1072 | Ramanauskaite A, Saltzer I, Padhye N, Begic A, Obreja K, Dahmer I, Schwarz F. Systemic Antibiotic Prophylaxis Adjunctive to Surgical Reconstructive Peri-Implantitis Treatment: A Retrospective Study. Clin Implant Dent Relat Res. 2024. doi: 10.1111/cid.13429. Epub ahead of print. | Included |

…

**Appendix 5.** Antibiotics used in the Charalampakis 2011.

|  |  | **Patient level** | **Implant level** |
| --- | --- | --- | --- |
| Antibiotic | Not specified | 1 (0.7%) | 1 (0.2%) |
|  | Penicilin V | 6 (3.9%) | 12 (2.5%) |
|  | Azithromycin | 1 (0.7%) | 2 (0.4%) |
|  | Other combination | 3 (1.9%) | 9 (1.8%) |
|  | Amoxicillin | 5 (3.2%) | 16 (3.3%) |
|  | Amoxicillin + tetracyclin | 6 (3.9%) | 19 (3.9%) |
|  | Amoxicillin + Flagyl | 62 (40.0%) | 193 (39.4%) |
|  | Flagyl | 31 (20.0%) | 108 (22.0%) |
|  | Flagyl + Penicilin V | 1 (0.7%) | 1 (0.2%) |
|  | Flagyl + Ciproxin | 2 (1.3%) | 6 (1.2%) |
|  | Flagyl + Dalacin | 1 (0.7%) | 2 (0.4%) |
|  | Doxycyclin | 9 (5.8%) | 34 (6.9%) |
|  | Tetracyclin | 4 (2.6%) | 10 (2.0%) |
|  | Amoxicillin + Clavulanic acid | 1 (0.7%) | 1 (0.2%) |
|  | Ciproxin | 17 (11.0%) | 58 (11.8%) |
|  | Flucloxacilin | 1 (0.7%) | 3 (0.6%) |
|  | Dalacin | 4 (2.6%) | 15 (3.1%) |
|  |  |  |  |
| Category | Tetracyclines (doxycycline / tetracyclin) | 13 (8.4%) | 44 (9.0%) |
|  | Penicilines (Amoxicillin / Amoxicillin + Clavulanic acid / flucloxacillin / penicillin V) | 13 (8.4%) | 32 (6.5%) |
|  | Other (Azithromycin / Ciproxin / Dalacin / Flagyl) | 53 (34.4%) | 183 (37.4%) |
|  | Combinations | 75 (48.7%) | 230 (47.0%) |

.

**Appendix 6.** Risk of bias assessment of included randomized trials with the ROB 2.0 tool.

| **Domain** | **Item** | **Carcuac 2016; 2017** | **Hallström 2017 (plus raw data)** | **Grundström 2024** |
| --- | --- | --- | --- | --- |
| **Domain 1. Randomization process** | **1.1** | Y | Y | Y |
|  | **1.2** | Y | Y | Y |
|  | **1.3** | N | N | N |
|  | **1.0 Judgement** | Low | Low | Low |
| **Domain 2. Deviations from intended interventions** | **2.1** | Y | Y | Y |
|  | **2.2** | Y | N | N |
|  | **2.3** | NA | NA | NA |
|  | **2.4** | N | N | N |
|  | **2.5** | NA | NA | NA |
|  | **2.6** | NA | NA | NA |
|  | **2.7** | NA | NA | NA |
|  | **2.0 Judgement** | Low | Low | Low |
| **Domain 3. Mising outcome data** | **3.1** | Y | Y | Y |
|  | **3.2** | NA | NA | NA |
|  | **3.3** | NA | NA | NA |
|  | **3.4** | NA | NA | NA |
|  | **3.0 judgement** | Low | Low | Low |
| **Domain 4. Measurement of the outcome** | **4.1** | N | N | N |
|  | **4.2** | N | N | N |
|  | **4.3** | N | N | N |
|  | **4.4** | NA | NA | NA |
|  | **4.5** | NA | NA | NA |
|  | **4.0 Judgement** | Low | Low | Low |
| **Domain 5. Selection of the reported result** | **5.1** | N | PN | Y |
|  | **5.2** | N | PN | N |
|  | **5.3** | Low | Low | N |
|  | **5.0 Judgement** | Low | Low | Low |
| **Overall judgement** | | Low | Low | Low |

N, no; NA, not applicable; PN, probably no; Y, yes.

**Appendix 7.** Risk of bias assessment of included non-randomized studies with the ROBINS-I tool (version 2)**.**

| **Risk of bias….** | Signalling questions | **Berglundh 2018** | **Carrillo de Albornoz 2024 (IPD)** | **Charalampakis 2011 (IPD)** | **Ramanauskaite 2024** |
| --- | --- | --- | --- | --- | --- |
| **1. due to confounding** | 1.1 Did the authors control for all the important confounding factors for which this was necessary? | **SN** | **PY** | **SN** | **PY** |
|  | 1.2 If Y/PY/WN to 1.1: Were confounding factors that were controlled for (and for which control was necessary) measured validly and reliably by the variables available in this study? | **Y** | **Y** | **NA** | **Y** |
|  | 1.3 If Y/PY/WN to 1.1: Did the authors control for any post-intervention variables that could have been affected by the intervention? | **Y** | **N** | **NA** | **N** |
|  | 1.4. Did the use of negative controls, quantitative bias analysis, or other considerations, suggest serious unmeasured confounding? | **PN** | **N** | **N** | **N** |
|  | Risk of bias judgement | **Serious** | **Low** | **Serious** | **Low** |
|  |  |  |  |  |  |
| **2. in classification of interventions** | *Questions about immortal time bias arising from definition of intervention groups* |  |  |  |  |
|  | 2.1 Did assignment of participants to the intervention group or the comparator group rely on events or measurements that occurred after the start of follow up? | **N** | **N** | **N** | **N** |
|  | 2.2 If Y/PY to 2.1: Were participants included in the comparator group until they fulfilled the definition of the intervention (or vice versa)? | **NA** | **NA** | **NA** | **NA** |
|  | *Questions about differential misclassification* |  |  |  |  |
|  | 2.3 If N/PN to 2.1: Was all information used to classify intervention and comparator groups recorded at or before the time the interventions started? | **Y** | **Y** | **Y** | **Y** |
|  | 2.4 Was classification of intervention status influenced by knowledge of the outcome or risk of the outcome? | **N** | **N** | **N** | **N** |
|  | *Question about non-differential misclassification* |  |  |  |  |
|  | 2.5 If N/PN to 2.1 and WY/N/PN/NI 2.4: Was intervention status classified correctly for all, or nearly all, participants? | **Y** | **Y** | **Y** | **Y** |
|  | Risk of bias judgement | **Low** | **Low** | **Low** | **Low** |
|  |  |  |  |  |  |
| **3. selection of participants into the study (or into the analysis)** | *A. Questions about immortal time bias arising from definition of intervention groups* |  |  |  |  |
|  | 3.1 (=2.1) Did assignment of participants to the intervention group or the comparator group rely on events or measurements that occurred after the start of follow up? | **N** | **N** | **N** | **N** |
|  | 3.2 If Y/PY to 3.1: Were participants excluded after the start of follow-up because they did not meet the definition of either the intervention or the comparator? | **NA** | **NA** | **NA** | **NA** |
|  | *B. Questions about prevalent user bias* |  |  |  |  |
|  | 3.3 Were start of follow up and start of intervention the same for most participants? | **N** | **N** | **N** | **PN** |
|  | 3.4 If N/PN to 3.3: Is the effect of intervention expected to be constant over the time period studied? | **Y** | **Y** | **Y** | **Y** |
|  | *C. Questions about other types of selection bias* |  |  |  |  |
|  | 3.5 Was selection of participants into the study (or into the analysis) based on participant characteristics observed after the start of intervention (additional to the situations addressed in 3.1 and 3.3)? | **PN** | **PN** | **PN** | **PN** |
|  | 3.6 If Y/PY to 3.5: Were the post-intervention variables that influenced selection likely to be associated with intervention? | **NA** | **NA** | **NA** | **NA** |
|  | 3.7 If Y/PY to 3.6: Were the post-intervention variables that influenced selection likely to be influenced by the outcome or a cause of the outcome? | **NA** | **NA** | **NA** | **NA** |
|  | *D. Questions about analysis, sensitivity analyses and severity of the problem* |  |  |  |  |
|  | 3.8 If Y/PY to 3.2, N/PN 3.4 or Y/PY to 3.7: Is it likely that the analysis corrected for all of the potential selection biases identified in 3.1-3.2, 3.3-3.4 or 3.5-3.7 above? | **NA** | **NA** | **NA** | **NA** |
|  | 3.9 If N/PN to 3.8: Did sensitivity analyses demonstrate that the likely impact of the potential selection biases identified in 3.1-3.2, 3.3-3.4 or 3.5-3.7 above was minimal? | **NA** | **NA** | **NA** | **NA** |
|  | 3.10 If N/PN to 3.9: Were potential selection biases identified in 3.1-3.2, 3.3-3.4 or 3.5-3.7 above sufficiently severe that the result should not be included in a quantitative synthesis? | **NA** | **NA** | **NA** | **NA** |
|  | Risk of bias judgement | **Moderate** | **Moderate** | **Moderate** | **Moderate** |
|  |  |  |  |  |  |
| **4. due to deviations from intended interventions** | 4.1 Was the study undertaken in an experimental context? | **N** | **N** | **N** | **N** |
|  | 4.2. If Y/PY to 4.1: Did participants deviate from the intended intervention as a result of the processes of recruiting and engaging them in the study? | **NA** | **NA** | **NA** | **NA** |
|  | 4.3. If Y/PY to 4.1: Did study personnel consciously or unconsciously undermine implementation of the intended interventions? | **NA** | **NA** | **NA** | **NA** |
|  | 4.4. If Y/PY/NI to 4.2 or 4.3: Were these deviations from intended intervention likely to have affected the outcome? | **NA** | **NA** | **NA** | **NA** |
|  | 4.5. Was an appropriate analysis used to estimate the effect of assignment to intervention? | **PY** | **PY** | **PY** | **PY** |
|  | Risk of bias judgement | **Low** | **Low** | **Low** | **Low** |
|  |  |  |  |  |  |
| **5. due to missing data** | 5.1 Were complete data on intervention status available for all, or nearly all, participants? | **Y** | **Y** | **Y** | **Y** |
|  | 5.2 Were complete data on the outcome available for all, or nearly all, participants? | **Y** | **Y** | **PN** | **Y** |
|  | 5.3 Were complete data on important confounding variables available for all, or nearly all, participants? | **NI** | **Y** | **NI** | **Y** |
|  | 5.4 If N/PN/NI to 5.1, 5.2 or 5.3: Is the result based on a complete case analysis? | **NI** | **NA** | **PY** | **NA** |
|  | 5.5 If Y/PY/NI to 5.4: Was exclusion from the analysis because of missing data (in intervention, confounders or the outcome) likely to be related to the true value of the outcome? | **NI** | **NA** | **NI** | **NA** |
|  | 5.6 If Y/PY/NI to 5.5: Is the relationship between the outcome and missingness likely to be explained by the variables in the analysis model? | **WN** | **NA** | **NI** | **NA** |
|  | 5.7 If N/PN to 5.4: Was the analysis based on imputing missing values? | **NA** | **NA** | **NA** | **NA** |
|  | 5.8 If Y/PY to 5.7: Is it reasonable to assume that data were ‘missing at random’ (MAR) or ‘missing completely at random’ (MCAR)? | **NA** | **NA** | **NA** | **NA** |
|  | 5.9 If Y/PY to 5.8: Was imputation performed appropriately? | **NA** | **NA** | **NA** | **NA** |
|  | 5.10 If N/PN/NI to 5.7: Was an appropriate alternative method used to correct for bias due to missing data? | **NA** | **NA** | **NA** | **NA** |
|  | 5.11 If PN/N/NI to 5.1, 5.2 or 5.3 AND (Y/PY/NI to 5.5 OR (Y/PY to 5.8 AND WN/SN/NI to 5.9) OR WN/SN/NI to 5.10): Is there evidence that the result was not biased by missing data? | **PY** | **NA** | **N** | **NA** |
|  | Risk of bias judgement | **Moderate** | **Low** | **Critical** | **Low** |
|  |  |  |  |  |  |
| **6. arising from measurement of the outcome** | 6.1 Could measurement or ascertainment of the outcome have differed between intervention groups? | **PN** | **PN** | **PN** | **PN** |
|  | 6.2 Were outcome assessors aware of the intervention received by study participants? | **Y** | **Y** | **Y** | **Y** |
|  | 6.3 If Y/PY/NI to 6.2: Could assessment of the outcome have been influenced by knowledge of the intervention received? | **PN** | **PN** | **PN** | **PN** |
|  | Risk of bias judgement | **Low** | **Low** | **Low** | **Low** |
|  |  |  |  |  |  |
| **7. in selection of the reported result** | 7.1 Was the result reported in accordance with an available, pre-determined analysis plan? | **N** | **N** | **N** | **N** |
|  | Is the numerical result being assessed likely to have been selected, on the basis of the results, from... |  |  |  |  |
|  | 7.2 ... multiple outcome *measurements* (e.g. scales, definitions, time points) within the outcome domain? | **PN** | **PN** | **PN** | **PN** |
|  | 7.3 ... multiple *analyses* of the data? | **PN** | **PN** | **PN** | **PN** |
|  | 7.4 ... multiple *subgroups*? | **PN** | **PN** | **PN** | **PN** |
|  | Risk of bias judgement | **Low** | **Low** | **Low** | **Low** |
|  | x |  |  |  |  |
|  | **Overall risk of bias** | **Serious** | **Moderate** | **Critical** | **Moderate** |

N, no; NA, not applicable; NI, no information; PN, probably no; PY, probably yes; SN, strong no; WN, weak no; Y, yes.

**Appendix 8.** Selection of confounders to adjust for, according to each study with raw data and provided outcomes. Number of confounders chosen is based on the change-in-estimate method and the sample size of each study that can support adding additional variables to the statistical model.

| **Study** | **Outcome** | **Clustering** | **Adjusted confounders** |
| --- | --- | --- | --- |
| Carrillo de Albornoz 2024 | Treatment success (short-term) | Yes | Sex, age, systemic disease smoking, periodontitis history, diabetes, hypothyroidism, anxiety / depression, SPIC compliance, toothbrush type, FMPS, jaw, deviant implant position, vestibule depth, prosthesis removal, intermediate abutment, implant surface, baseline recession, baseline SoP, baseline bone level |
|  | Treatment success (long-term) | Yes | Smoking, FMPS, deviant implant position, implant surface, baseline recession |
|  | BoP  (post-treatment) | Yes | Sex, age, systemic disease, smoking, periodontitis history, diabetes, hypothyroidism, osteoporosis, SPIC compliance, toothbrush type, FMPS, jaw, prosthesis retention, deviant implant position, vestibule depth, keratinized mucosa thickness, prosthesis removal, intermediate abutment, baseline plaque score, baseline SoP, baseline PPD, baseline bone level |
|  | SoP  (post-treatment) | Yes | Sex, systemic disease, smoking, periodontitis history, diabetes, hypothyroidism, anxiety / depression, osteoporosis, SPIC compliance, toothbrush type, FMPS, jaw, prosthesis retention, deviant implant position, vestibule depth, keratinized mucosa thickness, prosthesis removal, intermediate abutment, baseline recession, baseline BoP, baseline SoP, baseline PPD, baseline bone level |
|  | Recession change (post minus pre) | Yes | Systemic disease, keratinized mucosa thickness, baseline bone level |
|  | PPD change (post minus pre) | Yes | Sex, systemic disease, smoking, periodontitis history, hypothyroidism, toothbrush type, FMPS, jaw, prosthesis retention, prosthesis removal, baseline SoP, baseline PPD, baseline bone level |
|  | Bone level change (post minus pre) | Yes | Sex, hypothyroidism, anxiety / depression, SPIC compliance, toothbrush type, FMPS, prosthesis removal, baseline SoP, baseline PPD, baseline bone level |
|  | Bone loss ≤ 0.5 mm (post minus pre) | Yes | Sex, age, systemic disease, smoking, periodontitis history, diabetes, hypothyroidism, anxiety / depression, osteoporosis, SPIC compliance, toothbrush type, FMPS, jaw, prosthesis retention, deviant implant position, vestibule depth, keratinized mucosa thickness, prosthesis removal, intermediate abutment, implant surface, baseline recession, baseline plaque score, baseline SoP, baseline PPD, baseline bone level |
|  | PPD ≥ 5.0 mm (post-treatment) | Yes | Hypothyroidism, FMPS, vestibule depth, keratinized mucosa thickness, baseline PPD, baseline bone level |
|  | Treatment success (short-term): modified surface implants | Yes | - |
|  | Treatment success (short-term): modified surface implants | Yes | - |
|  |  |  |  |
| Charalampakis 2011 | Treatment success (short-term) | Yes | Smoking, systemic disease |
|  | Treatment success (long-term) | Yes | Follow-up duration |
|  |  |  |  |
| Grundström 2024 | Treatment success (short-term) | Yes | Smoking, implant surface, PPD < 8.0 mm |
|  |  |  |  |
| Hallström 2017 | BoP (post-treatment) | Not needed | Smoking, FMPS |
|  | PPD change (post minus pre) | Not needed | Smoking, FMPS |
|  | PPD ≥ 5.0 mm (post-treatment) | Not needed | Smoking, baseline SoP |
|  | Recession change (post minus pre) | Not needed | Smoking, FMPS |
|  | SoP  (post-treatment) | Not needed | Smoking, baseline PPD |
|  |  |  |  |
| Ramanauskaite 2024 | Treatment success (short-term) | Yes | Smoking |
|  | BoP (post-treatment) | Yes | Smoking |
|  | SoP  (post-treatment) | Yes | - |
|  | PPD ≥ 5.0 mm (post-treatment) | Yes | Smoking |
|  | PPD improvement | Yes | - |
|  | PPD change (post minus pre) | Yes | Smoking |

BoP, bleeding on probing; FMPS, full-mouth plaque score; PPD, peri-implant probing depth; SoP, suppuration on probing; SPIC, supportive peri-implant care.

**Appendix 9.** Meta-analysis on the effect of antibiotics on treatment success in the long term (at least 3 year follow-up).

AB, antibiotics group; adj, adjusted for confounders estimates used; CI, confidence interval; CT, control group (no antibiotics); n/N, events/sample; Non-RCT, not randomized comparative clinical trial; OR, odds ratio; P_SG_, P value for differences between subgroups; RCT, randomized clinical trial; SG, subgroup; REML, restricted maximum likelihood model.

**Appendix 10.** Meta-analysis on the effect of antibiotics on PPD change before and after treatment.

AB, antibiotics group; adj, adjusted for confounders estimates used; CI, confidence interval; CT, control group (no antibiotics); MD, mean difference; n/N, events/sample; Non-RCT, not randomized comparative clinical trial; PPD, peri-implant probing depth; P_SG_, P value for differences between subgroups; RCT, randomized clinical trial; SG, subgroup; REML, restricted maximum likelihood model.

**Appendix 11.** Meta-analysis on the effect of antibiotics on implants with PPD ≥ 5.0 mm post-treatment.

AB, antibiotics group; adj, adjusted for confounders estimates used; CI, confidence interval; CT, control group (no antibiotics); n/N, events/sample; Non-RCT, not randomized comparative clinical trial; OR, odds ratio; PPD, peri-implant probing depth; P_SG_, P value for differences between subgroups; RCT, randomized clinical trial; SG, subgroup; REML, restricted maximum likelihood model.

**Appendix 12.** Meta-analysis on the effect of antibiotics on RBL change before and after treatment.

AB, antibiotics group; adj, adjusted for confounders estimates used; CI, confidence interval; CT, control group (no antibiotics); MD, mean difference; n/N, events/sample; Non-RCT, not randomized comparative clinical trial; P_SG_, P value for differences between subgroups; RBL, radiographic bone level; RCT, randomized clinical trial; SG, subgroup; REML, restricted maximum likelihood model.

**Appendix 13.** Meta-analysis on the effect of antibiotics on additional RBL loss ≤ 0.5 mm before and after treatment.

AB, antibiotics group; adj, adjusted for confounders estimates used; CI, confidence interval; CT, control group (no antibiotics); n/N, events/sample; Non-RCT, not randomized comparative clinical trial; OR, odds ratio; P_SG_, P value for differences between subgroups; RBL, radiographic bone level; RCT, randomized clinical trial; SG, subgroup; REML, restricted maximum likelihood model.

**Appendix 14.** Meta-analysis on the effect of antibiotics on implants with BoP post-treatment.

AB, antibiotics group; adj, adjusted for confounders estimates used; CI, confidence interval; CT, control group (no antibiotics); n/N, events/sample; Non-RCT, not randomized comparative clinical trial; OR, odds ratio; P_SG_, P value for differences between subgroups; RCT, randomized clinical trial; SG, subgroup; REML, restricted maximum likelihood model.

**Appendix 15.** Meta-analysis on the effect of antibiotics on additional SoP post-treatment.

AB, antibiotics group; adj, adjusted for confounders estimates used; CI, confidence interval; CT, control group (no antibiotics); n/N, events/sample; Non-RCT, not randomized comparative clinical trial; OR, odds ratio; P_SG_, P value for differences between subgroups; RCT, randomized clinical trial; SG, subgroup; SoP, suppuration on probing; REML, restricted maximum likelihood model.

**Appendix 16.** Meta-analysis on the effect of antibiotics on recession change before and after treatment.

AB, antibiotics group; adj, adjusted for confounders estimates used; CI, confidence interval; CT, control group (no antibiotics); MD, mean difference; n/N, events/sample; Non-RCT, not randomized comparative clinical trial; P_SG_, P value for differences between subgroups; RCT, randomized clinical trial; SG, subgroup; REML, restricted maximum likelihood model.

**Appendix 17.** Sensitivity analysis by excluding the study of Carrillo de Albornoz 2024 that used a minimally-invasive surgical protocol. Random-effects meta-analyses for the effect of systemic antibiotics for peri-implantitis treatment (studies / effect estimate with 95% CIs and P value / heterogeneity estimates with 95% CIs / 95% predictions for chosen analysis).

| **Outcome** | **Follow-up (yr)** | **Analysis level** | **Randomized studies** | **Non-randomized studies** | **P_SG_** | **Total** |
| --- | --- | --- | --- | --- | --- | --- |
| Tx success (short-term) | 1 | Imp / Mult | 2 studies^1,2^  OR 2.29 (0.69, 7.64); P=0.18  τ^2^ 0.57 (-) / I^2^ 75% (0%, 94%) | 2 studies^3^  OR 2.66 (0.58, 12.15); P=0.21  τ^2^ 0.32 (-) / I^2^ 22% (-) | 0.88 | 4 studies^1-4^  OR 2.33 (1.06, 5.16); P=0.03  τ^2^=0.27 (0, 10.43) / I^2^=43% (0%, 81%)  PrI 0.14, 40.11 |
| Tx success (long-term) | 3 / mixed | Imp / Mult | 1 study^1^  OR 0.69 (0.32, 1.47); P=0.34  τ^2^ - / I^2^ –  PrI - | 1 study^3^  OR 5.74 (0.89, 37.00); P=0.06  τ^2^ - / I^2^ - | 0.03* | 2 studies^1,3^  OR 1.66 (0.21, 12.91); P=0.63  τ^2^=1.72 (-) / I^2^=77% (-) |
| PPD change (post-pre) | 1 / 3 / mixed | Pat / Imp | 3 studies^1,2,6^  MD -0.33 (-0.84, 0.17); P=0.19  τ^2^=0 (0, 2.47) / I^2^=0% (0%, 90%) | 2 studies^4,6^  MD 0.27 (-0.60, 1.14); P=0.55  τ^2^ 0 (-) / I^2^ 0% (-) | 0.24 | 5 studies^1,2,4-6^  MD -0.18 (-0.62, 0.25); P=0.41  τ^2^=0 (0, 1.56) / I^2^=0% (0%, 79%)  PrI -0.89, 0.25 |
| PPD ≥ 0.5 mm (post-treatment) | 1 / mixed | Imp / Mult | 2 studies^1,6^  OR 0.85 (0.37, 1.94); P=0.70  τ^2^ 0 (-) / I^2^ 0% (-) | 1 study^4^  OR 0.39 (0.06, 2.76); P=0.35  τ^2^ - / I^2^ - | 0.47 | 3 studies^1,4-5^  OR 0.76 (0.35, 1.61); P=0.47  τ^2^=0 (0, 13.13) / I^2^=0% (0%, 90%)  PrI 0.01, >100.00 |
| RBL change (post-pre) | 1 / 3 / mixed | Pat / Imp | 2 studies^1,2^  MD -0.57 (-1.02, -0.13); P=0.01  τ^2^=0.03 (-) / I^2^=24% (-) | 1 study^6^  MD 0 (-1.11, 1.11); P=1.00  τ^2^ - / I^2^ - | 0.35 | 3 studies^1,2,6^  MD -0.50 (-0.89, -0.11); P=0.01  τ^2^=0.01 (0, 6.63) / I^2^=9% (0%, 91%)  PrI -3.34, 2.34 |
| RBL loss < 0.5 mm (post-pre) | 1 / 3 | Imp | 2 studies^1,2^  OR 2.86 (1.49, 5.48); P=0.001  τ^2^=0 (-) / I^2^=0% (-) | No study | - | 2 studies^1,2^  OR 2.86 (1.49, 5.48); P=0.001  τ^2^=0 (-) / I^2^=0% (-) |
| BoP (post-treatment) | 1 / mixed | Pat / Imp | 3 studies^1,2,6^  OR 0.49 (0.24, 1.01); P=0.05  τ^2^=0.15 (0, 32.54) / I^2^=29% (0%, 93%) | 2 studies^4,6^  OR 0.46 (0.14, 1.47); P=0.19  τ^2^ 0 (-) / I^2^ 0% (-) | 0.93 | 5 studies^1,2,4-6^  OR 0.50 (0.29, 0.86); P=0.01  τ^2^=0.06 (0, 4.07) / I^2^=0% (0%, 79%)  PrI 0.16, 1.60 |
| SoP (post-treatment) | 1 / mixed | Imp / Mult | 3 studies^1,2,6^  OR 0.33 (0.18, 0.61); P<0.001  τ^2^=0 (-) / I^2^=0% (0%, 90%) | 1 study^4^  OR 2.18 (0.07, 70.04); P=0.66  τ^2^ - / I^2^ - | 0.29 | 4 studies^1,2,4-5^  OR 0.35 (0.19, 0.64); P<0.001  τ^2^=0 (0, 9.66) / I^2^=0% (0%, 85%)  PrI 0.09, 1.32 |
| Recession change (post-pre) | 1 / mixed | Imp / Mult | 2 studies^2,6^  MD 0.14 (-0.36, 0.63); P=0.59  τ^2^=0 (-) / I^2^=0% (-) | No study | - | 2 studies^2,6^  MD 0.14 (-0.36, 0.63); P=0.59  τ^2^=0 (-) / I^2^=0% (-) |

BoP, bleeding on probing; CI, confidence interval; Imp, implant; MD, mean difference; Mult, multilevel; NA, non-applicable; OR, odds ratio; Pat, patient; PPD, pocket probing depth; PrI, random-effects 95% prediction interval; P_SG_, P value between randomized/non-randomized subgroups; RBL, ragiographic bone level; SoP, suppuration on probing; Tx, treatment; yr, year.

Included studies: ^1^Carcuac 2016 / 2017; ^2^Grundstrom 2024; ^3^Charalampakis 2011; ^4^Ramanauskaite 2024; ^5^Hallström 2017; ^6^Berglundh 2018.

* statistically significant differences between subgroups for randomized and non-randomized studies (P<0.10)

**Appendix 18.** Sensitivity analysis by excluding non-randomized studies with serious or critical risk of bias according to ROBINS-I V2. Random-effects meta-analyses for the effect of systemic antibiotics for peri-implantitis treatment (studies / effect estimate with 95% CIs and P value / heterogeneity estimates with 95% CIs / 95% predictions for chosen analysis).

| **Outcome** | **Follow-up (yr)** | **Analysis level** | **Randomized studies** | **Non-randomized studies** | **P_SG_** | **Total** |
| --- | --- | --- | --- | --- | --- | --- |
| Tx success (short-term) | 1 & 1-2 | Imp / Mult | 2 studies^1,2^  OR 2.29 (0.69, 7.64); P=0.18  τ^2^ 0.57 (-) / I^2^ 75% (0%, 94%) | 2 studies^3,4^  OR 3.02 (1.25, 7.27); P=0.01  τ^2^ 0 (-) / I^2^ 0% (-) | 0.72 | 4 studies^1-4^  OR 2.54 (1.25, 5.17); P=0.01  τ^2^ 0.22 (0, 8.58) / I^2^ 43% (0%, 81%)  PrI 0.20, 32.96 |
| Tx success (long-term) | 3 & mixed | Imp / Mult | 1 study^1^  OR 0.69 (0.32, 1.47); P=0.34  τ^2^ - / I^2^ - | 1 study^3^  OR 3.64 (0.26, 50.64); P=0.34  τ^2^ - / I^2^ - | 0.23 | 2 studies^1,3^  OR 0.96 (0.26, 3.57); P=0.95  τ^2^ 0.41 (-) / I^2^ 30% (-)  PrI - |
| PPD change (post-pre) | 1 / 3 | Pat / Imp | 3 studies^1,2,5^  MD -0.33 (-0.84, 0.17); P=0.19  τ^2^ 0 (0, 2.47) / I^2^ 0% (0%, 90%) | 2 studies^3,4^  MD 0.07 (-0.58, 0.73); P=0.83  τ^2^ 0.07 (-) / I^2^ 22% (-) | 0.33 | 5 studies^1-5^  MD -0.16 (-0.51, 0.20); P=0.38  τ^2^ 0 (0, 1.56) / I^2^ 0% (0%, 79%)  PrI -0.73, 0.42 |
| PPD ≥ 0.5 mm (post-treatment) | 1 | Imp / Mult | 2 studies^1,5^  OR 0.85 (0.37, 1.94); P=0.70  τ^2^ 0 (-) / I^2^ 0% (-)  PrI - | 2 studies^3,4^  OR 0.19 (0.06, 0.56); P=0.002  τ^2^ 0 (-) / I^2^ 0% (-) | 0.03* | 4 studies^1,3-5^  OR 0.40 (0.14, 1.15); P=0.08  τ^2^ 0.58 (0, 8.87) / I^2^ 52% (0%, 84%) |
| RBL change (post-pre) | 1 / 3 | Pat / Imp | 2 studies^1,2^  MD -0.57 (-1.02, -0.13); P=0.01  τ^2^ 0.03 (-) / I^2^ 24% (-) | 1 study^3^  MD -0.19 (-0.60, 0.23); P=0.38  τ^2^ - / I^2^ - | 0.21 | 3 studies^1,2,3^  MD -0.41 (-0.76, -0.06); P=0.02  τ^2^ 0.03 (0, 4.27) / I^2^ 34% (0%, 78%)  PrI -3.57, 2.74 |
| RBL loss < 0.5 mm (post-pre) | 1 / 3 | Imp | 2 studies^1,2^  OR 2.86 (1.49, 5.48); P=0.001  τ^2^ 0 (-) / I^2^ 0% (-) | 1 study^3^  OR 2.12 (0.44, 10.23); P=0.35  τ^2^ - / I^2^ - | 0.73 | 3 studies^1,2,3^  OR 2.73 (1.50, 4.99); P=0.001  τ^2^ 0 (0, 5.48) / I^2^ 0% (0%, 90%)  PrI 0.06, >100.00 |
| BoP (post-treatment) | 1-2 | Pat / Imp | 3 studies^1,2,5^  OR 0.49 (0.24, 1.01); P=0.05  τ^2^ 0.15 (0, 32.54) / I^2^ 29% (0%, 93%) | 2 studies^3,4^  OR 0.33 (0.12, 0.90); P=0.03  τ^2^ 0 (-) / I^2^ 0% (-) | 0.53 | 6 studies^1,2,3,4,5^  OR 0.45 (0.26, 0.79); P=0.005  τ^2^ 0.08 (0, 3.14) / I^2^ 0% (0%, 79%)  PrI 0.12, 1.64 |
| SoP (post-treatment) | 1-2 | Imp / Mult | 3 studies^1,2,5^  OR 0.33 (0.18, 0.61); P<0.001  τ^2^ 0 (-) / I^2^ 0% (0%, 90%)  PrI 0.01, 17.66 | 2 studies^3,4^  OR 3.87 (0.36, 41.91); P=0.27  τ^2^ 0 (-) / I^2^ 0% (-) | 0.05* | 5 studies^1,2,3,4,5^  OR 0.38 (0.21, 0.70); P=0.002  τ^2^ 0 (0, 13.59) / I^2^ 1% (0%, 80%) |
| Recession change (post-pre) | 1-2 | Imp / Mult | 2 studies^2,5^  MD 0.14 (-0.36, 0.63); P=0.59  τ^2^ 0 (-) / I^2^ 0% (-) | 1 study^3^  MD 0.19 (0, 0.38); P=0.05  τ^2^ - / I^2^ - | 0.85 | 3 studies^2,3,5^  MD 0.18 (0, 0.36); P=0.04  τ^2^ 0 (0, 0.01) / I^2^ 0% (0%, 90%)  PrI -0.97, 1.33 |

BoP, bleeding on probing; CI, confidence interval; Imp, implant; MD, mean difference; Mult, multilevel; NA, non-applicable; OR, odds ratio; Pat, patient; PPD, pocket probing depth; PrI, random-effects 95% prediction interval; P_SG_, P value between randomized/non-randomized subgroups; RBL, ragiographic bone level; SoP, suppuration on probing; Tx, treatment; yr, year.

Included studies: ^1^Carcuac 2016 / 2017; ^2^Grundstrom 2024; ^3^Carrilo de Albornoz; ^4^Ramanauskaite 2024; ^5^Hallström 2017.

* statistically significant differences between subgroups for randomized and non-randomized studies (P<0.10).

**Appendix 19.** Detailed assessment using GRADE approach and its separate domain.

|  |  | **Reasons to downgrade** | | | | | **Reasons to upgrade** | |  |
| --- | --- | --- | --- | --- | --- | --- | --- | --- | --- |
| **Outcome**  **Studies (implants)** | **Confidence (starts from)** | **Risk of bias** | **Imprecision** | **Indirectness** | **Inconsistency** | **Publication bias** | **Dose response** | **Large effect** | **Resulting GRADE** |
| Treatment success (short-term)  5 RCTs/non-RCTs (718 implants) | Low | At least serious risk of bias for ≥1 non-RCT via ROBINS-I; however no systematic differences between RCTs / non-RCTs and sensitivity analysis showed results were robust; no downgrade | No issue | No issue | No issue | Non-applicable (<10 studies) | No evidence | No evidence | Low |
| Treatment success (long-term)  1 RCT (121 implants) | High | No issue | Downgraded by two levels for severe imprecision due to the inclusion of an inadequate sample and very wide confidence intervals. | No issue | No issue (1 study) | Non-applicable (<10 studies) | No evidence | No evidence | Low |
| PPD change (post-pre)  6 RCTs/non-RCTs (583 implants) | Low | At least serious risk of bias for ≥1 non-RCT via ROBINS-I; however no systematic differences between RCTs / non-RCTs and sensitivity analysis showed results were robust; no downgrade | No issue | No issue | Inconsistency found with heterogeneous estimates on both sides of the forest plot; but effects minor in all instances with wide 95% CIs; judged as lack of an effect; therefore, no downgrade | Non-applicable (<10 studies) | No evidence | No evidence | Low |
| PPD ≥ 5.0 mm (post-treatment)  2 RCTs (430 implants) | High | No issue | No issue | No issue | No issue | Non-applicable (<10 studies) | No evidence | No evidence | High |
| RBL change (post-pre)  4 RCTs/non-RCTs (546 implants) | Low | At least serious risk of bias for ≥1 non-RCT via ROBINS-I; however no systematic differences between RCTs / non-RCTs and sensitivity analysis showed results were robust; no downgrade | No issue | No issue | No issue | Non-applicable (<10 studies) | No evidence | No evidence | Low |
| RBL loss ≤ 0.5 mm (post-pre)  3 RCTs/non-RCTs (430 implants) | Low | No issue | No issue | No issue | No issue | Non-applicable (<10 studies) | No evidence | Potentially large effect (OR>2.50); decided to upgrade by one level. | Moderate |
| BoP (post-treatment)  6 RCTs/non-RCTs (643 implants) | Low | At least serious risk of bias for ≥1 non-RCT via ROBINS-I; however no systematic differences between RCTs / non-RCTs and sensitivity analysis showed results were robust; no downgrade | No issue | No issue | No issue | Non-applicable (<10 studies) | No evidence | No evidence | Low |
| SoP (post-treatment)  3 RCTs (604 implants) | High | No issue | No issue | No issue | No issue | Non-applicable (<10 studies) | No evidence | Potentially large effect (OR<0.4); intent to upgrade by one level, but already at high. | High |
| Recession change (post-pre)  3 RCTs/non-RCTs (376 implants) | Low | No issue | No issue | No issue | No issue | Non-applicable (<10 studies) | No evidence | No evidence | Low |
| Adverse effect: diarrhea  1 RCT (104 implants) | High | No issue | Downgraded by two levels for severe imprecision due to the inclusion of an inadequate sample and very wide confidence intervals. | No issue | No issue | Non-applicable (<10 studies) | No evidence | Potentially large effect (since antibiotics-grouping perfectly predicts diarrhea) but already downgraded for imprecision; therefore, decision not to upgrade. | Low |

BoP, bleeding on probing; CI, confidence interval; GRADE, Grading of Recommendations Assessment, Development and Evaluation; Non-RCT, non-randomized comparative clinical trial; PPD, peri-implant probing depth; RBL, radiographic bone level; RCT, randomized clinical trial; SoP, suppuration on probing
